# Interventions to Reduce Severe Brain Injury Risk in Preterm Neonates: A Systematic Review and Meta-analysis

**DOI:** 10.1001/jamanetworkopen.2023.7473

**Published:** 2023-04-13

**Authors:** Abdul Razak, Waseemoddin Patel, Naveed Ur Rehman Durrani, Abdul Kareem Pullattayil

**Affiliations:** 1 Department of Pediatrics, Monash University, Melbourne, Victoria, Australia; 2 Monash Newborn, Monash Children’s Hospital, Melbourne, Victoria, Australia; 3 Ritchie Centre, Hudson Institute of Medical Research, Melbourne, Victoria, Australia; 4 Division of Neonatology, Department of Pediatrics, Sidra Medicine, Doha, Qatar; 5 Department of Pediatrics, Weill Cornell Medicine–Qatar, Doha, Qatar; 6 Health Sciences Library, Queen’s University, Kingston, Ontario, Canada

## Abstract

**Importance:**

Interventions to reduce severe brain injury risk are the prime focus in neonatal clinical trials.

**Objective:**

To evaluate multiple perinatal interventions across clinical settings for reducing the risk of severe intraventricular hemorrhage (sIVH) and cystic periventricular leukomalacia (cPVL) in preterm neonates.

**Data Sources:**

MEDLINE, Embase, CENTRAL (Cochrane Central Register of Controlled Trials), and CINAHL (Cumulative Index to Nursing and Allied Health Literature) databases were searched from inception until September 8, 2022, using prespecified search terms and no language restrictions.

**Study Selection:**

Randomized clinical trials (RCTs) that evaluated perinatal interventions, chosen a priori, and reported 1 or more outcomes (sIVH, cPVL, and severe brain injury) were included.

**Data Extraction and Synthesis:**

Two co-authors independently extracted the data, assessed the quality of the trials, and evaluated the certainty of the evidence using the Cochrane GRADE (Grading of Recommendations, Assessment, Development, and Evaluation) approach. Fixed-effects pairwise meta-analysis was used for data synthesis.

**Main Outcomes and Measures:**

The 3 prespecified outcomes were sIVH, cPVL, and severe brain injury.

**Results:**

A total of 221 RCTs that assessed 44 perinatal interventions (6 antenatal, 6 delivery room, and 32 neonatal) were included. Meta-analysis showed with moderate certainty that antenatal corticosteroids were associated with small reduction in sIVH risk (risk ratio [RR], 0.54 [95% CI, 0.35-0.82]; absolute risk difference [ARD], −1% [95% CI, −2% to 0%]; number needed to treat [NNT], 80 [95% CI, 48-232]), whereas indomethacin prophylaxis was associated with moderate reduction in sIVH risk (RR, 0.64 [95% CI, 0.52-0.79]; ARD, −5% [95% CI, −8% to −3%]; NNT, 20 [95% CI, 13-39]). Similarly, the meta-analysis showed with low certainty that volume-targeted ventilation was associated with large reduction in risk of sIVH (RR, 0.51 [95% CI, 0.36-0.72]; ARD, −9% [95% CI, −13% to −5%]; NNT, 11 [95% CI, 7-23]). Additionally, early erythropoiesis-stimulating agents (RR, 0.68 [95% CI, 0.57-0.83]; ARD, −3% [95% CI, −4% to −1%]; NNT, 34 [95% CI, 22-67]) and prophylactic ethamsylate (RR, 0.68 [95% CI, 0.48-0.97]; ARD, −4% [95% CI, −7% to 0%]; NNT, 26 [95% CI, 13-372]) were associated with moderate reduction in sIVH risk (low certainty). The meta-analysis also showed with low certainty that compared with delayed cord clamping, umbilical cord milking was associated with a moderate increase in sIVH risk (RR, 1.82 [95% CI, 1.03-3.21]; ARD, 3% [95% CI, 0%-6%]; NNT, −30 [95% CI, −368 to −16]).

**Conclusions and Relevance:**

Results of this study suggest that a few interventions, including antenatal corticosteroids and indomethacin prophylaxis, were associated with reduction in sIVH risk (moderate certainty), and volume-targeted ventilation, early erythropoiesis-stimulating agents, and prophylactic ethamsylate were associated with reduction in sIVH risk (low certainty) in preterm neonates. However, clinicians should carefully consider all of the critical factors that may affect applicability in these interventions, including certainty of the evidence, before applying them to clinical practice.

## Key Points

**Question:**

Which perinatal interventions associated with reducing the risk of severe intraventricular hemorrhage (sIVH) in neonates born at less than 37 weeks’ gestation?

**Findings:**

In this systematic review and meta-analysis of 221 randomized clinical trials that assessed 44 perinatal interventions, antenatal corticosteroids for lung maturation (small decrease) and indomethacin prophylaxis (moderate decrease) were found with moderate certainty to be associated with reduced risk of sIVH in preterm neonates. With low certainty, volume-targeted ventilation (large decrease), early erythropoiesis-stimulating agents (moderate decrease), and prophylactic ethamsylate (moderate decrease) were associated with reduced sIVH risk, whereas umbilical cord milking (moderate increase) was associated with increased risk of sIVH in preterm neonates.

**Meaning:**

Findings of this study suggest a few interventions were associated with reduced sIVH risk; however, clinicians need to consider all of the critical factors that may affect applicability in these interventions, including certainty of the evidence, before applying them to clinical practice.

## Teaser

This systematic review and meta-analysis examines the role of perinatal interventions in the development of severe intraventricular hemorrhage and cystic periventricular leukomalacia in neonates born before 37 weeks’ gestation.

## Introduction

Intraventricular hemorrhage (IVH) and periventricular leukomalacia (PVL) are acquired brain injuries in neonates born before 37 weeks’ gestation. Severe IVH (sIVH), often referred to as IVH with ventricular distension or periventricular hemorrhagic infarction, occurs in 7.7% of very preterm infants (gestational age <32 weeks) and 16.2% of extremely preterm infants (gestational age <28 weeks).^1^ Cystic PVL (cPVL), a type of brain injury characterized by necrosis of white matter near the lateral ventricles, occurs in 6.1% of extremely preterm infants.^2^ Both sIVH and cPVL, which are collectively recognized as severe brain injury, are detrimental to long-term neurodevelopmental outcomes,^3^ and interventions to reduce their risk in preterm neonates are of utmost importance in neonatal medicine.

Several perinatal interventions have been tested in clinical trials to reduce the risk of severe brain injury in preterm neonates. Therefore, a summary of literature focusing on the role of potential interventions is needed. To our knowledge, there has been no published systematic review of interventions or a network meta-analysis of the role that the interventions evaluated in clinical trials play in reducing the risk of severe brain injury in preterm neonates. A network meta-analysis may not be appropriate because interventions for reducing the risk of severe brain injury have been studied in diverse clinical settings and at different time points. Although a comprehensive overview of intervention reviews is appropriate for summarizing the literature, evaluating a specific question may not be helpful. In addition, some reviews may be outdated given the ongoing proliferation of newer clinical trials. Hence, rather than an overview format, we used an intervention review format to evaluate multiple perinatal interventions for reducing the risk of sIVH and cPVL in preterm neonates across clinical settings.

## Methods

We conducted this systematic review and meta-analysis following the Preferred Reporting Items for Systematic Reviews and Meta-analyses (PRISMA) reporting guideline.^4^ We registered the study protocol for the systematic review with PROSPERO (registration number CRD42020186590).

### Search Strategy and Study Selection

We performed a comprehensive systematic search of the literature using appropriate prespecified search terms in MEDLINE, Embase, CENTRAL (Cochrane Central Register of Controlled Trials), and CINAHL (Cumulative Index to Nursing and Allied Health Literature) from database inception to September 8, 2022, without any language restriction. Details of the search strategy for each database are provided in eAppendix 1 in Supplement 1. To identify relevant reports, we searched the reference lists of systematic and narrative reviews and studies that fulfilled the eligibility criteria of the present study. Additionally, we explored the *Similar articles* feature in PubMed and the *Cited by* tool in Google Scholar and Web of Science. We identified the trial registration records in CENTRAL and conference abstracts in CENTRAL and Embase.

We included randomized clinical trials (RCTs) that reported 1 or more prespecified outcomes (sIVH, cPVL, and severe brain injury) of this systematic review. We excluded observational reports, reviews, case reports, and case series. We included trials that reported outcomes in preterm neonates (<37 weeks’ gestational age) or in term and preterm neonates for whom data could be extracted. We included trials of any interventions that were chosen a priori based on discussion and consensus. One of us (A.R.) prepared a preliminary list of potential interventions after searching PubMed, Cochrane Neonatal, and Cochrane Pregnancy and Childbirth. A final list of 44 interventions was prepared based on discussion and consensus between 3 of us (A.R., W.P., and N.U.R.D.). The registered protocol and eAppendix 2 in Supplement 1 list all of the interventions evaluated in the systematic review.

### Study Outcomes and Data Extraction

The 3 prespecified outcomes were (1) sIVH, defined as hemorrhage into the ventricles with ventricular distension, intraparenchymal hemorrhage, or parenchymal hemorrhagic infarct (grade III or IV using the Papile classification), identified on cranial ultrasound any time before discharge^5^; (2) cPVL, defined as white matter injury characterized by the necrosis of white matter near the lateral ventricles (must include cystic changes), identified on cranial ultrasound any time before discharge^6^; and (3) severe brain injury, defined as the presence of either sIVH or cPVL.

One of us (A.K.P.) searched the literature in the databases and compiled a final list using a reference management software (EndNote, version X9.3.3; Thomson Reuters). Two of us (W.P. and N.U.R.D.) independently screened the titles and abstracts using the screening form (eAppendix 3 in Supplement 1) and read the short-listed full-text articles to determine their eligibility. We selected clinical trials and independently examined their population characteristics, inclusion criteria, outcomes, and risk of bias. We contacted the study authors for relevant, missing, or any unclear information. We compared the extracted data for any discrepancies and resolved discrepancies by discussion and consensus with another author (A.R.).

### Statistical Analysis

Two of us (W.P. and N.U.R.D.) independently assessed the methodological quality of included trials using the Cochrane Risk-of-Bias Tool for Randomized Trials, version 1 (Cochrane Methods) for the following domains: sequence generation, allocation concealment, blinding of participants and personnel, blinding of outcome assessment, incomplete outcome data, selective reporting, and other biases. In addition, we resolved conflicts between us through discussion and consensus with a third author (A.R.).

We examined the treatment effects in the individual trials using Review Manager 5.3 (Cochrane Collaboration) and reported Mantel-Haenszel risk ratios (RRs) with fixed 95% CIs. We performed a fixed-effects model meta-analysis to yield the pooled RR (with 95% CI) and *P* value for each outcome. We also reported the absolute risk difference (ARD) and the number needed to treat (NNT) or the number needed to harm (with 95% CI) for outcomes with significant differences. We considered a *P* < .05 to be statistically significant.

We examined heterogeneity by inspecting the forest plots. Additionally, we determined the *P* value for χ^2^ and *I*^2^ tests to detect statistical heterogeneity. We conducted sensitivity and subgroup analyses to explore the causes of substantial heterogeneity (*I*^2^ >50%) if data were available. The subgroup analysis was based on the gestational ages younger than 28 weeks and 28 weeks or older. The sensitivity analysis included only high-quality studies with low risk of bias (low risk of bias in all domains) or probably low (unclear risk of bias in 1 domain and low risk of bias in all other domains).

We assessed the risk of bias due to missing results in a synthesis by using a funnel plot and the Egger test when more than 10 studies were included for any individual meta-analysis. Three of us (A.R., N.U.R.D., and W.P.) independently assessed the certainty of evidence using the Cochrane Grading of Recommendations, Assessment, Development, and Evaluation (GRADE) approach, as outlined in the GRADE Handbook,^7^ for all 3 outcomes. To communicate the systematic review findings, we used the language for interpretation based on the GRADE informative statements outlined by Santesso et al.^8^ We considered the effect size of fewer than 20 per 1000 newborns for small, 20 to 50 per 1000 newborns for moderate, and more than 50 per 1000 newborns for large benefit or harm.

## Results

The results of the database search and study selection are provided in eFigure 1 in Supplement 1. After removing 1196 duplicates from 9983 identified records, we screened the titles and abstracts of 8787 records and found 395 articles that were relevant for full-text screening. We further determined the eligibility of 395 short-listed articles and excluded 174 articles for the reasons shown in eAppendix 4 in Supplement 1. A total of 221 RCTs were included in the final sample.^9,10,11,12,13,14,15,16,17,18,19,20,21,22,23,24,25,26,27,28,29,30,31,32,33,34,35,36,37,38,39,40,41,42,43,44,45,46,47,48,49,50,51,52,53,54,55,56,57,58,59,60,61,62,63,64,65,66,67,68,69,70,71,72,73,74,75,76,77,78,79,80,81,82,83,84,85,86,87,88,89,90,91,92,93,94,95,96,97,98,99,100,101,102,103,104,105,106,107,108,109,110,111,112,113,114,115,116,117,118,119,120,121,122,123,124,125,126,127,128,129,130,131,132,133,134,135,136,137,138,139,140,141,142,143,144,145,146,147,148,149,150,151,152,153,154,155,156,157,158,159,160,161,162,163,164,165,166,167,168,169,170,171,172,173,174,175,176,177,178,179,180,181,182,183,184,185,186,187,188,189,190,191,192,193,194,195,196,197,198,199,200,201,202,203,204,205,206,207,208,209,210,211,212,213,214,215,216,217,218,219,220,221,222,223,224,225,226,227,228,229^ These trials evaluated 44 interventions, which included 6 antenatal, 6 delivery room, and 32 neonatal interventions. Details of the included trials are included in eTable 1 in Supplement 1.

We summarized the risk-of-bias assessment in eTable 1 in Supplement 1. Bias from the randomization process varied across studies. In a few studies, blinding the participants was not feasible, and the domain was assessed as high risk. Additionally, most trials provided no information on the blinding of outcome assessment and were marked unclear. However, bias in the measurement of the outcome was not an issue in most studies. Other bias domains are reported in eTable 1 in Supplement 1. Overall, some studies were at low risk of bias in all domains; however, most trials had methodological limitations in 1 or more domains.

### Outcome: sIVH

**Figure 1.**
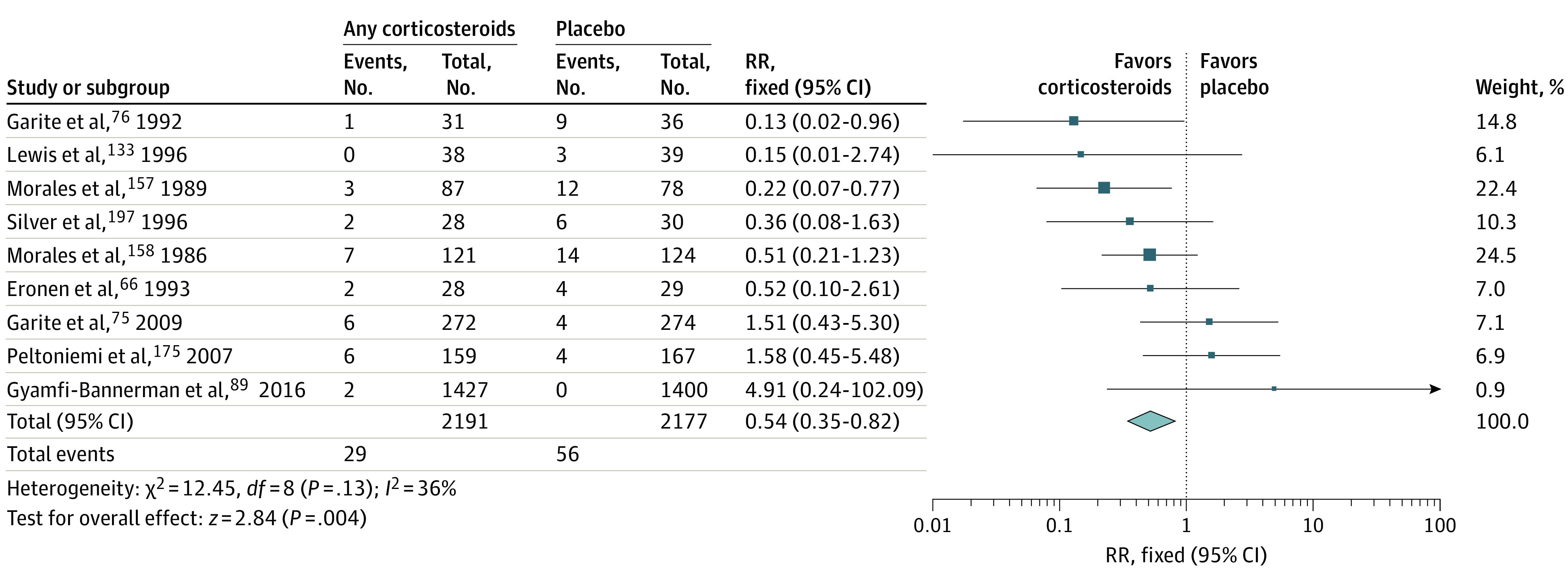
Forest Plot for Antenatal Corticosteroids for Lung Maturity vs Placebo in Preterm Neonates for the Outcome of Severe Intraventricular Hemorrhage Diamond indicates the overall effect estimate from the meta-analysis, and squares indicate a point estimate for the individual study. RR indicates risk ratio.

Meta-analysis of data from 9 trials (4368 participants)^66,75,76,89,133,157,158,175,197^ showed a small reduction in sIVH risk (RR, 0.54 [95% CI, 0.35-0.82]; *I*^2^ = 36%; ARD, −1% [95% CI, −2% to −0%]; NNT, 80 [95% CI, 48-232]) (Table and Figure 1). We assessed the certainty of evidence as moderate, which was a downgrade for serious study design limitations. Meta-analysis of data showed no treatment effect for other antenatal interventions, including betamethasone vs dexamethasone for lung maturity, repeat vs single antenatal corticosteroids, magnesium sulfate for neuroprotection or tocolysis or antibiotics for preterm premature rupture of membranes (Table; eFigures 2-5 in Supplement 1). No data were available for cesarean delivery vs vaginal delivery for preterm birth.

**Table.** Summary of Meta-analysis of Interventions on Severe Intraventricular Hemorrhage

Intervention	No. of RCTs	No. of patients	RR (95% CI)	Heterogeneity, *I^2^*, %^a^	GRADE
**Antenatal interventions**					
Antenatal corticosteroids for lung maturity	9	4368	0.54 (0.35-0.82)^b^	36	Moderate^c^
Betamethasone vs dexamethasone for lung maturity	4	1956	2.17 (0.89-5.25)	0	Moderate^c^
Repeat antenatal corticosteroids vs single-course antenatal corticosteroids	8	5472	1.06 (0.73-1.56)	13	Moderate^c^
Magnesium sulfate for neuroprotection or tocolysis	6	4559	0.80 (0.61-1.06)	10	Moderate^c^
Antibiotics for premature rupture of membranes	4	893	0.73 (0.42-1.26)	0	Low^c^^,^^d^
Cesarean delivery vs vaginal delivery for preterm birth	NA	NA	NA	NA	NA
**Delivery room interventions**					
Lower vs higher FiO_2_ for resuscitation	8	918	0.92 (0.61-1.40)	0	Moderate^c^
Sustained inflation vs standard resuscitation	10	1290	0.92 (0.67-1.26)	0	Moderate^c^
Delayed cord clamping vs early cord clamping	15	2501	0.96 (0.65-1.42)	0	Moderate^c^
Umbilical cord milking vs early cord clamping	12	1005	0.91 (0.61-1.37)	0	Moderate^c^
Umbilical cord milking vs delayed cord clamping	6	866	1.82 (1.03-3.21)^b^	27	Low^c^^,^^d^
Delayed cord clamping with respiratory support vs without respiratory support	1	150	1.33 (0.31-5.75)	NA	Low^c^^,^^d^
**Neonatal interventions**					
Supine head midline vs supine head rotated	3	290	0.71 (0.37-1.33)	0	Low^c^^,^^d^
LISA vs INSURE	6	1227	0.79 (0.52-1.20)	3	Low^c^^,^^d^
Volume-targeted vs pressure-limited ventilation	13	878	0.51 (0.36-0.72)^b^	9	Low^c^^,^^e^
Elective HFOV vs conventional ventilation	19	4196	1.11 (0.96-1.29)	16	Moderate^c^
Elective HFJV vs conventional ventilation	2	193	1.37 (0.79-2.37)	19	Low^c^^,^^d^
Oxygen saturation target after birth: 85%-89% vs 91%-95%	4	3684	0.92 (0.77-1.10)	0	High
Permissive hypercapnia vs normocapnia	5	912	0.92 (0.71-1.21)	0	Low^c^^,^^d^
Early extubation vs delayed extubation	1	86	0.32 (0.07-1.49)	NA	Low^c^^,^^d^
Caffeine prophylaxis or treatment for apnea or postextubation	3	2106	0.91 (0.72-1.16)	48	Moderate^a^
High-dose vs low-dose caffeine for apnea or postextubation	6	662	1.34 (0.74-2.41)	0	Low^c^^,^^d^
Sedation during ventilation: midazolam vs placebo	1	43	1.59 (0.43-5.84)	NA	Low^c^^,^^d^
Sedation during ventilation: opioids vs no placebo	5	1106	1.02 (0.73-1.43)	34	Moderate^c^
Sedation during ventilation: phenobarbitone vs placebo	NA	NA	NA	NA	NA
Neuromuscular paralysis during ventilation vs placebo	2	217	0.51 (0.25-1.06)	78	Very low^a^^,^^c^^,^^d^
Early erythropoiesis-stimulating agents vs placebo	12	5117	0.68 (0.57-0.83)^b^	45	Low^a^^,^^c^
Volume expansion vs inotropes (any) for hypotension	1	39	1.47 (0.96-2.25)	NA	Low^c^^,^^d^
Dopamine vs dobutamine for hypotension	2	83	0.73 (0.15-3.50)	0	Low^c^^,^^d^
Indomethacin prophylaxis for PDA vs placebo	15	2584	0.64 (0.52-0.79)^b^	0	Moderate^c^
Indomethacin presymptomatic treatment for PDA vs placebo	1	92	0.36 (0.08-1.71)	NA	Low^c^^,^^d^
Ibuprofen prophylaxis for PDA vs placebo	8	959	0.68 (0.46-1.01)	31	Low^c^^,^^d^
Ibuprofen presymptomatic treatment for PDA vs placebo	3	467	1.18 (0.75-1.88)	0	Moderate^d^
Restrictive vs liberal packed red blood cell transfusion for anemia	4	1157	0.97 (0.63-1.47)	0	Moderate^c^
Low vs high threshold for platelet transfusion for thrombocytopenia^f^	NA	NA	NA	NA	NA
Prophylactic plasma administration vs placebo	2	588	0.67 (0.37-1.24)	5	Low^c^^,^^d^
Prophylactic factor VII administration vs placebo	NA	NA	NA	NA	NA
Prophylactic antithrombin III administration vs placebo	2	175	0.92 (0.40-2.14)	0	Low^c^^,^^d^
Prophylactic ethamsylate administration vs placebo	6	1117	0.68 (0.48-0.97)^b^	2	Low^c^^,^^d^
Prophylactic heparin administration vs placebo	1	107	0.87 (0.31-2.43)	NA	Low^c^^,^^d^
Stem cell therapy	NA	NA	NA	NA	NA
Vitamin A supplementation vs placebo	7	1384	0.84 (0.65-1.07)	26	Low^c^^,^^d^
Vitamin E supplementation vs placebo	7	1095	0.96 (0.68-1.36)	29	Moderate^d^
Vitamin K supplementation vs placebo	NA	NA	NA	NA	NA

Abbreviations: FiO_2_, fraction of inspired oxygen; GRADE, Grading of Recommendations, Assessment, Development, and Evaluation; HFJV, high-frequency jet ventilation; HFOV, high-frequency oscillatory ventilation; INSURE, Intubate-surfactant-extubate; LISA, less-invasive surfactant administration; NA, not available; PDA, patent ductus arteriosus; PVL, periventricular leukomalacia; RCT, randomized clinical trial; RR, relative risk.

^a^ Heterogeneity.

^b^ Significant association.

^c^ Risk of bias.

^d^ Imprecision.

^e^ Publication bias.

^f^ Two RCTs^51,129^ were available and provided data on IVH, but a meta-analysis was not performed due to heterogeneous comparisons in the trials. No significant differences were found between the groups in all of the trials.

Meta-analysis of data from 6 trials (866 participants)^31,70,112,114,115,181^ showed a moderate increase in sIVH risk (RR, 1.82 [95% CI, 1.03-3.21]; *I*^2^ = 27%; ARD, 3% [95% CI, 0%-6%]; NNT, −30 [95% CI, −368 to −16]) with umbilical cord milking (UCM) vs delayed cord clamping (DCC) (Table; eFigure 6 in Supplement 1). We assessed the certainty of evidence as low, which was a downgrade for serious study design limitations and imprecision. Meta-analysis showed no treatment effect for other delivery room interventions, including lower vs higher fraction of inspired oxygen for resuscitation, sustained inflation vs standard resuscitation, DCC vs early cord clamping, UCM vs early cord clamping, or DCC with respiratory support vs without respiratory support (Table; eFigures 7-11 in Supplement 1).

**Figure 2.**
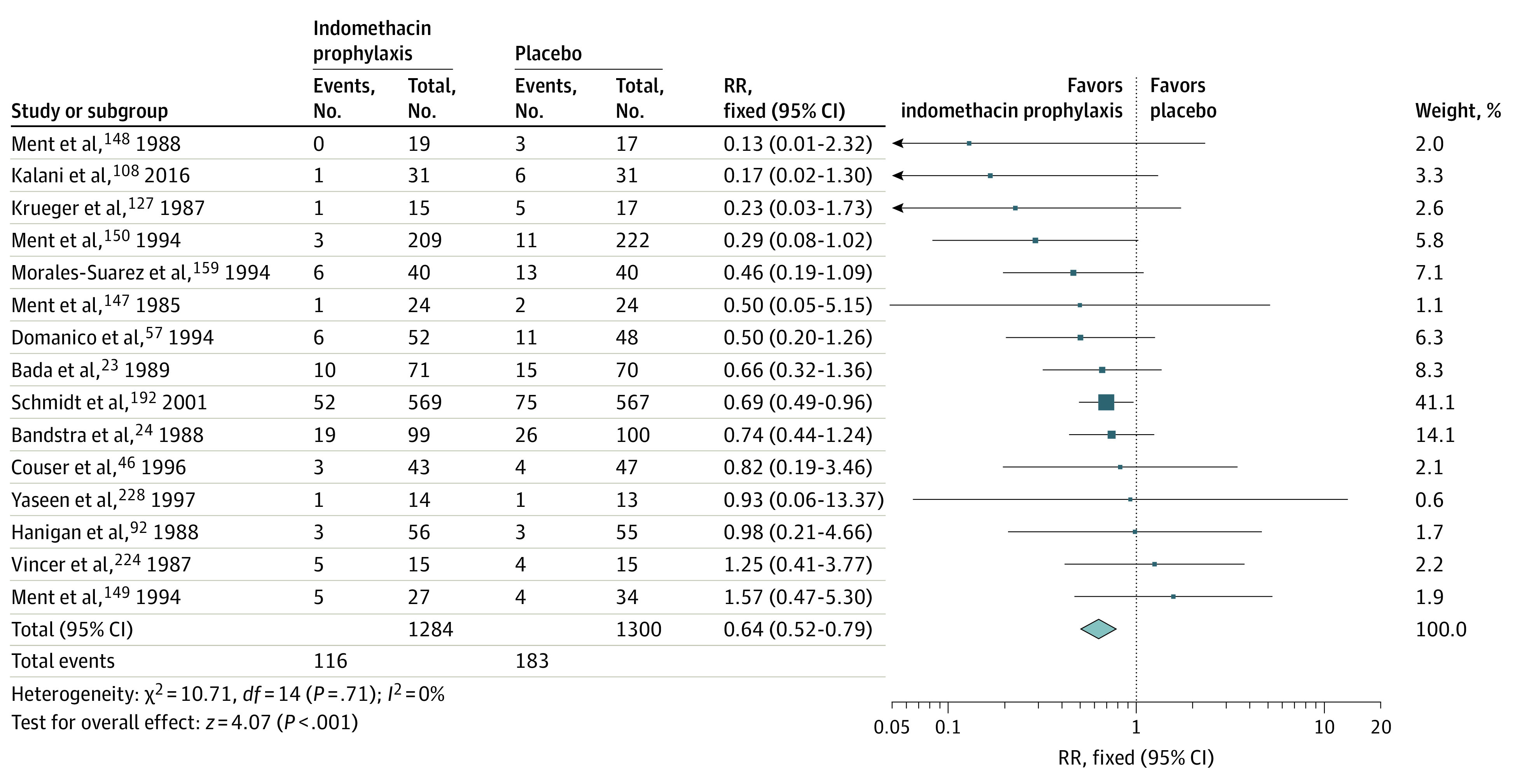
Forest Plot for Indomethacin Prophylaxis for Patent Ductus Arteriosus vs Placebo in Preterm Neonates for the Outcome of Severe Intraventricular Hemorrhage Diamond indicates the overall effect estimate from the meta-analysis, and squares indicate a point estimate for the individual study. RR indicates risk ratio.

Meta-analysis of data from 15 trials (2584 participants)^23,24,46,57,92,108,127,147,148,149,150,159,192,224,228^ showed a moderate reduction in sIVH risk (RR, 0.64 [95% CI, 0.52-0.79]; *I*^2^ = 0%; ARD, −5% [95% CI, −8% to −3%]; NNT, 20 [95% CI, 13-39]) with indomethacin prophylaxis for patent ductus arteriosus (PDA) vs placebo (Table and Figure 2). We assessed the certainty of evidence as moderate, which was a downgrade for serious study design limitations, and found no statistically significant evidence of publication bias (funnel plot: symmetrical; Egger intercept test: 1-tailed *P* = .13) (eFigures 12 and 13 in Supplement 1).

**Figure 3.**
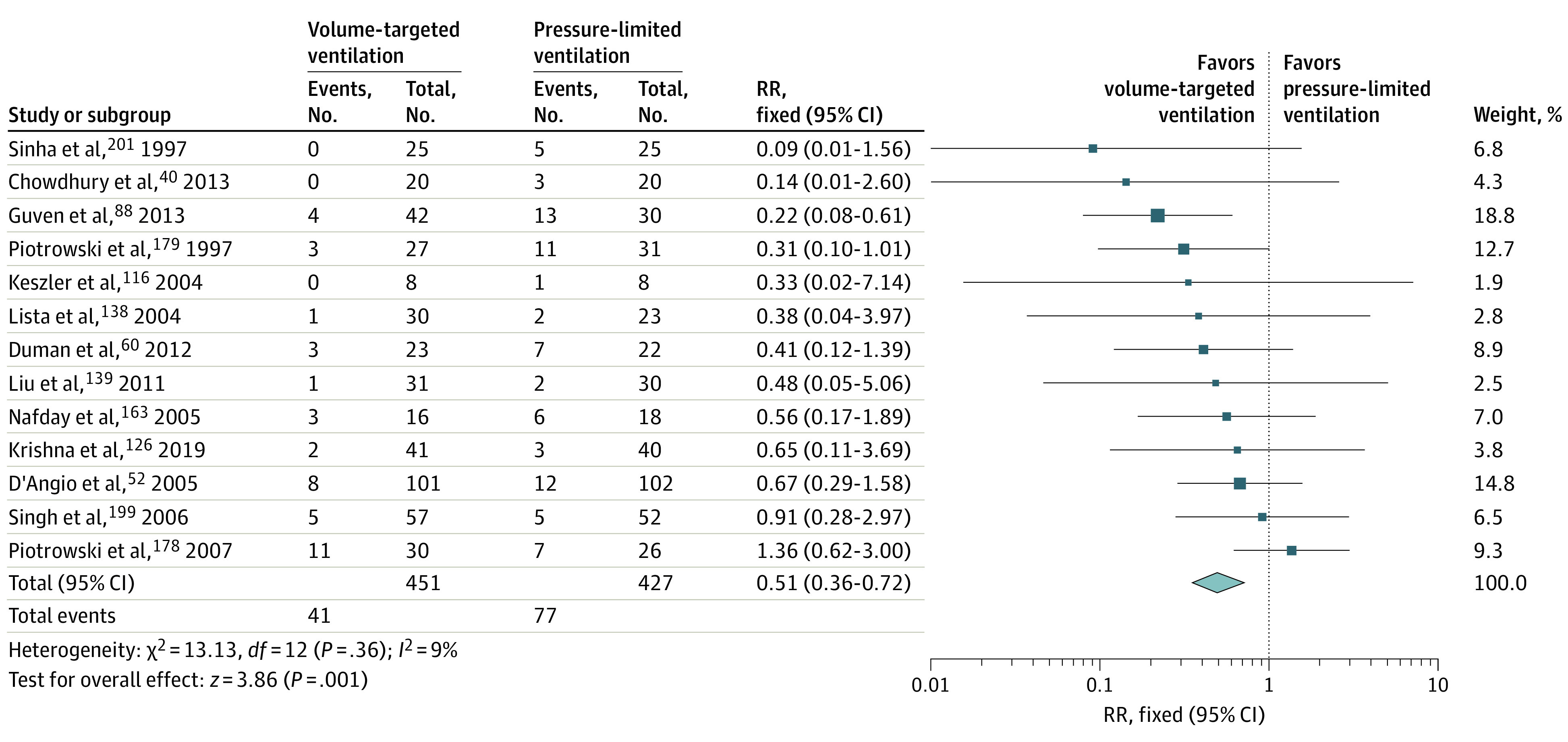
Forest Plot for Volume-Targeted vs Pressure-Limited Ventilation in Preterm Neonates for the Outcome of Severe Intraventricular Hemorrhage Diamond indicates the overall effect estimate from the meta-analysis, and squares indicate a point estimate for the individual study. RR indicates risk ratio.

Meta-analysis of data from 13 trials (878 participants)^40,52,60,88,116,126,138,139,163,178,179,199,201^ showed a large reduction in sIVH risk (RR, 0.51 [95% CI, 0.36-0.72]; *I*^2^ = 9%; ARD, −9% [95% CI, −13% to −5%]; NNT, 11 [95% CI, 7-23]) with volume-targeted ventilation vs pressure-limited ventilation (Table and Figure 3). We assessed the certainty of evidence as low, which was a downgrade for serious study design limitations and serious risk of publication bias (funnel plot: asymmetrical; Egger intercept test: 1-tailed *P* = .03) (eFigures 14 and 15 in Supplement 1).

Meta-analysis of data from 12 trials (5117 participants)^32,68,69,90,107,142,164,169,170,174,203,212^ showed a moderate reduction in sIVH risk (RR, 0.68 [95% CI, 0.57-0.83]; *I*^2^ = 45%; ARD, −3% [95% CI, −4% to −1%]; NNT, 34 [95% CI, 22-67]) with early erythropoiesis-stimulating agents (ESAs) vs placebo (Table; eFigure 16 in Supplement 1). We assessed the certainty of evidence as low, which was a downgrade for serious study design limitations and serious risk of heterogeneity. No statistically significant evidence of publication bias was found (funnel plot: symmetrical; Egger intercept test: 1-tailed *P* = .18) (eFigure 17 in Supplement 1).

Meta-analysis of data from 6 trials (1117 participants)^16,28,39,85,160,189^ showed a moderate reduction in sIVH risk (RR, 0.68 [95% CI, 0.48-0.97]; *I*^2^ = 2%; ARD, −4% [95% CI, −7% to 0%]; NNT, 26 [95% CI, 13-372]) with prophylactic ethamsylate administration vs placebo (Table; eFigure 18 in Supplement 1). We assessed the certainty of evidence as low, which was a downgrade for serious study design limitations and serious risk of imprecision. The other neonatal interventions that the meta-analysis showed as not having treatment effects are listed in the Table and eFigures 19 to 41 in Supplement 1.

### Outcome: cPVL

Meta-analysis of data from 3 trials (1551 participants)^68,164,203^ showed a moderate reduction in cPVL risk (RR, 0.59 [95% CI, 0.42-0.83]; *I*^2^ = 0%; ARD, −4% [95% CI, −7% to −1%]; NNT, 22 [95% CI, 14-57]) with early ESAs vs placebo (eTable 2 and eFigure 42 in Supplement 1). We assessed the certainty of evidence as low, which was a downgrade for serious study design limitations.

The data from 1 trial,^221^ including 64 participants, showed an increased risk of cPVL with elective high-frequency jet ventilation vs placebo (RR, 5.00; 95% CI, 1.19-21.04) (eTable 2 and eFigure 43 in Supplement 1). We assessed the certainty of evidence as low, which was a downgrade for serious study design limitation and serious risk of imprecision. The remaining interventions that the meta-analysis showed as not being associated with cPVL are provided in eTable 2 and eFigures 44 to 64 in Supplement 1.

### Outcome: Severe Brain Injury

Only a limited number of trials^27,43,45,46,61,93,105,120,121,165,180,190,191,192,208,213^ provided data on severe brain injury associated with various interventions (eTable 2 in Supplement 1). Meta-analysis of trials showed no significant differences in implications of various interventions, except for elective high-frequency oscillatory ventilation (HFOV) vs conventional ventilation. Specifically, the meta-analysis of data from 4 trials (1769 participants)^45,61,105,180^ showed a reduced risk of severe brain injury with elective HFOV vs placebo, and the evidence certainty was assessed as low (RR, 0.79 [95% CI, 0.63-0.99]; ARD, −4% [95% CI, −8% to −0%]; NNT, 23 [95% CI, 12-501]) (eTable 2 and eFigure 65 in Supplement 1). The interventions that the meta-analysis showed no treatment effect for severe brain injury are provided in eTable 2 and eFigures 66 to 71 in Supplement 1.

Subgroup and sensitivity analyses are provided in eTable 3 in Supplement 1. For most of the interventions, the analyses were not conducted due to a lack of subgroup data, high-quality studies, and the absence of substantial heterogeneity (*I*^2^ >50%).

## Discussion

In this systematic review and meta-analysis of 221 trials^9,10,11,12,13,14,15,16,17,18,19,20,21,22,23,24,25,26,27,28,29,30,31,32,33,34,35,36,37,38,39,40,41,42,43,44,45,46,47,48,49,50,51,52,53,54,55,56,57,58,59,60,61,62,63,64,65,66,67,68,69,70,71,72,73,74,75,76,77,78,79,80,81,82,83,84,85,86,87,88,89,90,91,92,93,94,95,96,97,98,99,100,101,102,103,104,105,106,107,108,109,110,111,112,113,114,115,116,117,118,119,120,121,122,123,124,125,126,127,128,129,130,131,132,133,134,135,136,137,138,139,140,141,142,143,144,145,146,147,148,149,150,151,152,153,154,155,156,157,158,159,160,161,162,163,164,165,166,167,168,169,170,171,172,173,174,175,176,177,178,179,180,181,182,183,184,185,186,187,188,189,190,191,192,193,194,195,196,197,198,199,200,201,202,203,204,205,206,207,208,209,210,211,212,213,214,215,216,217,218,219,220,221,222,223,224,225,226,227,228,229^ that assessed 44 perinatal interventions, we found with moderate certainty that antenatal corticosteroids for lung maturation (small decrease) and indomethacin prophylaxis (moderate decrease) were associated with reduced risk of sIVH in preterm neonates. We also found low certainty evidence that volume-targeted ventilation (large decrease), early ESAs (moderate decrease), and prophylactic ethamsylate (moderate decrease) were associated with lower risk of sIVH, whereas UCM (moderate increase) were associated with higher risk of sIVH in preterm neonates.

Severe IVH and cPVL have detrimental roles in childhood neurodevelopmental outcomes.^3^ Therefore, it is essential for clinicians to be aware of the evidence-based interventions available for reducing the risk of severe brain injury in preterm neonates. To our knowledge, this systematic review and meta-analysis was the first to collate and summarize the breadth of evidence for such potential interventions, albeit not all. By outlining the evidence and its certainty using the GRADE approach, we believe that this study helps clinicians and decision-makers to understand the role of these interventions in reducing the risk of sIVH and cPVL in preterm neonates.

**Figure 4.**
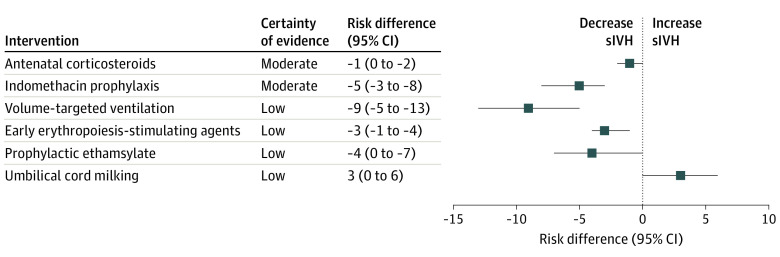
Summary Estimates of Meta-analyses of Clinical Trials Comparing Interventions for the Prevention of Severe Intraventricular Hemorrhage (sIVH) in Preterm Neonates Squares indicate the overall effect estimate from the meta-analysis for that intervention.

In this systematic review and meta-analysis, we found only a few perinatal interventions (antenatal corticosteroids,^66,75,76,89,133,157,158,175,197^ indomethacin prophylaxis,^23,24,46,57,92,108,127,147,148,149,150,159,192,224,228^ volume-targeted ventilation,^40,52,60,88,116,126,138,139,163,178,179,199,201^ early ESAs,^32,68,69,90,107,142,164,169,170,174,203,212^ and prophylactic ethamsylate^16,28,39,85,160,189^) that were associated with decreased risk of sIVH in preterm neonates (Figure 4). The certainty of the evidence for these interventions was low to moderate. We studied 6 antenatal interventions but found that only antenatal corticosteroid for lung maturity was beneficial in reducing sIVH risk in this preterm population. For this intervention, the certainty of the evidence was moderate and the effect size was small. Nonetheless, the small treatment effect of antenatal corticosteroids in sIVH is important. Additionally, several other benefits of antenatal corticosteroids, such as reduced perinatal and neonatal mortality, respiratory distress syndrome, need for mechanical ventilation, and necrotizing enterocolitis, compel their use in pregnant individuals who are at risk of preterm delivery.

We found no significant decrease in the risk of sIVH associated with the delivery room interventions assessed in this systematic review and meta-analysis. On the other hand, compared with DCC, UCM was associated with a moderate increase in the risk of sIVH in preterm neonates (low certainty). This finding is consistent with a finding from the PREMOD-2 (Premature Infants Receiving Cord Milking) trial,^112^ which was prematurely terminated due to a higher rate of sIVH occurring with UCM than with DCC in neonates born at less than 32 weeks’ gestation. In contrast, a recent network meta-analysis comparing DCC with UCM showed no difference in sIVH risk associated with UCM.^230^ It is important to note that the network meta-analysis used a random-effects model, which weighed the studies relatively more equally than would a fixed-effects meta-analysis in the presence of heterogeneity. On the other hand, we used a fixed-effects model, which offered more weight to the large trials (therefore, the analysis was affected by the largest-conducted PREMOD-2 trial^112^), and there was no significant between-studies heterogeneity. Nevertheless, the overall sample size from all of the included trials contributing to the meta-analysis was insufficient; hence, more data for this intervention are required.

Among the neonatal interventions, indomethacin prophylaxis was associated with a moderate decrease in sIVH risk in preterm neonates (moderate certainty). The use of indomethacin prophylaxis for reducing sIVH risk is a better option compared with its use for reducing the incidence of PDA and PDA requiring surgical ligation, which is now becoming obsolete given the uncertainties of the management of PDA. However, the lack of a long-term neurodevelopment benefit may prevent clinicians from using indomethacin prophylaxis for the short-term benefit. Nonetheless, clinicians should consider the baseline risks; given that the risk of sIVH for a particular newborn may be higher, prophylaxis may be beneficial for some preterm neonates.

The meta-analysis found a large reduction in sIVH risk associated with volume-targeted ventilation compared with pressure-limited ventilation for preterm neonates with respiratory distress syndrome; however, the evidence had low certainty. Nevertheless, volume-targeted ventilation offers several benefits, such as reduction in pneumothorax, chronic lung disease, hypocarbia, and ventilation duration, which may support its use in preterm infants.^231^ Despite these benefits, the uptake of volume-targeted ventilation has been slow, and efforts are ongoing to improve the understanding of volume-targeted ventilation and bridge the knowledge gap.^231^

The meta-analysis found a moderate reduction in sIVH and cPVL risk associated with early ESA (low certainty). However, the reduction in sIVH risk with early ESA was inconsistent across the studies, as evident by significant between-studies heterogeneity. Of the 3 large RCTs affecting the meta-analysis,^107,203,212^ 2 multicenter trials^203,212^ with substantial limitations in study design showed a large reduction in sIVH risk with erythropoietin. A recent multicenter RCT with no methodological concerns that included more fragile preterm infants (28 weeks’ vs <32 weeks’ gestation) and used a higher initial dose (1000 U/kg vs 500 U/kg) and prolonged duration (5-9 weeks vs 2 weeks) showed no reduction in sIVH risk with erythropoietin.^107^ Similarly, the reduction in cPVL risk was inconsistent, and the studies had substantial methodological limitations. We also found a moderate decrease in sIVH risk with prophylactic ethamsylate administration and in severe brain injury risk with elective HFOV; however, the confidence in the estimate was low, and the trials assessed in the meta-analysis were older and may not reflect current practice.

### Limitations

This systematic review and meta-analysis has several limitations. First, we did not study the treatment effects of the interventions for mortality, other short-term neonatal morbidities, and long-term neurodevelopmental outcomes, given that the study focused on comprehensively reviewing the interventions relevant to severe brain injury. Some of the interventions studied may not play a role in sIVH or severe brain injury but may be a considerable factor in other important outcomes; hence, to ensure the intervention is meaningful for a preterm newborn, clinicians should consider all of the critical outcomes in individual trials and their reviews. However, in the present study, we provided a balanced approach and conclusion for the potential interventions. Second, we evaluated interventions as decided a priori, which included an exhaustive list, but it may not be complete. Some interventions for preventing sIVH or severe brain injury, such as minimal handling of a preterm neonate, in utero transport for a premature neonate, hypothermia prevention, avoidance of boluses and bicarbonate therapy, and avoidance of fluctuation in carbon dioxide and blood pressure, have not been studied, as it may not be feasible to evaluate them in RCTs. Third, we did not examine the treatment effects of the interventions in milder forms of brain injury, such as any IVH or any PVL, which may still have considerable implications for long-term neurodevelopmental outcomes. Fourth, we had limited data for several intervention comparisons and sparse data for the cPVL outcome.

## Conclusions

In this systematic review and meta-analysis, a few interventions that were assessed in trials, including antenatal corticosteroids and indomethacin prophylaxis, were associated with reduction in sIVH risk with moderate certainty in preterm newborns; interventions such as volume-targeted ventilation, early ESAs, and prophylactic ethamsylate were associated with reduced risk with low certainty. However, clinicians should carefully consider all of the critical factors in such interventions, such as certainty of the evidence, effect size, clinical context, and methodological limitations and size of the studies included in the meta-analysis, before applying the interventions to clinical practice. This study offered a transparent and structured summary of the evidence using the GRADE approach, which may help readers understand the role of potential interventions in reducing the risk of severe brain injury in preterm neonates. Further studies are required to identify more interventions for reducing severe brain injury risk in preterm neonates. These studies should assess the implications of interventions for childhood neurodevelopmental outcomes.

## Notes

Supplement 1. **eAppendix 1.** Search Strategy **eAppendix 2.** Interventions Considered in the Systematic Review **eAppendix 3.** Screening Form **eAppendix 4.** Excluded Articles and Their Citations **eTable 1.** Characteristics of Studies Studying Interventions on Severe Brain Injury in Preterm Infants **eTable 2.** Summary of Meta-analysis of Interventions on Cystic Periventricular Leukomalacia (cPVL) and Severe Brain Injury **eTable 3.** Summary of Sensitivity and Subgroup Analysis of Interventions on Severe Intraventricular Hemorrhage (IVH) and cPVL **eFigure 1.** Study Flow Diagram Outlining Stages of Search Results and Filtering Process [as per Preferred Reporting Items for Systematic Reviews and Meta-Analyses (PRISMA) Guidelines] **eFigure 2.** Forest Plot for Comparison—Betamethasone for Lung Maturity vs. Dexamethasone for Lung Maturity for Outcome Severe IVH **eFigure 3.** Forest Plot for Comparison—Antenatal Steroid for Lung Maturity: Repeat Course vs. Single Course for Outcome Severe IVH **eFigure 4.** Forest Plot for Comparison—Antenatal MgSO4 Therapy for Neuroprotection or Tocolysis vs. Placebo for the Outcome Severe IVH **eFigure 5.** Forest Plot for Comparison—Antibiotics for PPROM vs. Placebo for the Outcome Severe IVH **eFigure 6.** Forest Plot for Umbilical Cord Milking vs. Delayed Cord Clamping in Preterm Infants for the Outcome Severe IVH **eFigure 7.** Forest Plot for Comparison—Lower FiO2 for Resuscitation vs. Higher FiO2 at Resuscitation for the Outcome Severe IVH **eFigure 8.** Forest Plot for Comparison—Sustained Inflation During Resuscitation vs. Standard Resuscitation for the Outcome Severe IVH **eFigure 9.** Forest Plot for Comparison—Delayed Cord Clamping vs. Early Cord Clamping for the Outcome Severe IVH **eFigure 10.** Forest Plot for Comparison—Umbilical Cord Milking vs. Early Cord Clamping for the Outcome Severe IVH **eFigure 11.** Forest Plot for Comparison—Delayed Cord Clamping With Respiratory Support vs. Delayed Cord Clamping Without Respiratory Support for the Outcome Severe IVH **eFigure 12.** Funnel Plot of Indomethacin Prophylaxis for Patent Ductus Arteriosus vs. Placebo for Outcome Severe IVH **eFigure 13.** Egger’s Regression Test for Assessing Publication Bias for Indomethacin Prophylaxis for Patent Ductus Arteriosus **eFigure 14.** Funnel Plot of Volume Guarantee vs. Pressure Limited Ventilation for Outcome Severe IVH **eFigure 15.** Egger’s Regression Test for Assessing Publication Bias for Volume Guarantee Ventilation vs. Pressure Limited Ventilation for Outcome Severe IVH **eFigure 16.** Forest Plot for Comparison—Early Erythropoiesis-Stimulating Agents vs. Placebo for Outcome Severe IVH **eFigure 17.** Funnel Plot of Early Erythropoiesis-Stimulating Agents vs. Placebo for Outcome Severe IVH **eFigure 18.** Forest Plot for Comparison—Prophylactic Ethamsylate Administration vs. Placebo for the Outcome Severe IVH **eFigure 19.** Forest Plot for Comparison—Supine Head Midline vs. Supine Head Rotated for the Outcome Severe IVH **eFigure 20.** Forest Plot for Comparison—LISA vs. INSURE Method for Surfactant Administration for the Outcome Severe IVH **eFigure 21.** Forest Plot for Comparison—Elective High-Frequency Oscillatory Ventilation (HFOV) vs. Conventional Ventilation Method for the Outcome Severe IVH **eFigure 22.** Forest Plot for Comparison—Elective High-Frequency Jet Ventilation (HFJV) vs. Conventional Ventilation Method for the Outcome Severe IVH **eFigure 23.** Forest Plot for Comparison—High Oxygen Saturation Target (91-95%) vs. Low Oxygen Saturation Target (85-89%) in NICU for the Outcome Severe IVH **eFigure 24.** Forest Plot for Comparison—Permissive Hypercapnia vs. Normocapnia During Ventilation for the Outcome Severe IVH **eFigure 25.** Forest Plot for Comparison—Early Extubation vs. Delayed Extubation During Ventilation for the Outcome Severe IVH **eFigure 26.** Forest Plot for Comparison—Caffeine Prophylaxis or Treatment for Apnea or Post-Extubation vs. Placebo for the Outcome Severe IVH **eFigure 27.** Forest Plot for Comparison—High Dose Caffeine Prophylaxis for Apnea or Post-Extubation vs. Low Dose Caffeine for the Outcome Severe IVH **eFigure 28.** Forest Plot for Comparison—Sedation During Ventilation: Midazolam vs. Placebo for the Outcome Severe IVH **eFigure 29.** Forest Plot for Comparison— Sedation During Ventilation: Opioid vs. Placebo for the Outcome Severe IVH **eFigure 30.** Forest Plot for Comparison—Neuromuscular Paralysis During Ventilation vs. Placebo for the Outcome Severe IVH **eFigure 31.** Forest Plot for Comparison—Volume Expansion vs. Inotropes for Hypotension for the Outcome Severe IVH **eFigure 32.** Forest Plot for Comparison—Dopamine vs. Dobutamine for Hypotension for the Outcome Severe IVH **eFigure 33.** Forest Plot for Comparison—Ibuprofen Prophylaxis for PDA vs. Placebo for the Outcome Severe IVH **eFigure 34.** Forest Plot for Comparison—Indomethacin Pre-Symptomatic Treatment for PDA vs. Placebo for the Outcome Severe IVH **eFigure 35.** Forest Plot for Comparison—Ibuprofen Pre-Symptomatic Treatment for PDA vs. Placebo for the Outcome Severe IVH **eFigure 36.** Forest Plot for Comparison—Restrictive vs. Liberal Packed Red Cell Transfusion for Anemia for the Outcome Severe IVH **eFigure 37.** Forest Plot for Comparison—Prophylactic Factor VII Administration vs. Placebo for the Outcome Severe IVH **eFigure 38.** Forest Plot for Comparison—Prophylactic Antithrombin III Administration vs. Placebo for the Outcome Severe IVH **eFigure 39.** Forest Plot for Comparison—Prophylactic Heparin Administration vs. Placebo for the Outcome Severe IVH **eFigure 40.** Forest Plot for Comparison—Vitamin A Supplementation vs. Placebo for the Outcome Severe IVH **eFigure 41.** Forest Plot for Comparison—Vitamin E Supplementation vs. Placebo for the Outcome Severe IVH **eFigure 42.** Forest Plot for Comparison—Early Erythropoiesis-Stimulating Agents vs. Placebo for Outcome cPVL **eFigure 43.** Forest Plot for Comparison—Elective HFJV vs. Conventional Ventilation Method for the Outcome cPVL **eFigure 44.** Forest Plot for Comparison—Any Steroids for Lung Maturity vs. Placebo for the Outcome cPVL **eFigure 45.** Forest Plots for Comparison—Betamethasone for Lung Maturity vs. Betamethasone for Lung Maturity for Outcome cPVL **eFigure 46.** Forest Plot for Comparison—Antenatal Steroid for Lung Maturity: Repeat Course vs. Single Course for Outcome cPVL **eFigure 47.** Forest Plot for Comparison—Antenatal MgSO4 Therapy for Neuroprotection vs. Placebo for the Outcome cPVL **eFigure 48.** Forest Plot for Comparison—Lower FiO2 for Resuscitation vs. Higher FiO2 for the Outcome cPVL **eFigure 49.** Forest Plot for Comparison—Sustained Inflation During Resuscitation vs. Standard Resuscitation for the Outcome cPVL **eFigure 50.** Forest Plot for Comparison—Umbilical Cord Milking vs. Early Cord Clamping for the Outcome cPVL **eFigure 51.** Forest Plot for Comparison—Supine Head Midline vs. Supine Head Rotated for the Outcome cPVL **eFigure 52.** Forest Plot for Comparison—Less Invasive Surfactant Administration (LISA) vs. Intubate, Surfactant, Extubate (INSURE) Method for Surfactant Administration for the Outcome Severe cPVL **eFigure 53.** Forest Plot for Comparison—Volume Target Ventilation vs. Pressure Limited Ventilation for the Outcome Severe cPVL **eFigure 54.** Forest Plot for Comparison—Elective HFOV vs. Conventional Ventilation Method for the Outcome cPVL **eFigure 55.** Forest Plot for Comparison—High Oxygen Saturation Target (91-95%) vs. Low Oxygen Saturation Target (85-89%) in NICU for the Outcome cPVL **eFigure 56.** Forest Plot for Comparison—Permissive Hypercapnia vs. Normocapnia During Ventilation for the Outcome cPVL **eFigure 57.** Forest Plot for Comparison—Caffeine Prophylaxis or Treatment for Apnea or Post-Extubation vs. Placebo for the Outcome cPVL **eFigure 58.** Forest Plot for Comparison—High Dose Caffeine Prophylaxis for Apnea or Post-Extubation vs. Low Dose Caffeine for the Outcome cPVL **eFigure 59.** Forest Plot for Comparison—Sedation During Ventilation: Opioid vs. Placebo for the Outcome cPVL **eFigure 60.** Forest Plot for Comparison—Neuromuscular Paralysis During Ventilation vs. Placebo for the Outcome cPVL **eFigure 61.** Forest Plot for Comparison—Indomethacin Prophylaxis for PDA vs. Placebo for the Outcome cPVL **eFigure 62.** Forest Plot for Comparison—Indomethacin Pre-Symptomatic Treatment for PDA vs. Placebo for the Outcome cPVL **eFigure 63.** Forest Plot for Comparison—Ibuprofen Prophylaxis for PDA vs. Placebo for the Outcome cPVL **eFigure 64.** Forest Plot for Comparison—Restrictive vs. Liberal Packed Red Cell Transfusion for Anemia for the Outcome cPVL **eFigure 65.** Forest Plot for Comparison—Elective HFOV vs. Conventional Ventilation Method for the Outcome Severe Brain Injury **eFigure 66.** Forest Plot for Comparison—Any Steroids for Lung Maturity vs. Placebo for the Outcome Severe Brain Injury **eFigure 67.** Forest Plot for Comparison—Delayed Cord Clamping vs. Early Cord Clamping for the Outcome Severe Brain Injury **eFigure 68.** Forest Plot for Comparison—High vs. Low Oxygen Saturation Targets for the Outcome Severe Brain Injury **eFigure 69.** Forest Plot for Comparison—Caffeine Prophylaxis or Treatment for Apnea or Post-Extubation for the Outcome Severe Brain Injury **eFigure 70.** Forest Plot for Comparison—Indomethacin Prophylaxis for the Outcome Severe Brain Injury **eFigure 71.** Forest Plot for Comparison—Restrictive vs. Liberal Packed Red Cell Transfusion for Anemia for the Outcome Severe Brain Injury

Supplement 2.
Data Sharing Statement
