# Supplementary material for: Interventions to Reduce Severe Brain Injury Risk in Preterm Neonates: A Systematic Review and Meta-analysis
Source: JAMA Netw Open. 2023 Apr 13;6(4):e237473. doi: 10.1001/jamanetworkopen.2023.7473 (PMC10102877; doi:10.1001/jamanetworkopen.2023.7473)
Supplement: Supplement 1. — eAppendix 1. Search Strategy eAppendix 2. Interventions Considered in the Systematic Review eAppendix 3. Screening Form eAppendix 4. Excluded Articles and Their Citations eTable 1. Characteristics of Studies Studying Interventions on Severe Brain Injury in Preterm Infants eTable 2. Summary of Meta-analysis of Interventions on Cystic Periventricular Leukomalacia (cPVL) and Severe Brain Injury eTable 3. Summary of Sensitivity and Subgroup Analysis of Interventions on Severe Intraventricular Hemorrhage (IVH) and cPVL eFigure 1. Study Flow Diagram Outlining Stages of Search Results and Filtering Process [as per Preferred Reporting Items for Systematic Reviews and Meta-Analyses (PRISMA) Guidelines] eFigure 2. Forest Plot for Comparison—Betamethasone for Lung Maturity vs. Dexamethasone for Lung Maturity for Outcome Severe IVH eFigure 3. Forest Plot for Comparison—Antenatal Steroid for Lung Maturity: Repeat Course vs. Single Course for Outcome Severe IVH eFigure 4. Forest Plot for Comparison—Antenatal MgSO4 Therapy for Neuroprotection or Tocolysis vs. Placebo for the Outcome Severe IVH eFigure 5. Forest Plot for Comparison—Antibiotics for PPROM vs. Placebo for the Outcome Severe IVH eFigure 6. Forest Plot for Umbilical Cord Milking vs. Delayed Cord Clamping in Preterm Infants for the Outcome Severe IVH eFigure 7. Forest Plot for Comparison—Lower FiO2 for Resuscitation vs. Higher FiO2 at Resuscitation for the Outcome Severe IVH eFigure 8. Forest Plot for Comparison—Sustained Inflation During Resuscitation vs. Standard Resuscitation for the Outcome Severe IVH eFigure 9. Forest Plot for Comparison—Delayed Cord Clamping vs. Early Cord Clamping for the Outcome Severe IVH eFigure 10. Forest Plot for Comparison—Umbilical Cord Milking vs. Early Cord Clamping for the Outcome Severe IVH eFigure 11. Forest Plot for Comparison—Delayed Cord Clamping With Respiratory Support vs. Delayed Cord Clamping Without Respiratory Support for the Outcome Severe IVH eFigure 12. Funnel Plot of Indom [file jamanetwopen-e237473-s001.pdf]

Supplemental Online Content

Razak A, Patel W, Durrani NUR, Pullattayil AK. Interventions to reduce severe brain injury risk in preterm neonates: a systematic review and meta-analysis. *JAMA Netw Open*. 2023;6(4):e237473. doi:10.1001/jamanetworkopen.2023.7473

eAppendix 1. Search Strategy

eAppendix 2. Interventions Considered in the Systematic Review

eAppendix 3. Screening Form

eAppendix 4. Excluded Articles and Their Citations

eTable 1. Characteristics of Studies Studying Interventions on Severe Brain Injury in Preterm Infants

eTable 2. Summary of Meta-analysis of Interventions on Cystic Periventricular Leukomalacia (cPVL) and Severe Brain Injury

eTable 3. Summary of Sensitivity and Subgroup Analysis of Interventions on Severe Intraventricular Hemorrhage (IVH) and cPVL

eFigure 1. Study Flow Diagram Outlining Stages of Search Results and Filtering Process [as per Preferred Reporting Items for Systematic Reviews and Meta-Analyses (PRISMA) Guidelines]

eFigure 2. Forest Plot for Comparison—Betamethasone for Lung Maturity vs. Dexamethasone for Lung Maturity for Outcome Severe IVH

eFigure 3. Forest Plot for Comparison—Antenatal Steroid for Lung Maturity: Repeat Course vs. Single Course for Outcome Severe IVH

eFigure 4. Forest Plot for Comparison—Antenatal MgSO4 Therapy for Neuroprotection or Tocolysis vs. Placebo for the Outcome Severe IVH

eFigure 5. Forest Plot for Comparison—Antibiotics for PPROM vs. Placebo for the Outcome Severe IVH

eFigure 6. Forest Plot for Umbilical Cord Milking vs. Delayed Cord Clamping in Preterm Infants for the Outcome Severe IVH

eFigure 7. Forest Plot for Comparison—Lower FiO2 for Resuscitation vs. Higher FiO2 at Resuscitation for the Outcome Severe IVH

eFigure 8. Forest Plot for Comparison—Sustained Inflation During Resuscitation vs. Standard Resuscitation for the Outcome Severe IVH

eFigure 9. Forest Plot for Comparison—Delayed Cord Clamping vs. Early Cord Clamping for the Outcome Severe IVH

eFigure 10. Forest Plot for Comparison—Umbilical Cord Milking vs. Early Cord Clamping for the Outcome Severe IVH

© 2023 Razak A et al. *JAMA Network Open.*

eFigure 11. Forest Plot for Comparison—Delayed Cord Clamping With Respiratory Support vs. Delayed Cord Clamping Without Respiratory Support for the Outcome Severe IVH

eFigure 12. Funnel Plot of Indomethacin Prophylaxis for Patent Ductus Arteriosus vs. Placebo for Outcome Severe IVH

eFigure 13. Egger's Regression Test for Assessing Publication Bias for Indomethacin Prophylaxis for Patent Ductus Arteriosus

eFigure 14. Funnel Plot of Volume Guarantee vs. Pressure Limited Ventilation for Outcome Severe IVH

eFigure 15. Egger's Regression Test for Assessing Publication Bias for Volume Guarantee Ventilation vs. Pressure Limited Ventilation for Outcome Severe IVH

eFigure 16. Forest Plot for Comparison—Early Erythropoiesis-Stimulating Agents vs. Placebo for Outcome Severe IVH

eFigure 17. Funnel Plot of Early Erythropoiesis-Stimulating Agents vs. Placebo for Outcome Severe IVH

eFigure 18. Forest Plot for Comparison—Prophylactic Ethamsylate Administration vs. Placebo for the Outcome Severe IVH

eFigure 19. Forest Plot for Comparison—Supine Head Midline vs. Supine Head Rotated for the Outcome Severe IVH

eFigure 20. Forest Plot for Comparison—LISA vs. INSURE Method for Surfactant Administration for the Outcome Severe IVH

eFigure 21. Forest Plot for Comparison—Elective High-Frequency Oscillatory Ventilation (HFOV) vs. Conventional Ventilation Method for the Outcome Severe IVH

eFigure 22. Forest Plot for Comparison—Elective High-Frequency Jet Ventilation (HFJV) vs. Conventional Ventilation Method for the Outcome Severe IVH

eFigure 23. Forest Plot for Comparison—High Oxygen Saturation Target (91-95%) vs. Low Oxygen Saturation Target (85-89%) in NICU for the Outcome Severe IVH

eFigure 24. Forest Plot for Comparison—Permissive Hypercapnia vs. Normocapnia During Ventilation for the Outcome Severe IVH

eFigure 25. Forest Plot for Comparison—Early Extubation vs. Delayed Extubation During Ventilation for the Outcome Severe IVH

eFigure 26. Forest Plot for Comparison—Caffeine Prophylaxis or Treatment for Apnea or Post-Extubation vs. Placebo for the Outcome Severe IVH

eFigure 27. Forest Plot for Comparison—High Dose Caffeine Prophylaxis for Apnea or Post-Extubation vs. Low Dose Caffeine for the Outcome Severe IVH

eFigure 28. Forest Plot for Comparison—Sedation During Ventilation: Midazolam vs. Placebo for the Outcome Severe IVH

© 2023 Razak A et al. *JAMA Network Open.*

eFigure 29. Forest Plot for Comparison— Sedation During Ventilation: Opioid vs. Placebo for the Outcome Severe IVH

eFigure 30. Forest Plot for Comparison—Neuromuscular Paralysis During Ventilation vs. Placebo for the Outcome Severe IVH

eFigure 31. Forest Plot for Comparison—Volume Expansion vs. Inotropes for Hypotension for the Outcome Severe IVH

eFigure 32. Forest Plot for Comparison—Dopamine vs. Dobutamine for Hypotension for the Outcome Severe IVH

eFigure 33. Forest Plot for Comparison—Ibuprofen Prophylaxis for PDA vs. Placebo for the Outcome Severe IVH

eFigure 34. Forest Plot for Comparison—Indomethacin Pre-Symptomatic Treatment for PDA vs. Placebo for the Outcome Severe IVH

eFigure 35. Forest Plot for Comparison—Ibuprofen Pre-Symptomatic Treatment for PDA vs. Placebo for the Outcome Severe IVH

eFigure 36. Forest Plot for Comparison—Restrictive vs. Liberal Packed Red Cell Transfusion for Anemia for the Outcome Severe IVH

eFigure 37. Forest Plot for Comparison—Prophylactic Factor VII Administration vs. Placebo for the Outcome Severe IVH

eFigure 38. Forest Plot for Comparison—Prophylactic Antithrombin III Administration vs. Placebo for the Outcome Severe IVH

eFigure 39. Forest Plot for Comparison—Prophylactic Heparin Administration vs. Placebo for the Outcome Severe IVH

eFigure 40. Forest Plot for Comparison—Vitamin A Supplementation vs. Placebo for the Outcome Severe IVH

eFigure 41. Forest Plot for Comparison—Vitamin E Supplementation vs. Placebo for the Outcome Severe IVH

eFigure 42. Forest Plot for Comparison—Early Erythropoiesis-Stimulating Agents vs. Placebo for Outcome cPVL

eFigure 43. Forest Plot for Comparison—Elective HFJV vs. Conventional Ventilation Method for the Outcome cPVL

eFigure 44. Forest Plot for Comparison—Any Steroids for Lung Maturity vs. Placebo for the Outcome cPVL

eFigure 45. Forest Plots for Comparison—Betamethasone for Lung Maturity vs. Betamethasone for Lung Maturity for Outcome cPVL

eFigure 46. Forest Plot for Comparison—Antenatal Steroid for Lung Maturity: Repeat Course vs. Single Course for Outcome cPVL

© 2023 Razak A et al. *JAMA Network Open.*

eFigure 47. Forest Plot for Comparison—Antenatal MgSO4 Therapy for Neuroprotection vs. Placebo for the Outcome cPVL

eFigure 48. Forest Plot for Comparison—Lower FiO2 for Resuscitation vs. Higher FiO2 for the Outcome cPVL

eFigure 49. Forest Plot for Comparison—Sustained Inflation During Resuscitation vs. Standard Resuscitation for the Outcome cPVL

eFigure 50. Forest Plot for Comparison—Umbilical Cord Milking vs. Early Cord Clamping for the Outcome cPVL

eFigure 51. Forest Plot for Comparison—Supine Head Midline vs. Supine Head Rotated for the Outcome cPVL

eFigure 52. Forest Plot for Comparison—Less Invasive Surfactant Administration (LISA) vs. Intubate, Surfactant, Extubate (INSURE) Method for Surfactant Administration for the Outcome Severe cPVL

eFigure 53. Forest Plot for Comparison—Volume Target Ventilation vs. Pressure Limited Ventilation for the Outcome Severe cPVL

eFigure 54. Forest Plot for Comparison—Elective HFOV vs. Conventional Ventilation Method for the Outcome cPVL

eFigure 55. Forest Plot for Comparison—High Oxygen Saturation Target (91-95%) vs. Low Oxygen Saturation Target (85-89%) in NICU for the Outcome cPVL

eFigure 56. Forest Plot for Comparison—Permissive Hypercapnia vs. Normocapnia During Ventilation for the Outcome cPVL

eFigure 57. Forest Plot for Comparison—Caffeine Prophylaxis or Treatment for Apnea or Post-Extubation vs. Placebo for the Outcome cPVL

eFigure 58. Forest Plot for Comparison—High Dose Caffeine Prophylaxis for Apnea or Post-Extubation vs. Low Dose Caffeine for the Outcome cPVL

eFigure 59. Forest Plot for Comparison—Sedation During Ventilation: Opioid vs. Placebo for the Outcome cPVL

eFigure 60. Forest Plot for Comparison—Neuromuscular Paralysis During Ventilation vs. Placebo for the Outcome cPVL

eFigure 61. Forest Plot for Comparison—Indomethacin Prophylaxis for PDA vs. Placebo for the Outcome cPVL

eFigure 62. Forest Plot for Comparison—Indomethacin Pre-Symptomatic Treatment for PDA vs. Placebo for the Outcome cPVL

eFigure 63. Forest Plot for Comparison—Ibuprofen Prophylaxis for PDA vs. Placebo for the Outcome cPVL

© 2023 Razak A et al. *JAMA Network Open.*

eFigure 64. Forest Plot for Comparison—Restrictive vs. Liberal Packed Red Cell Transfusion for Anemia for the Outcome cPVL

eFigure 65. Forest Plot for Comparison—Elective HFOV vs. Conventional Ventilation Method for the Outcome Severe Brain Injury

eFigure 66. Forest Plot for Comparison—Any Steroids for Lung Maturity vs. Placebo for the Outcome Severe Brain Injury

eFigure 67. Forest Plot for Comparison—Delayed Cord Clamping vs. Early Cord Clamping for the Outcome Severe Brain Injury

eFigure 68. Forest Plot for Comparison—High vs. Low Oxygen Saturation Targets for the Outcome Severe Brain Injury

eFigure 69. Forest Plot for Comparison—Caffeine Prophylaxis or Treatment for Apnea or Post-Extubation for the Outcome Severe Brain Injury

eFigure 70. Forest Plot for Comparison—Indomethacin Prophylaxis for the Outcome Severe Brain Injury

eFigure 71. Forest Plot for Comparison—Restrictive vs. Liberal Packed Red Cell Transfusion for Anemia for the Outcome Severe Brain Injury

This supplemental material has been provided by the authors to give readers additional information about their work.

© 2023 Razak A et al. *JAMA Network Open.*

eAppendix 1. Search Strategy

Database(s): Ovid MEDLINE(R) ALL: 1946 to September 08, 2022
First Search was conducted on 16th May 2020; the search has been updated on 22nd October 2021 and 8th September 2022

#	Searches	Results
1	exp Infant, Newborn/ or neonat*.mp.	804206
2	exp Infant/ or infan*.mp.	1389301
3	newborn.mp. or exp Infant, Newborn/	811062
4	exp Infant, Premature/ or preterm.mp.	119056
5	premature.mp. or exp Infant, Premature/ or exp Infant, Extremely Premature/	214615
6	premie.mp.	29
7	low birth weight.mp. or exp Infant, Low Birth Weight/	52655
8	very low birth weight.mp. or exp Infant, Very Low Birth Weight/	15158
9	extremely low birth weight.mp. or exp Infant, Extremely Low Birth Weight/	3745
10	or/1-9	1712280
11	randomized.mp. or exp Randomized Controlled Trial/	979027
12	exp Clinical Trial/ or trial.mp.	1347829
13	randomly.mp.	391699
14	controlled clinical trial.mp. or exp Controlled Clinical Trial/	673723
15	or/11-14	1832476
16	exp Cerebral Hemorrhage/ or intraventricular hemorrhage.mp.	40707
17	intraventricular hemorrhages.mp.	258
18	intra-ventricular hemorrhage.mp.	76
19	intra-ventricular hemorrhages.mp.	5
20	periventricular-intraventricular hemorrhage.mp.	166
21	periventricular-intraventricular hemorrhages.mp.	6
22	PIVH.mp.	137
23	peri-intraventricular hemorrhage.mp.	75
24	peri-intraventricular hemorrhages.mp.	13
25	periventricular leukomalacia.mp. or exp Leukomalacia, Periventricular/	2889
26	intracranial hemorrhage.mp. or exp Intracranial Hemorrhages/	84717
27	intracranial hemorrhages.mp. or exp Intracranial Hemorrhages/	78756
28	germinal matrix hemorrhage.mp.	251
29	germinal matrix hemorrhages.mp.	27
30	brain bleed.mp.	25
31	brain injury.mp. or exp Brain Injuries/	112952
32	brain injuries.mp.	69672
33	PVL.mp.	4541
34	or/16-33	199972

© 2023 Razak A et al. *JAMA Network Open.*

35	10 and 15 and 34	2169
36	exp animals/ not humans/	5043654
37	35 not 36	1951
38	limit 37 to dt=20200516-20211231	165
39	limit 37 to dt=20211231-20220910	58

Database(s): Embase Classic+Embase-1947 to 2022 September 08
First Search was conducted on 16th May 2020; the search has been updated on 22nd October 2021 and 8th September 2022.

#	Searches	Results
1	exp Infant, Newborn/ or neonat*.mp.	860637
2	exp Infant/ or infan*.mp.	1534244
3	newborn.mp. or exp Infant, Newborn/	797532
4	exp Infant, Premature/ or preterm.mp.	192597
5	premature.mp. or exp Infant, Premature/ or exp Infant, Extremely Premature/	329858
6	premie.mp.	60
7	low birth weight.mp. or exp Infant, Low Birth Weight/	80809
8	very low birth weight.mp. or exp Infant, Very Low Birth Weight/	19697
9	extremely low birth weight.mp. or exp Infant, Extremely Low Birth Weight/	5327
10	or/1-9	1896070
11	randomized.mp. or exp Randomized Controlled Trial/	1326912
12	exp Clinical Trial/ or trial.mp.	2617159
13	randomly.mp.	521230
14	controlled clinical trial.mp. or exp Controlled Clinical Trial/	940146
15	or/11-14	3122705
16	exp Cerebral Hemorrhage/ or intraventricular hemorrhage.mp.	173566
17	intraventricular hemorrhages.mp.	363
18	intra-ventricular hemorrhage.mp.	169
19	intra-ventricular hemorrhages.mp.	4
20	periventricular-intraventricular hemorrhage.mp.	195
21	periventricular-intraventricular hemorrhages.mp.	7
22	PIVH.mp.	153
23	peri-intraventricular hemorrhage.mp.	97
24	peri-intraventricular hemorrhages.mp.	14
25	periventricular leukomalacia.mp. or exp Leukomalacia, Periventricular/	7599
26	intracranial hemorrhage.mp. or exp Intracranial Hemorrhages/	175092
27	intracranial hemorrhages.mp. or exp Intracranial Hemorrhages/	172676
28	germinal matrix hemorrhage.mp.	379
29	germinal matrix hemorrhages.mp.	40
30	brain bleed.mp.	33

© 2023 Razak A et al. *JAMA Network Open.*

31	brain injury.mp. or exp Brain Injuries/	236548
32	brain injuries.mp.	13352
33	PVL.mp.	7028
34	or/16-33	406687
35	10 and 15 and 34	5489
36	exp animals/ not humans/	12275913
37	35 not 36	3476
38	limit 37 to dc=20200516-20211231	481
39	limit 37 to dc=20211231-20220831	298

Database CENTRAL, Search Name: brain injury
Firs Search was conducted on 16th May 2020; the search has been updated on 22nd October 2021 and 10th September (Trials other than Medline and Embase only downloaded)

ID	Search	Hits
#1	infant (Word variations have been searched)	68573
#2	preterm (Word variations have been searched)	16348
#3	premature (Word variations have been searched)	27641
#4	infan*	72420
#5	Newborn (Word variations have been searched)	31725
#6	premie (Word variations have been searched)	147
#7	low birth weight (Word variations have been searched)	9704
#8	MeSH descriptor: [Infant, Low Birth Weight] explode all trees	2332
#9	very low birth weight (Word variations have been searched)	5090
#10	MeSH descriptor: [Infant, Very Low Birth Weight] explode all trees	1038
#11	extremely low birth weight (Word variations have been searched)	1867
#12	MeSH descriptor: [Infant, Extremely Low Birth Weight] explode all trees	131
#13	{OR #1-#12}	91237
#14	intraventricular hemorrhage (Word variations have been searched)	2146
#15	"Intraventricular hemorrhage"	1923
#16	MeSH descriptor: [Infant, Newborn, Diseases] explode all trees	7713
#17	intraventricular NEAR2 hemorrhage (Word variations have been searched)	39215
#18	Cerebral NEAR2 Hemorrhage (Word variations have been searched)	64213
#19	MeSH descriptor: [Cerebral Hemorrhage] explode all trees	1113
#20	MeSH descriptor: [Cerebral Intraventricular Hemorrhage] explode all trees	22
#21	intra-ventricular hemorrhage (Word variations have been searched)	70
#22	periventricular-intraventricular hemorrhage (Word variations have been searched)	42
#23	PIVH (Word variations have been searched)	18
#24	peri-intraventricular hemorrhage (Word variations have been searched)	14
#25	periventricular NEAR2 leukomalacia	1184

© 2023 Razak A et al. *JAMA Network Open.*

#26	MeSH descriptor: [Leukomalacia, Periventricular] explode all trees	62
#27	intracranial hemorrhage (Word variations have been searched)	4093
#28	germinal matrix hemorrhage (Word variations have been searched)	41
#29	brain NEAR2 bleed (Word variations have been searched)	125581
#30	brain NEAR2 injury (Word variations have been searched)	136624
#31	PVL (Word variations have been searched)	419
#32	{OR #14-#31}	170705
#33	#13 AND #32	2197
#34	#13 AND #32 with Publication Year from 2020 to 2021, with Cochrane Library publication date Between May 2022 and Oct 2021, in Trials	427
#35	#13 AND #32 with Publication Year from 2021 to 2022, with Cochrane Library publication date Between Oct 2021 and Sep 2022, in Trials	240

Database – CINAHL
Firs Search was conducted on 16th May2020 (n=606); the search has been updated on 22nd October 2021 (n=40) and 10th September 2022 (n=31).

#	**Query**	**Results**
S24	S5 AND S9 AND S23	677
S23	S10 OR S11 OR S12 OR S13 OR S14 OR S15 OR S16 OR S17 OR S18 OR S19 OR S20 OR S21 OR S22	53,072
S22	PVL	723
S21	brain N2 injury OR brain N2 injuries	37,672
S20	brain N2 bleed OR brain N2 bleeds	26
S19	("germinal matrix" N2 (hemorrhage OR heamorrhages)) OR (germinal matrix N2 (hemorrhages OR heamorrhages))	102
S18	(intracranial N2 hemorrhage OR intracranial N2 haemorrhage) OR (intracranial N2 hemorrhages OR intracranial N2 haemorrhages)	6,438
S17	periventricular N2 leukomalacia OR MJ periventricular leukomalacia	656
S16	"peri-intraventricular" N2 hemorrhage OR "peri-intraventricular" N2 hemorrhages	28
S15	PIVH	19
S14	"periventricular-intraventricular" N2 hemorrhage OR "periventricular-intraventricular" N2 hemorrhages OR MJ periventricular-intraventricular hemorrhage	49
S13	intraventricular N2 hemorrhages OR intraventricular N2 hemorrhage OR intra-ventricular N2 hemorrhage OR intra-ventricular N2 hemorrhages	2,468
S12	intraventricular N2 hemorrhages	2,443
S11	intraventricular hemorrhages	6,967
S10	Cerebral N2 Hemorrhage OR intraventricular N2 hemorrhage	10,119
S9	(S6 OR S7 OR S8)	509,385
S8	(randomized controlled trials or rtc or randomised control trials or randomized clinical trial or randomized controlled study) OR MJ Controlled Clinical Trial	229,347
S7	Controlled Clinical Trial OR MJ Controlled Clinical Trial	37,162
S6	random*	492,224

© 2023 Razak A et al. *JAMA Network Open.*

S5	(S1 OR S2 OR S3 OR S4)	349,626
S4	premie	13,060
S3	low birth weight OR MJ very low birthweight OR extremely low birth weight	17,489
S2	(premature infants or preterm infants OR preterm) OR MJ (premature infants or preterm infants OR preterm)	51,931
S1	(infants or newborn or neonate) OR MH (infants or newborns or neonates)	338,921

© 2023 Razak A et al. *JAMA Network Open.*

eAppendix 2. Interventions Considered in the Systematic Review

ANTENATAL INTERVENTIONS:

1. Antenatal corticosteroids for lung maturity
2. Betamethasone vs. Dexamethasone for lung maturity
3. Repeat antenatal steroids vs. single course antenatal steroids
4. Magnesium sulfate for neuroprotection or tocolysis
5. Antibiotics for premature rupture of membranes
6. Cesarean section vs. vaginal delivery for preterm birth

DELIVERY ROOM INTERVENTIONS:

1. Lower vs. higher FiO2 for resuscitation
2. Sustained inflation vs. standard resuscitation
3. Delayed cord clamping vs. early cord clamping
4. Umbilical cord milking vs. early cord clamping
5. Umbilical cord milking vs. delayed cord clamping
6. Delayed cord clamping with respiratory support vs. delayed cord clamping without respiratory support

POSTNATAL INTERVENTIONS:

1. Supine head midline vs. Supine head rotated
2. LISA vs. INSURE
3. Volume guarantee vs. pressure limited ventilation
4. Elective HFOV vs. conventional ventilation
5. Elective HFJV vs. conventional ventilation
6. Oxygen saturation target after birth: 85-89% vs. 91-95%
7. Permissive hypercapnia vs. normocapnia
8. Early extubation vs. delayed extubation
9. Caffeine prophylaxis or treatment for apnea or post-extubation
10. High dose vs. low dose caffeine for apnea or post-extubation
11. Sedation during ventilation: midazolam vs. placebo
12. Sedation during ventilation: opioids vs. placebo
13. Sedation during ventilation: phenobarbitone vs. placebo
14. Neuromuscular paralysis during ventilation vs placebo
15. Early erythropoiesis-stimulating agents vs placebo
16. Volume expansion vs. inotropes (any) for hypotension
17. Dopamine vs. dobutamine for hypotension
18. Indomethacin prophylaxis for PDA vs placebo
19. Indomethacin pre-symptomatic treatment for PDA vs placebo
20. Ibuprofen prophylaxis for PDA vs placebo
21. Ibuprofen pre-symptomatic treatment for PDA vs placebo

© 2023 Razak A et al. *JAMA Network Open.*

22. Restrictive vs. liberal packed red cell transfusion for anemia
23. Low vs. high threshold for platelet transfusion for thrombocytopenia
24. Prophylactic plasma administration vs placebo to reduce IVH/PVL
25. Prophylactic factor VII administration vs placebo to reduce IVH/PVL
26. Prophylactic antithrombin III administration vs placebo to reduce IVH/PVL
27. Prophylactic ethamsylate administration vs placebo to reduce IVH/PVL
28. Prophylactic heparin administration vs placebo to reduce IVH/PVL
29. Stem cell therapy
30. Vitamin A supplementation vs. placebo for the outcome severe IVH
31. Vitamin E supplementation vs. placebo for the outcome severe IVH
32. Vitamin K supplementation vs. placebo for the outcome severe IVH

© 2023 Razak A et al. *JAMA Network Open.*

eAppendix 3. Screening Form

INCLUSION: STUDY MUST MEET ALL FOUR STEPS TO BE INCLUDED

STEP 1: We will only include randomised or quasi-randomised clinical trials reporting at least one or more outcomes (sIVH, cPVL, and severe brain injury) identified in the systematic review

STEP 2: Preterm infants <37 weeks gestational age.

STEP 3: Must include at least one of the 44 interventions as listed above (antenatal (n=6), delivery room (n=6), and neonatal (n=32)).

STEP 4: Must report at least one of the outcomes of our interest:

- sIVH: defined as hemorrhage into the ventricles with ventricular distension, intra-parenchymal hemorrhage, or parenchymal hemorrhagic infarct (grade III or IV IVH Papile grading), identified any time before discharge on cranial ultrasound.
- cPVL: defined as white matter injury characterized by the necrosis of white matter near the lateral ventricles (must include cystic changes), identified any time before discharge on cranial ultrasound.
- Severe brain injury: defined as the presence of either sIVH or cPVL

EXCLUSION: STUDY WILL BE EXCLUDED IF IT MEETS ANY ONE OF THE FOLLOWING:

- Non-randomised studies
- Studies including term infants
- Studies evaluating interventions that are not mentioned above.

© 2023 Razak A et al. *JAMA Network Open.*

eAppendix 4. Excluded Articles and Their Citations

**Study author, year**	**Reasons for exclusion**
Abbas, 2019[1]	Review article
Adamska, 2015[2]	No outcome of interest.
Aher, 2006[3]	Cochrane Review
Ahn, 2018[4]	Not an RCT.
Altaany, 2015[5]	Retrospective study design, not an RCT.
Ambalavanan 2015[6]	Follow-up study, not eligible.
Amin 2007[7]	Metanalysis (Review article)
Amorin 1999[8]	Studied effect of antenatal corticosteroid in pregnant women, not eligible.
Ankola 1993[9]	Not mentioned Severe IVH of cPVL, no outcome of interest.
Araki 2018[10]	Systematic Review, not eligible
Ardell 2018[11]	Intervention was not considered in our study.
Askie 2917[12]	Cochrane Review
Abdel-Rehman[13]	Not an RCT.
Adcock 2018[14]	Retrospective study design
Ahmed 2019[15]	Intervention not considered in our study
Airoldi 2009[16]	Case-control study, not eligible.
Bassler 2006[17]	Cochrane Review
Been 2011[18]	Meta-analysis-Review article
Bellu 2008[19]	Cochrane Review
Bhuta 1997[20]	Study already considered, duplicate study
Blankenship 2019[21]	Not an RCT.
Boedy 2005[22]	No outcome of interest.
Bourchier 1997[23]	Intervention was not considered in our study.
Bowes 2004[24]	Full text not found.
Bradshaw 2015 [25]	Study protocol.
Brion 2003[26]	Cochrane Review
Brooks 1999[27]	No outcome of interest.
Bruschettini 2016[28]	Duplicate, the published study included
Cao 2020[29]	Full text not found, although it looks like a review article.
Corbet 1995[30]	A pilot study, study protocol.
Carlo 1987[31]	Not mentioned severe IVH of cPVL, no outcome of interest.
Carroll 2019[32]	Retrospective cohort study, not an RCT.
Chapman 2016[33]	Systematic review
Anand 1999[34]	Duplicate data, published in two different journals, one already included

© 2023 Razak A et al. *JAMA Network Open.*

Chatzakis 2020[1]	No outcome of interest.
Chiswick 1991[35]	IVH is not classified into I to IV grade, not eligible
Clark 1996[36]	Meta-analysis-Review article
Clyman 2006[37]	Follow-up study.
Cooke 2003[38]	Cochrane Review
Cools 2015[39]	Review article
Crane 2003[40]	Poster, no eligible.
Crowther 2012[41]	Duplicate data, published in two different journals. One already included.
Dagenais 2017[42]	No outcome of interest.
Dang 2015[43]	No outcome of interest.
Darlow 2016[44]	Review article
Dilmen 2014[45]	No outcome of interest.
Dolfin 1994[46]	Intervention was not considered in our study.
Dogoff 2017[47]	Study protocol, No outcome of interest.
Egarter 1996[48]	No outcome of interest.
Elbourne 2001[49]	Follow-up study at 2 years of age
Elimian 2013[50]	Duplicate data, published in two different journals. One already included.
Fergusson 2012[51]	Intervention not considered in our study
Fogarty 2018[52]	Not an RCT.
Fowlie 2003[53]	Review article.
Foligno 2020[54]	Studied bovine vs. porcine surfactant, not considered in our study.
Fujiwara 1990[55]	Included preterm with grade 2 IVH, met exclusion criteria.
Gerstmann 1996[56]	No outcome of interest.
Gork 2008[57]	Review Article.
Grevsen 2020[58]	Not an RCT.
Haidenn 2005[58]	Studied EPO in anemia, not considered as an intervention in our study.
Hammerman 2008[59]	Study protocol, poster.
Harding 2001[60]	Follow-up study
Henderson 2010[61]	Studied Caffeine vs. Theophylline in AOP, not a comparison in our review.
Herrera 2007[62]	Cochrane Review
Hodnett 1999[63]	No outcome of interest.
Horton 2015[64]	Secondary follow-up study.
How 1998[65]	Intervention was not considered in our study.
Hutzal 2008[66]	Review article.
Ibrahim 2000[67]	No outcome of interest.
Ibrahim 2014[68]	Review article

© 2023 Razak A et al. *JAMA Network Open.*

Jacqz-Aigrain1994[69]	Did not report IVH.
Jarde 2020[70]	Not an RCT.
Jiang 2012[71]	Did not report IVH.
Joshi 2006[72]	Cochrane Review
Juul 2008[73]	Study protocol, phase 1/II trial, we included the final article.
Kalani 2016[74]	Compared Indomethacin vs. Ibuprofen, we did not consider this intervention.
Kashanian 2018[75]	Studied different Dexamethasone regimes, intervention not considered.
Keszler 1991[76]	Duplicate data, we already considered one latest study.
Khuwuthyakorn 2018[77]	Evaluated oral Indomethacin vs. oral Ibuprofen; we did not consider the intervention.
Klauser 2012[78]	No outcome of interest.
Kribs 2015[79]	No outcome of interest.
Kua 2017[80]	Not an RCT.
Lago 1998[81]	No separate data on severe IVH, cystic PVL or severe brain injury provided
Lietich 1998[82]	Meta-analysis, review article.
Leuchter 2014[83]	Did not mention IVH, PVL mentioned but no data on cPVL
Li 2020[84]	No outcome of interest.
Liao 2014[85]	Not an RCT.
Liu 2016[86]	Chinese article, Full text not available.
Laughon 2009[87]	Study protocol, pilot study.
Long 1991[88]	Intervention was not considered in our study.
Maruyama 2012[89]	No outcome of interest.
Mirzarahimi 2018[90]	Compared efficacy of Curosurf vs. Survanta, innervation was not considered.
Mittendorf 2002[91]	IVH is not graded into I to IV and PVL is not defined.
Malhotra 2017[92]	A prospective observational study, not an RCT.
Ment 1996[93]	Duplicate data, we already considered one study.
Mercer 2016[94]	Follow-up study
Mirzamoradi 2014[95]	IVH and PVL are not defined, no outcome of interest.
Mitra 2016[96]	Network meta-analysis, excluded.
Nagano 2018[97]	Review article.
Nayeri 2014[98]	IVH and PVL are not clearly defined.
Neary 2016[99]	Cochrane Review.
Nguyen 2013[100]	Study includes term neonates, excluded.
Nicolades 2016[101]	Poster presentation. No outcome of interest.
Nijman 2018[102]	Secondary analysis excluded.
O'Sullivan 2019[103]	Secondary analysis of prospective gathered data.
Oberthuer 2017[104]	Conference abstract

© 2023 Razak A et al. *JAMA Network Open.*

Oei 2018[105]	Review article.
Ohlsson 2017[106]	Cochrane review.
Osborn 2003[107]	IVH and PVL are not defined clearly.
Ovalle 2006[108]	Only includes Antibiotics for Preterm labor, no PPROM, so excluded.
Park 2017[109]	Review article about Mesenchymal stem cell transplantation, not an RCT.
Pasquier 2019[110]	IVH not graded, no outcome of interest.
Peng 2014[111]	A meta-analysis, not an RCT.
Perlman 1985[112]	No outcome of interest.
Qublan 2001[113]	Prospective study but no randomisation.
Quinn 1993[114]	No outcome of interest.
Rennie 1986[115]	IVH and PVL are not defined clearly.
Reynolds 1985[116]	No outcome of interest.
Rezaie 2016[117]	Compared single vs. double Betamethasone dose, it's a study protocol and did not have any outcome of our interest.
Roberts 2017[118]	Cochrane Review.
Rojas 2009[119]	Studied the effect of Surfactant with or without ventilation, we did not consider this intervention.
Romero 1993[120]	Antibiotics for preterm labor with intact membranes, no PPROM, so excluded.
Rook 2012[121]	Study protocol.
Ruegger 2015[122]	Included all grades of IVH, so excluded.
Saugstad 2017[123]	Conference abstract.
Scheller 1994[124]	Commentary and recommendation, not an RCT.
Schmidt 2001[125]	No outcome of interest.
Schmolzer 2018[126]	Feasibility trial.
Schulte 2005[127]	Long-term follow-up study.
Shah 2008[128]	Review article.
Shankaran 1995[129]	No outcome of interest.
Sharafutdinova 2019[130]	A prospective observational study, not an RCT.
Shirk 2019[131]	Conference abstract.
Silver 1996[132]	Studied the effect of Surfactant on IVH, we did not consider this intervention.
Simons 2006[133]	Did not specify severe IVH or cPVL.
Sinha 2005[134]	No outcome of interest.
Sluncheva 2006[135]	Studied prevention of IVH and surfactant, not an inclusion criterion.
Skoll 2018[136]	Review article.
Smit 2013[137]	Studied postnatal phenobarbital on neonatal outcome, we did not include this intervention.
Smith 2007[138]	No outcome of interest.
Sosenko 2012[139]	Studied symptomatic PDA; we only considered prophylactic/asymptomatic PDA

© 2023 Razak A et al. *JAMA Network Open.*

Sommers 2012[140]	Prospective study, not an RCT.
Stock 2012[141]	Review article.
Strickland 2007[142]	Response letter, commentary to an original article.
Su 2003[143]	Comparison between Indomethacin and Ibuprofen for treatment of PDA, not an inclusion criterion for our study.
Subhedar 2003[144]	Cochrane Review.
Supapannachart 1999[145]	The article did not clearly define severe IVH, cPVL, or severe brain injury, so we excluded the article.
Thomas 2005[146]	Review article.
Thome 2000[147]	No outcome of interest.
Thome 2018[148]	Long-term follow-up study.
Unal 2017[149]	No outcome of interest.
Van Teeffelen 2014[150]	No outcome of interest.
Van Overmeire 2001[151]	Included symptomatic PDA cases; we only considered prophylactic and pre-symptomatic cases.
Veldman 2006[152]	Study protocol.
Walti 1973[153]	Prospective study, not an RCT.
Wang 2015[154]	A meta-analysis, Review article.
Wardle 2001[155]	Not defined severe IVH, no outcome of interest.
Wenstrom 2007[156]	No outcome of interest.
Wheeler 2011[157]	Review article.
Whyte 2011[158]	Cochrane review.
Thorp 1995[159]	No outcome of interest.
Weesner 1987[160]	Did not mention separately grade III-IV IVH or no mention of cPVL
Wyllie 2018[161]	Commentary to an original article. Not an RCT.
Xu 2016[162]	Studied Atosiban in threatened preterm labor; cases did not have PPROM
Yadav 2014[163]	Compared oral Indomethacin vs. oral Ibuprofen in the closure of PDA, intervention was not considered in our study.
Yang 2018[164]	Did not mention separately grade III-IV IVH or cPVL
Zeng 2019[165]	Review article.
Sunil 2022[166]	No IVH reported
McDonald 2022[167]	Studied different doses of Betamethasone, not considered as an intervention
Benavides 2021[168]	Follow up study of Bell 2005
Bravo 2021[169]	Considered symptomatic PDA neonates, excluded.
Erdeve 2021[170]	Not an RCT
Mari 2022 [171]	Review article
Li 2021[172]	No mention of severe IVH or cPVL.
Sabzehai 2022[173]	No grading of IVH

© 2023 Razak A et al. *JAMA Network Open.*

Iranpour 2022[174]	No outcome of interest.

Reference list for excluded studies:

1. Abbas S, Keir AK. In preterm infants, does fluid restriction, as opposed to liberal fluid prescription, reduce the risk of important morbidities and mortality? Journal of Paediatrics & Child Health 2019;55(7):860-866
2. Adamska E, Helwich E, Rutkowska M, Zacharska E, Piotrowska A. [Comparison of the efficacy of ibuprofen and indomethacin in the treatment of patent ductus arteriosus in prematurely born infants]. Porownanie ibuprofenu i indometacyny w leczeniu przetrwalego przewodu tetniczego u noworodkow urodzonych przedwczesnie 2005;9(3 Pt 1):335-354
3. Aher S, Ohlsson A. Late erythropoietin for preventing red blood cell transfusion in preterm and/or low birth weight infants. The Cochrane database of systematic reviews 2006(3):CD004868
4. Ahn SY, Chang YS, Sung SI, Park WS. Mesenchymal Stem Cells for Severe Intraventricular Hemorrhage in Preterm Infants: Phase I Dose-Escalation Clinical Trial. Stem cells translational medicine 2018;7(12):847-856
5. Altaany D, Natarajan G, Gupta D, Zidan M, Chawla S. Severe Intraventricular Hemorrhage in Extremely Premature Infants: Are high Carbon Dioxide Pressure or Fluctuations the Culprit? American Journal of Perinatology 2015;32(9):839-844
6. Ambalavanan N, Carlo WA, Wrage LA, et al. PaCO2 in surfactant, positive pressure, and oxygenation randomised trial (SUPPORT). Archives of disease in childhood Fetal and neonatal edition 2015;100(2):F145-149
7. Amin SB, Sinkin RA, Glantz JC. Metaanalysis of the effect of antenatal indomethacin on neonatal outcomes. American Journal of Obstetrics and Gynecology 2007;197(5):486.e481-486.e410
8. Amorim MM, Santos LC, Faundes A. Corticosteroid therapy for prevention of respiratory distress syndrome in severe preeclampsia. American journal of obstetrics and gynecology 1999;180(5):1283-1288
9. Ankola PA, Atakent YS. Effect of adding heparin in very low concentration to the infusate to prolong the patency of umbilical artery catheters. American journal of perinatology 1993;10(3):229-232

© 2023 Razak A et al. *JAMA Network Open.*

10. Araki S, Kato S, Namba F, Ota E. Vitamin A to prevent bronchopulmonary dysplasia in extremely low birth weight infants: a systematic review and meta-analysis. PloS one 2018;13(11):e0207730
11. Ardell S, Offringa M, Ovelman C, Soll R. Prophylactic vitamin K for the prevention of vitamin K deficiency bleeding in preterm neonates. Cochrane Database of Systematic Reviews 2018(2)
12. Askie LM, Darlow BA, Davis PG, et al. Effects of targeting lower versus higher arterial oxygen saturations on death or disability in preterm infants. Cochrane Database Syst Rev 2017;4(1469-493X (Electronic)):CD011190
13. Abdel-Rahman AM, Rosenberg AA. Prevention of intraventricular hemorrhage in the premature infant. Clinics in perinatology 1994;21(3):505-521
14. Adcock B, Hanna M, Giannone P, Bauer J, Carpenter S. Renal consequences of low-dose indomethacin for prevention of intraventricular hemmorhage in extremely premature infants. Journal of Investigative Medicine 2018;66(2):510
15. Ahmed MS, Giesinger RE, Ibrahim M, et al. Clinical and echocardiography predictors of response to inhaled nitric oxide in hypoxic preterm neonates. Journal of Paediatrics and Child Health 2019;55(7):753-761
16. Airoldi MJ, Silva SBdC, de Souza RCT. Neurological evaluation of neonates with intraventricular hemorrhage and periventricular leukomalacia. Revista Neurociencias 2009;17(1):24-29
17. Bassler D, Millar D, Schmidt B. Antithrombin for respiratory distress syndrome in preterm infants. The Cochrane database of systematic reviews 2006(4):CD005383
18. Been JV, Degraeuwe PL, Kramer BW, Zimmermann LJI. Antenatal steroids and neonatal outcome after chorioamnionitis: A meta-analysis. BJOG: An International Journal of Obstetrics and Gynaecology 2011;118(2):113-122
19. Bellù R, de Waal KA, Zanini R. Opioids for neonates receiving mechanical ventilation. Cochrane Database of Systematic Reviews 2008(1)
20. Bhuta T, Henderson-Smart DJ. Elective high-frequency oscillatory ventilation versus conventional ventilation in preterm infants with pulmonary dysfunction: systematic review and meta-analyses. Pediatrics 1997;100(5):E6

© 2023 Razak A et al. *JAMA Network Open.*

21. Blankenship SA, Brown KE, Stout MJ, Tuuli MG. 483: Antenatal corticosteroids in preterm growth-restricted fetuses: A systematic review and meta- analysis. American Journal of Obstetrics and Gynecology 2019;220(1):S323-S324
22. Boedy RF, Mathew OP, Bell EF, Strauss RG, Widness JA. Randomized trial of liberal versus restrictive guidelines for red blood cell transfusion in preterm infants...Bell EF, Strauss RG, Widness JA et al. Randomized trial of liberal versus restrictive guidelines for red blood cell transfusion in preterm infants. Pediatrics. 2005;115:1685-1691. Pediatrics 2005;116(4):1048-1050
23. Bourchier D, Weston PJ, Bourchier D, Weston PJ. Randomised trial of dopamine compared with hydrocortisone for the treatment of hypotensive very low birthweight infants. Archives of Disease in Childhood -- Fetal & Neonatal Edition 1997;76(3):F174-178
24. Bowes WA, Jr. [Commentary on] Effect of magnesium sulfate given for neuroprotection before preterm birth: a randomized, controlled trial. Obstetrical & Gynecological Survey 2004;59(5):330-332
25. Bradshaw LE, Pushpa-Rajah A, Dorling J, Mitchell EJ, Duley L. Cord pilot trial: update to randomised trial protocol. Trials 2015;16(1):1-3
26. Brion LP, Bell EF, Raghuveer TS. Vitamin E supplementation for prevention of morbidity and mortality in preterm infants. The Cochrane database of systematic reviews 2003(4):CD003665
27. Brooks SE, Marcus DM, Gillis D, et al. The effect of blood transfusion protocol on retinopathy of prematurity: A prospective, randomized study. Pediatrics 1999;104(3 Pt 1):514-518
28. Bruschettini M, Romantsik O, Zappettini S, et al. Heparin for the prevention of intraventricular haemorrhage in preterm infants. The Cochrane database of systematic reviews 2016(5):CD011718
29. Cao ZL, Pan JJ, Shen X, et al. Less invasive surfactant administration in preterm infants with respiratory distress syndrome-an updated meta-analysis. Journal of the Chinese Medical Association 2020;83(2):170-179
30. Corbet A, Gerdes J, Long W, et al. Double-blind, randomized trial of one versus three prophylactic doses of synthetic surfactant in 826 neonates weighing 700 to

© 2023 Razak A et al. *JAMA Network Open.*

1100 grams: effects on mortality rate. American Exosurf Neonatal Study Groups I and IIa. The Journal of pediatrics 1995;126(6):969-978
31. Carlo WA, Chatburn RL, Martin RJ. Randomized trial of high-frequency jet ventilation versus conventional ventilation in respiratory distress syndrome. The Journal of pediatrics 1987;110(2):275-282
32. Carroll K, Kumar P, Prazad P, et al. Midline head positioning for prevention of intraventricular hemorrhage (IVH) in infants born at 23-28 weeks gestation: A multicenter retrospective cohort stud. Pediatrics 2019;144(2)
33. Chapman J, Marfurt S, Reid J. Effectiveness of Delayed Cord Clamping in Reducing Postdelivery Complications in Preterm Infants: A Systematic Review. The Journal of perinatal & neonatal nursing 2016;30(4):372-378
34. Anand KJ, Barton BA, McIntosh N, et al. Analgesia and sedation in preterm neonates who require ventilatory support: results from the NOPAIN trial. Neonatal Outcome and Prolonged Analgesia in Neonates. Archives of pediatrics & adolescent medicine 1999;153(4):331-338
35. Chiswick M, Gladman G, Sinha S, Toner N, Davies J. Vitamin E supplementation and periventricular hemorrhage in the newborn. The American journal of clinical nutrition 1991;53(1 Suppl):370S-372S
36. Clark RH, Dykes FD, Bachman TE, Ashurst JT. Intraventricular hemorrhage and high-frequency ventilation: a meta-analysis of prospective clinical trials. Pediatrics 1996;98(6 Pt 1):1058-1061
37. Clyman RI, Saha S, Jobe A, Oh W. Indomethacin prophylaxis for preterm infants: the impact of 2 multicentered randomized controlled trials on clinical practice. The Journal of pediatrics 2007;150(1):46-50.e42
38. Cooke L, Steer PA, Woodgate PG. Indomethacin for asymptomatic patent ductus arteriosus in preterm infants. Cochrane Database of Systematic Reviews 2003:N.PAG-N.PAG
39. Cools F, Offringa M, Askie LM. Elective high frequency oscillatory ventilation versus conventional ventilation for acute pulmonary dysfunction in preterm infants. The Cochrane database of systematic reviews 2015(3):CD000104

© 2023 Razak A et al. *JAMA Network Open.*

40. Crane J, Armson A, Brunner M, et al. Antenatal corticosteroid therapy for fetal maturation. Journal of obstetrics and gynaecology Canada : JOGC = Journal d'obstetrique et gynecologie du Canada : JOGC 2003;25(1):45-52
41. Crowther CA, Aghajafari F, Askie LM, et al. Repeat prenatal corticosteroid prior to preterm birth: a systematic review and individual participant data meta-analysis for the PRECISE study group (prenatal repeat corticosteroid international IPD study group: assessing the effects using the best level of evidence) - study protocol. Systematic reviews 2012;1:12
42. Dagenais C, Lewis-Mikhael A-M, Grabovac M, Mukerji A, McDonald SD. What is the safest mode of delivery for extremely preterm cephalic/non-cephalic twin pairs? A systematic review and meta-analyses. BMC pregnancy and childbirth 2017;17(1):397
43. Dang D, Zhang C, Shi S, et al. Umbilical cord milking reduces need for red cell transfusions and improves neonatal adaptation in preterm infants: Meta-analysis. The journal of obstetrics and gynaecology research 2015;41(6):890-895
44. Darlow BA, Graham PJ, Rojas-Reyes MX. Vitamin A supplementation to prevent mortality and short- and long-term morbidity in very low birth weight infants. Cochrane Database of Systematic Reviews 2016(8)
45. Dilmen U, Ozdemir R, Tatar Aksoy H, et al. Early regular versus late selective poractant treatment in preterm infants born between 25 and 30 gestational weeks: a prospective randomized multicenter study. The journal of maternal-fetal & neonatal medicine : the official journal of the European Association of Perinatal Medicine, the Federation of Asia and Oceania Perinatal Societies, the International Society of Perinatal Obstetricians 2014;27(4):411-415
46. Dolfin T, Zamir C, Regev R, Ben Ari J, Wolach B. Effect of surfactant replacement therapy on the outcome of premature infants with respiratory distress syndrome. Israel journal of medical sciences 1994;30(4):267-270
47. Dugoff L, Berghella V, Mackeen AD, Goetzl L, Ludmir J. Prevention of preterm birth with pessary in singletons (PoPPS): A randomized controlled trial. American Journal of Obstetrics and Gynecology 2017;216(1):S4-S5

© 2023 Razak A et al. *JAMA Network Open.*

48. Egarter C, Leitich H, Karas H, et al. Antibiotic treatment in preterm premature rupture of membranes and neonatal morbidity: a metaanalysis. American journal of obstetrics and gynecology 1996;174(2):589-597
49. Elbourne D, Ayers S, Dellagrammaticas H, et al. Randomised controlled trial of prophylactic etamsylate: follow up at 2 years of age. Archives of disease in childhood Fetal and neonatal edition 2001;84(3):F183-187
50. Elimian A, Goodman J, Escobedo M, et al. A randomized controlled trial of immediate versus delayed cord clamping in the preterm neonate. American Journal of Obstetrics and Gynecology 2013;208(1):S22
51. Fergusson DA, Hebert P, Hogan DL, et al. Effect of fresh red blood cell transfusions on clinical outcomes in premature, very low-birth-weight infants: the ARIPI randomized trial. JAMA 2012;308(14):1443-1451
52. Fogarty M, Osborn DA, Askie L, et al. Delayed vs early umbilical cord clamping for preterm infants: a systematic review and meta-analysis. American journal of obstetrics and gynecology 2018;218(1):1-18
53. Fowlie PW, Davis PG. Prophylactic indomethacin for preterm infants: a systematic review and meta-analysis. Archives of disease in childhood Fetal and neonatal edition 2003;88(6):F464-466
54. Foligno S, Luca DD, De Luca D. Porcine versus bovine surfactant therapy for RDS in preterm neonates: pragmatic meta-analysis and review of physiopathological plausibility of the effects on extra-pulmonary outcomes. Respiratory Research 2020;21(1):1-10
55. Fujiwara T, Konishi M, Chida S, et al. Surfactant replacement therapy with a single postventilatory dose of a reconstituted bovine surfactant in preterm neonates with respiratory distress syndrome: Final analysis of a multicenter, double-blind, randomized trial and comparison with similar trials. Pediatrics 1990;86(5):753-764
56. Gerstmann DR, Minton SD, Stoddard RA, et al. The Provo multicenter early high-frequency oscillatory ventilation trial: improved pulmonary and clinical outcome in respiratory distress syndrome. Pediatrics 1996;98(6 Pt 1):1044-1057

© 2023 Razak A et al. *JAMA Network Open.*

57. Gork AS, Ehrenkranz RA, Bracken MB. Continuous infusion versus intermittent bolus doses of indomethacin for patent ductus arteriosus closure in symptomatic preterm infants. The Cochrane database of systematic reviews 2008(1):CD006071
58. Haiden N, Cardona F, Schwindt J, et al. Changes in thrombopoiesis and platelet reactivity in extremely low birth weight infants undergoing erythropoietin therapy for treatment of anaemia of prematurity. Thromb Haemost 2005;93(1):118-123
59. Hammerman C, Shchors I, Jacobson S, et al. Ibuprofen versus continuous indomethacin in premature neonates with patent ductus arteriosus: is the difference in the mode of administration? Pediatric research 2008;64(3):291-297
60. Harding JE, Pang J, Knight DB, Liggins GC. Do antenatal corticosteroids help in the setting of preterm rupture of membranes? American journal of obstetrics and gynecology 2001;184(2):131-139
61. Henderson-Smart DJ, Steer PA. Caffeine versus theophylline for apnea in preterm infants. Cochrane Database Syst Rev 2010(1):CD000273
62. Herrera C, Holberton J, Davis P. Prolonged versus short course of indomethacin for the treatment of patent ductus arteriosus in preterm infants. The Cochrane database of systematic reviews 2007(2):CD003480
63. Hodnett E. Antenatal oral dexamethasone for neonatal respiratory distress syndrome increased neonatal morbidity [commentary on Egerman RS, Mercer BM, Doss JL, et al. A randomized, controlled trial of oral and intramuscular dexamethasone in the prevention of neonatal respiratory distress syndrome. AM J OBSTET GYNECOL 1998 Nov;179:1120-3]. Evidence Based Nursing 1999:73-73
64. Horton AL, Lai Y, Rouse DJ, et al. Effect of magnesium sulfate administration for neuroprotection on latency in women with preterm premature rupture of membranes. American journal of perinatology 2015;32(4):387-392
65. How HY, Cook CR, Cook VD, Miles DE, Spinnato JA. Preterm premature rupture of membranes: aggressive tocolysis versus expectant management. The Journal of maternal-fetal medicine 1998;7(1):8-12
66. Hutzal CE, Boyle EM, Kenyon SL, et al. Use of antibiotics for the treatment of preterm parturition and prevention of neonatal morbidity: a metaanalysis. American journal of obstetrics and gynecology 2008;199(6):620.e621-628

© 2023 Razak A et al. *JAMA Network Open.*

67. Ibrahim HM, Krouskop RW, Lewis DF, Dhanireddy R. Placental transfusion: umbilical cord clamping and preterm infants. Journal of perinatology : official journal of the California Perinatal Association 2000;20(6):351-354
68. Ibrahim M, Ho SKY, Yeo CL. Restrictive versus liberal red blood cell transfusion thresholds in very low birth weight infants: a systematic review and meta-analysis. Journal of paediatrics and child health 2014;50(2):122-130
69. Jacqz-Aigrain E, Daoud P, Burtin P, Desplanques L, Beaufils F. Placebo-controlled trial of midazolam sedation in mechanically ventilated newborn babies. Lancet 1994;344(8923):646-650
70. Jarde A, Feng YY, Viaje KA, Shah PS, McDonald SD. Vaginal birth vs caesarean section for extremely preterm vertex infants: a systematic review and meta-analyses. Archives of Gynecology and Obstetrics 2020;301(2):447-458
71. Jiang H-h, Cheng R, Kan Q, et al. [Clinical evaluation of the effects of morphine in mechanical ventilation of neonates]. Zhonghua er ke za zhi = Chinese journal of pediatrics 2012;50(5):350-355
72. Joshi VH, Bhuta T. Rescue high frequency jet ventilation versus conventional ventilation for severe pulmonary dysfunction in preterm infants. The Cochrane database of systematic reviews 2006(1):CD000437
73. Juul SE, McPherson RJ, Bauer LA, et al. A phase I/II trial of high-dose erythropoietin in extremely low birth weight infants: pharmacokinetics and safety. Pediatrics 2008;122(2):383-391
74. Kalani M, Shariat M, Khalesi N, Farahani Z, Ahmadi S. A Comparison of Early Ibuprofen and Indomethacin Administration to Prevent Intraventricular Hemorrhage Among Preterm Infants. Acta medica Iranica 2016;54(12):788-792
75. Kashanian M, Eshraghi N, Sheikhansari N, Bordbar A, Khatami E. Comparison between two doses of betamethasone administration with 12 hours vs. 24 hours intervals on prevention of respiratory distress syndrome: a randomised trial. Journal of Obstetrics and Gynaecology 2018;38(6):770-776
76. Keszler M, Donn SM, Bucciarelli RL, et al. Multicenter controlled trial comparing high-frequency jet ventilation and conventional mechanical ventilation in

© 2023 Razak A et al. *JAMA Network Open.*

newborn infants with pulmonary interstitial emphysema. The Journal of pediatrics 1991;119(1 Pt 1):85-93
77. Khuwuthyakorn V, Jatuwattana C, Silvilairat S, Tantiprapha W. Oral indomethacin versus oral ibuprofen for treatment of patent ductus arteriosus: a randomised controlled study in very low-birthweight infants. Paediatrics and international child health 2018;38(3):187-192
78. Klauser CK, Briery CM, Keiser SD, et al. Effect of antenatal tocolysis on neonatal outcomes. The journal of maternal-fetal & neonatal medicine : the official journal of the European Association of Perinatal Medicine, the Federation of Asia and Oceania Perinatal Societies, the International Society of Perinatal Obstetricians 2012;25(12):2778-2781
79. Kribs A, Roll C, Gopel W, et al. Nonintubated Surfactant Application vs Conventional Therapy in Extremely Preterm Infants: A Randomized Clinical Trial. JAMA pediatrics 2015;169(8):723-730
80. Kua KP, Lee SWH. Systematic review and meta-analysis of clinical outcomes of early caffeine therapy in preterm neonates. British journal of clinical pharmacology 2017;83(1):180-191
81. Lago P, Benini F, Agosto C, Zacchello F. Randomised controlled trial of low dose fentanyl infusion in preterm infants with hyaline membrane disease. Arch Dis Child Fetal Neonatal Ed 1998;79(3):F194-197
82. Leitich H, Egarter C, Reisenberger K, Kaider A, Berghammer P. Concomitant use of glucocorticoids: a comparison of two metaanalyses on antibiotic treatment in preterm premature rupture of membranes. American journal of obstetrics and gynecology 1998;178(5):899-908
83. Leuchter RH-V, Gui L, Poncet A, et al. Association between early administration of high-dose erythropoietin in preterm infants and brain MRI abnormality at term-equivalent age. JAMA 2014;312(8):817-824
84. Li J, Yu B, Wang W, et al. Does intact umbilical cord milking increase infection rates in preterm infants with premature prolonged rupture of membranes? Journal of Maternal-Fetal & Neonatal Medicine 2020;33(2):184-190

© 2023 Razak A et al. *JAMA Network Open.*

85. Liao SMC, Rao R, Mathur AM. Head position change is not associated with acute changes in bilateral cerebral oxygenation in stable preterm infants during the first 3 days of life. American Journal of Perinatology 2014;32(7):645-651
86. Liu C-Z, Huang B-Y, Tan B-Y, et al. [Efficacy of volume-targeted ventilation for the treatment of neonatal respiratory distress syndrome]. Zhongguo dang dai er ke za zhi = Chinese journal of contemporary pediatrics 2016;18(1):6-9
87. Laughon M, Bose C, Moya F, et al. A pilot randomized, controlled trial of later treatment with a Peptide-containing, synthetic surfactant for the prevention of bronchopulmonary dysplasia [corrected] [published erratum appears in PEDIATRICS 2009 May;123(5):1436]. Pediatrics 2009;123(1):89-96
88. Long W, Corbet A, Cotton R, et al. A controlled trial of synthetic surfactant in infants weighing 1250 g or more with respiratory distress syndrome. The American Exosurf Neonatal Study Group I, and the Canadian Exosurf Neonatal Study Group. The New England journal of medicine 1991;325(24):1696-1703
89. Maruyama K, Fujiu T. Effects of prophylactic indomethacin on renal and intestinal blood flows in premature infants. Pediatrics international : official journal of the Japan Pediatric Society 2012;54(4):480-485
90. Mirzarahimi M, Barak M. Comparison efficacy of Curosurf and Survanta in preterm infants with respiratory distress syndrome. Pakistan journal of pharmaceutical sciences 2018;31(2):469-472
91. Mittendorf R, Dambrosia J, Dammann O, et al. Association between maternal serum ionized magnesium levels at delivery and neonatal intraventricular hemorrhage. The Journal of pediatrics 2002;140(5):540-546
92. Malhotra A, Fahey MC, Davies-Tuck M, et al. Comparison of preterm and term equivalent age MRI for the evaluation of preterm brain injury. Journal of Perinatology 2017;37(7):864-868
93. Ment LR, Oh W, Ehrenkranz RA, Allan WC. Antenatal exposure to steroids and the additional prophylaxis effect of indomethacin to prevent intraventricular hemorrhage. Pediatrics 1996;97(4):601

© 2023 Razak A et al. *JAMA Network Open.*

94. Mercer JS, Erickson-Owens DA, Vohr BR, et al. Effects of Placental Transfusion on Neonatal and 18 Month Outcomes in Preterm Infants: A Randomized Controlled Trial. The Journal of pediatrics 2016;168:50-55.e51
95. Mirzamoradi M, Kimyaiee P, Mansouri A, Bakhtiyari Z, Bakhtiyari M. The effect of magnesium sulfate in delaying delivery in premature rupture of membrane and its fetal complications. Iranian Journal of Obstetrics, Gynecology and Infertility 2014;17(127):1-9
96. Mitra S, Florez ID, Tamayo ME, et al. Effectiveness and safety of treatments used for the management of patent ductus arteriosus (PDA) in preterm infants: a protocol for a systematic review and network meta-analysis. BMJ open 2016;6(7):e011271
97. Nagano N, Saito M, Sugiura T, et al. Benefits of umbilical cord milking versus delayed cord clamping on neonatal outcomes in preterm infants: A systematic review and meta-analysis. PloS one 2018;13(8):e0201528
98. Nayeri FS, Esmaeilnia Shirvani T, Aminnezhad M, et al. Comparison of INSURE method with conventional mechanical ventilation after surfactant administration in preterm infants with respiratory distress syndrome: therapeutic challenge. Acta medica Iranica 2014;52(8):596-600
99. Neary E, Ni Ainle F, El-Khuffash A, et al. Plasma transfusion to prevent intraventricular haemorrhage in very preterm infants. Cochrane Database of Systematic Reviews 2016(9)
100. Nguyen T-MN, Crowther CA, Wilkinson D, Bain E. Magnesium sulphate for women at term for neuroprotection of the fetus. The Cochrane database of systematic reviews 2013(2):CD009395
101. Nicolaides KH, Syngelaki A, Poon LC, et al. Cervical pessary placement for prevention of preterm birth in unselected twin pregnancies: a randomized controlled trial. American journal of obstetrics and gynecology 2016;214(1):3.e1-9
102. Nijman TAJ, Goedhart MM, Naaktgeboren CN, et al. Effect of nifedipine and atosiban on perinatal brain injury: secondary analysis of the APOSTEL-III trial. Ultrasound in obstetrics & gynecology : the official journal of the International Society of Ultrasound in Obstetrics and Gynecology 2018;51(6):806-812

© 2023 Razak A et al. *JAMA Network Open.*

103. O'Sullivan D, O'Toole J, Finn D, et al. Multimodal monitoring as predictor of brain injury in the preterm infant. Archives of Disease in Childhood 2019;104:A24-A25
104. Oberthuer A. Less invasive surfactant application (LISA) in very immature newborns: State of the art. Journal of Perinatal Medicine 2017;45:188
105. Oei J-L, Saugstad OD, Vento M. Oxygen and preterm infant resuscitation: what else do we need to know? Current opinion in pediatrics 2018;30(2):192-198
106. Ohlsson A, Aher SM. Early erythropoiesis-stimulating agents in preterm or low birth weight infants. The Cochrane database of systematic reviews 2017;11:CD004863
107. Osborn DA, Evans N, Kluckow M. Effect of early targeted indomethacin on the ductus arteriosus and blood flow to the upper body and brain in the preterm infant. Archives of disease in childhood Fetal and neonatal edition 2003;88(6):F477-482
108. Ovalle A, Romero R, Gomez R, et al. Antibiotic administration to patients with preterm labor and intact membranes: is there a beneficial effect in patients with endocervical inflammation? The journal of maternal-fetal & neonatal medicine : the official journal of the European Association of Perinatal Medicine, the Federation of Asia and Oceania Perinatal Societies, the International Society of Perinatal Obstetricians 2006;19(8):453-464
109. Park WS, Ahn SY, Sung SI, Ahn J-Y, Chang YS. Mesenchymal Stem Cells: The Magic Cure for Intraventricular Hemorrhage? Cell transplantation 2017;26(3):439-448
110. Pasquier J-C, Claris O, Rabilloud M, et al. Intentional early delivery versus expectant management for preterm premature rupture of membranes at 28-32 weeks' gestation: A multicentre randomized controlled trial (MICADO STUDY). European journal of obstetrics, gynecology, and reproductive biology 2019;233:30-37
111. Peng W, Zhu H, Shi H, Liu E. Volume-targeted ventilation is more suitable than pressure-limited ventilation for preterm infants: A systematic review and meta-analysis. Archives of Disease in Childhood: Fetal and Neonatal Edition 2014;99(2):F158-F165

© 2023 Razak A et al. *JAMA Network Open.*

112. Perlman JM, Goodman S, Kreusser KL, Volpe JJ. Reduction in intraventricular hemorrhage by elimination of fluctuating cerebral blood-flow velocity in preterm infants with respiratory distress syndrome. The New England journal of medicine 1985;312(21):1353-1357
113. Qublan HS, Malkawi HY, Hiasat MS, et al. The effect of antenatal corticosteroid therapy on pregnancies complicated by premature rupture of membranes. Clinical and experimental obstetrics & gynecology 2001;28(3):183-186
114. Quinn MW, Wild J, Dean HG, et al. Randomised double-blind controlled trial of effect of morphine on catecholamine concentrations in ventilated pre-term babies. Lancet 1993;342 North American Edition(8867):324-327
115. Rennie JM, Doyle J, Cooke RW. Ethamsylate reduces immunoreactive prostacyclin metabolite in low birthweight infants with respiratory distress syndrome. Early human development 1986;14(3-4):239-244
116. Reynolds EO, Hope PL, Whitehead MD. Muscle relaxation and periventricular hemorrhage. The New England journal of medicine 1985;313(15):955-956
117. Rezaie M, Soofizadeh N, Saymari F, Vakili FZ. Comparison of the effect of single versus double doses of Betamethasone on the outcome of preterm neonates: A Clinical trial study. Research Journal of Pharmaceutical, Biological and Chemical Sciences 2016;7(6):874-879
118. Roberts D, Brown J, Medley N, Dalziel SR. Antenatal corticosteroids for accelerating fetal lung maturation for women at risk of preterm birth. Cochrane Database of Systematic Reviews 2017;2017(3)
119. Rojas MA, Lozano JM, Rojas MX, et al. Very early surfactant without mandatory ventilation in premature infants treated with early continuous positive airway pressure: a randomized, controlled trial. Pediatrics 2009;123(1):137-142
120. Romero R, Sibai B, Caritis S, et al. Antibiotic treatment of preterm labor with intact membranes: a multicenter, randomized, double-blinded, placebo-controlled trial. American journal of obstetrics and gynecology 1993;169(4):764-774
121. Rook D, Schierbeek H, van der Eijk AC, et al. Resuscitation of very preterm infants with 30% vs. 65% oxygen at birth: study protocol for a randomized controlled trial. Trials 2012;13:65

© 2023 Razak A et al. *JAMA Network Open.*

122. Ruegger CM, Hagmann CF, Buhrer C, et al. Erythropoietin for the Repair of Cerebral Injury in Very Preterm Infants (EpoRepair). Neonatology 2015;108(3):198-204
123. Saugstad OD. Oxygenation of the newborn-what is new? Journal of Perinatal Medicine 2017;45:222
124. Scheller JM, Nelson KB. Does cesarean delivery prevent cerebral palsy or other neurologic problems of childhood? Obstetrics and gynecology 1994;83(4):624-630
125. Schmidt B, Davis P, Moddemann D, et al. Long-term effects of indomethacin prophylaxis in extremely-low-birth-weight infants. The New England journal of medicine 2001;344(26):1966-1972
126. Schmölzer GM, O Reilly M, Fray C, van Os S, Cheung P-Y. Chest compression during sustained inflation versus 3:1 chest compression:ventilation ratio during neonatal cardiopulmonary resuscitation: a randomised feasibility trial. Archives of Disease in Childhood - Fetal and Neonatal Edition 2018;103(5):F455
127. Schulte J, Osborne J, Benson JWT, et al. Developmental outcome of the use of etamsylate for prevention of periventricular haemorrhage in a randomised controlled trial. Archives of disease in childhood Fetal and neonatal edition 2005;90(1):F31-35
128. Shah PS, Shah VS. Continuous heparin infusion to prevent thrombosis and catheter occlusion in neonates with peripherally placed percutaneous central venous catheters. The Cochrane database of systematic reviews 2008(2):CD002772
129. Shankaran S, Bauer CR, Bain R, Wright LL, Zachary J. Relationship between antenatal steroid administration and grades III and IV intracranial hemorrhage in low birth weight infants. The NICHD Neonatal Research Network. American journal of obstetrics and gynecology 1995;173(1):305-312
130. Sharafutdinova DR, Balashova EN, Ionov OV, et al. The recombinant human erythropoietin therapy for extremely and very low birth weight infants. Pediatric Hematology/Oncology and Immunopathology 2019;18(2):75-82
131. Shirk SK, Manolis S, Lambers D, Smith K. 93: Delayed clamping versus milking of umbilical cord in preterm infants, a randomized controlled trial. American Journal of Obstetrics and Gynecology 2019;220(1):S75-S76

© 2023 Razak A et al. *JAMA Network Open.*

132. Silver RK, Vyskocil C, Solomon SL, et al. Randomized trial of antenatal dexamethasone in surfactant-treated infants delivered before 30 weeks' gestation. Obstetrics and gynecology 1996;87(5 Pt 1):683-691
133. Simons SHP, Roofthooft DWE, van Dijk M, et al. Morphine in ventilated neonates: its effects on arterial blood pressure. Archives of disease in childhood Fetal and neonatal edition 2006;91(1):F46-51
134. Sinha SK, Lacaze-Masmonteil T, Valls i Soler A, et al. A multicenter, randomized, controlled trial of lucinactant versus poractant alfa among very premature infants at high risk for respiratory distress syndrome. Pediatrics 2005;115(4):1030-1038
135. Sluncheva B, Vakrilova L, Emilova Z, Kalaidzhieva M, Garnizov T. [Prevention of brain hemorrhage in infants with low and extremely low birth weight and infants treated with surfactants. Late observation]. Akusherstvo i ginekologiia 2006;45(3):34-38
136. Skoll A, Boutin A, Bujold E, et al. No. 364-Antenatal Corticosteroid Therapy for Improving Neonatal Outcomes. Journal of obstetrics and gynaecology Canada : JOGC = Journal d'obstetrique et gynecologie du Canada : JOGC 2018;40(9):1219-1239
137. Smit E, Odd D, Whitelaw A. Postnatal phenobarbital for the prevention of intraventricular haemorrhage in preterm infants. The Cochrane database of systematic reviews 2013(8):CD001691
138. Smith GN, Walker MC, Ohlsson A, et al. Randomized double-blind placebo-controlled trial of transdermal nitroglycerin for preterm labor. American journal of obstetrics and gynecology 2007;196(1):37.e31-38
139. Sosenko IRS, Fajardo MF, Claure N, Bancalari E. Timing of patent ductus arteriosus treatment and respiratory outcome in premature infants: a double-blind randomized controlled trial. The Journal of pediatrics 2012;160(6):929-935.e921
140. Sommers R, Stonestreet BS, Oh W, et al. Hemodynamic effects of delayed cord clamping in premature infants. Pediatrics 2012;129(3):e667-672

© 2023 Razak A et al. *JAMA Network Open.*

141. Stock SJ, Bricker L, Norman JE. Immediate versus deferred delivery of the preterm baby with suspected fetal compromise for improving outcomes. Cochrane Database of Systematic Reviews 2012(7):N.PAG-N.PAG
142. Strickland DM, Wallach RC, Campbell WA. Antenatal betamethasone compared with dexamethasone (betacode trial): a randomized controlled trial...Obstet Gynecol. 2007 Jul;110(1):26-30; Obstet Gynecol. 2007 Jul;110(1):26-30. Obstetrics & Gynecology 2007;110(4):930-931
143. Su P-H, Chen J-Y, Su C-M, Huang T-C, Lee H-S. Comparison of ibuprofen and indomethacin therapy for patent ductus arteriosus in preterm infants. Pediatrics international : official journal of the Japan Pediatric Society 2003;45(6):665-670
144. Subhedar NV, Shaw NJ. Dopamine versus dobutamine for hypotensive preterm infants. The Cochrane database of systematic reviews 2003(3):CD001242
145. Supapannachart S, Khowsathit P, Patchakapati B. Indomethacin prophylaxis for patent ductus arteriosus (PDA) in infants with a birth weight of less than 1250 grams. Journal of the Medical Association of Thailand = Chotmaihet thangphaet 1999;82 Suppl 1:S87-92
146. Thomas RL, Parker GC, Van Overmeire B, Aranda JV. A meta-analysis of ibuprofen versus indomethacin for closure of patent ductus arteriosus. European journal of pediatrics 2005;164(3):135-140
147. Thome UH, Carlo WA. High-frequency ventilation in neonates. American journal of perinatology 2000;17(1):1-9
148. Thome UH, Dreyhaupt J, Genzel-Boroviczeny O, et al. Influence of PCO2 Control on Clinical and Neurodevelopmental Outcomes of Extremely Low Birth Weight Infants. Neonatology 2018;113(3):221-230
149. Unal S, Ergenekon E, Aktas S, et al. Effects of Volume Guaranteed Ventilation Combined with Two Different Modes in Preterm Infants. Respiratory care 2017;62(12):1525-1532
150. van Teeffelen ASP, van der Ham DP, Willekes C, et al. Midtrimester preterm prelabour rupture of membranes (PPROM): expectant management or amnioinfusion for improving perinatal outcomes (PPROMEXIL - III trial). BMC pregnancy and childbirth 2014;14:128

© 2023 Razak A et al. *JAMA Network Open.*

151. Van Overmeire B, Van de Broek H, Van Laer P, Weyler J, Vanhaesebrouck P. Early versus late indomethacin treatment for patent ductus arteriosus in premature infants with respiratory distress syndrome. The Journal of pediatrics 2001;138(2):205-211
152. Veldman A, Josef J, Fischer D, Volk WR. A prospective pilot study of prophylactic treatment of preterm neonates with recombinant activated factor VII during the first 72 hours of life. Pediatric critical care medicine : a journal of the Society of Critical Care Medicine and the World Federation of Pediatric Intensive and Critical Care Societies 2006;7(1):34-39
153. Waltl H, Fodisch HJ, Kurz R, et al. Intracranial haemorrhage in low-birth-weight infants and prophylactic administration of coagulation-factor concentrate. Lancet (London, England) 1973;1(7815):1284-1286
154. Wang H, Zhang L, Jin Y. A meta-analysis of the protective effect of recombinant human erythropoietin (rhEPO) for neurodevelopment in preterm infants. Cell biochemistry and biophysics 2015;71(2):795-802
155. Wardle SP, Hughes A, Chen S, Shaw NJ. Randomised controlled trial of oral vitamin A supplementation in preterm infants to prevent chronic lung disease. Archives of disease in childhood Fetal and neonatal edition 2001;84(1):F9-F13
156. Wenstrom KD. [Commentary on] Randomized trial of a single repeat dose of prenatal betamethasone treatment in imminent preterm birth. Obstetrical & Gynecological Survey 2007;62(6):368-370
157. Wheeler KI, Klingenberg C, Morley CJ, Davis PG. Volume-targeted versus pressure-limited ventilation for preterm infants: a systematic review and meta-analysis. Neonatology 2011;100(3):219-227
158. Whyte R, Kirpalani H. Low versus high haemoglobin concentration threshold for blood transfusion for preventing morbidity and mortality in very low birth weight infants. Cochrane Database of Systematic Reviews 2011(11)
159. Thorp JA, Ferrette-Smith D, Gaston LA, et al. Combined antenatal vitamin K and phenobarbital therapy for preventing intracranial hemorrhage in newborns less than 34 weeks' gestation. Obstetrics and gynecology 1995;86(1):1-8

© 2023 Razak A et al. *JAMA Network Open.*

160. Weesner KM, Dillard RG, Boyle RJ, Block SM. Prophylactic treatment of asymptomatic patent ductus arteriosus in premature infants with respiratory distress syndrome. Southern medical journal 1987;80(6):706-708
161. Wyllie JP, Gupta S. Prophylactic and early targeted treatment of patent ductus arteriosus. Semin Fetal Neonatal Med 2018;23(4):250-254
162. Xu YJ, Ran LM, Zhai SS, et al. Evaluation of the efficacy of atosiban in pregnant women with threatened preterm labor associated with assisted reproductive technology. European review for medical and pharmacological sciences 2016;20(9):1881-1887
163. Yadav S, Agarwal S, Maria A, et al. Comparison of oral ibuprofen with oral indomethacin for PDA closure in Indian preterm neonates: a randomized controlled trial. Pediatric cardiology 2014;35(5):824-830
164. Yang S-S, Xu F-L, Cheng H-Q, et al. [Effect of early application of recombinant human erythropoietin on white matter development in preterm infants]. Zhongguo dang dai er ke za zhi = Chinese journal of contemporary pediatrics 2018;20(5):346-351
165. Zeng L, Choonara I, Zhang L, Li Y, Shi J. Effectiveness of prothrombin complex concentrate (PCC) in neonates and infants with bleeding or risk of bleeding: a systematic review and meta-analysis. European journal of pediatrics 2017;176(5):581-589
166. Sunil B, Sindhuraj MN, Athul P. Umbilical Cord Milking in Late Preterm Neonates and its Effects on Haematological and Haemodynamical Parameters-A Randomised Controlled Study. Journal of Clinical and Diagnostic Research 2022;16(3):SC16-SC19
167. McDonald S, Tomlinson G, Dodd J, et al. Single Dose of Antenatal Corticosteroids (SNACS) Non-Inferiority Randomized Controlled Trial for Pregnancies at Risk of Preterm Delivery. Journal of Obstetrics and Gynaecology Canada 2022;44(5):617
168. Benavides A, Conrad AL, Brumbaugh JE, et al. Long-term outcome of brain structure in female preterm infants: possible associations of liberal versus restrictive red blood cell transfusions. Journal of Maternal-Fetal and Neonatal Medicine 2021;34(20):3292-3299

© 2023 Razak A et al. *JAMA Network Open.*

169. Bravo MC, Sanchez-Salmador R, Moral-Pumarega MT, et al. Randomized Trial on Echocardiography-Guided Ductus Arteriosus Treatment to Reduce Necrotizing Enterocolitis. Frontiers in Pediatrics 2022;9:807712
170. Erdeve O, Okulu E, Roberts KD, et al. Alternative Methods of Surfactant Administration in Preterm Infants with Respiratory Distress Syndrome: State of the Art. Turkish Archives of Pediatrics 2021;56(6):553-562
171. Mari A, Bawa M, Gugino S, et al. INITIAL ELEVATED MIDLINE HEAD POSITION REDUCES HEMODYNAMIC FLUCTUATION WITH SUBSEQUENT POSITION CHANGE. Journal of Investigative Medicine 2022;70(4):1092-1093
172. Li XL, Cai YJ, Zhang Z, et al. Effect of different maintenance doses of caffeine citrate on ventilator weaning in very preterm infants with respiratory distress syndrome: A prospective randomized controlled trial. Chinese Journal of Contemporary Pediatrics 2021;23(11):1097-1102
173. Sabzehei MK, Basiri B, Shokouhi M, Ghahremani S, Moradi A. Comparison of minimally invasive surfactant therapy with intubation surfactant administration and extubation for treating preterm infants with respiratory distress syndrome: a randomized clinical trial. Clinical and Experimental Pediatrics 2022;65(4):188-193
174. Iranpour R, Armanian AM, Miladi N, Feizi A. Effect of Prophylactic Caffeine on Noninvasive Respiratory Support in Preterm Neonates Weighing 1250-2000 g: A Randomized Controlled Trial. Archives of Iranian Medicine 2022;25(2):98-104

© 2023 Razak A et al. *JAMA Network Open.*

e-Table 1: Characteristics of studies studying interventions on severe brain injury in preterm infants

[redacted]											
					Random sequence generation	Allocation concealment	Blinding of participants	Blinding of outcome	Incomplete outcome	Selective reporting	Other biases
**Antenatal Interventions**											
**Antenatal corticosteroids for lung maturity**											
WHO Trial 2020	Multicenter	26-34wk	Dexa X 4, 6mg Q12hr	Placebo/NS	L	L	L	L	L	L	L
Gyamfi 2016	USA	34-36wk	Beta X 2, 12mg Q24hr	Placebo	L	L	L	L	L	L	L
Garite 2009	Multicenter	25-34wk	Beta X 2, 12mg Q24hr	Placebo	L	L	L	U	L	L	L
Peltoniemi 2007	Finland	<34wk	Beta X 2, 12mg Q24hr, Repeat course >7days	Placebo/NS	L	L	L	L	L	L	L
Lewis 1996	USA	24-34wk	Beta X 2, 12mg Q24hr	No treatment	L	L	H	U	L	L	U
Silver 1996	USA	24-29wk	Dexa X4, 5 mg 12hr	NS	L	L	L	L	H	L	U
Kari 1994	Finland	24-32wk	Dexa X 4, 6mg Q12hr	Placebo	U	U	L	U	L	L	U
Garite 1992	USA	24-28wk	Beta Acetate 6mg+ Beta phosphate 6mg , Q24hr	NS	L	U	L	U	L	L	U
Morales 1989	USA	26-34wk	Beta X 2, 12mg Q24hr	No treatment	U	U	H	U	L	L	U
Morales 1986	USA	28-33wk	Dexa X 4, 6mg Q12hr	No treatment	L	L	U	L	L	U	U
**Dexamethasone *vs.* Betamethasone for lung maturity**											
Crowther 2019	Multicenter	<34wk	Dexa X 4, 6mg Q12hr	Beta X 2, 12mg Q24hr	L	L	L	L	L	L	L
Elimian 2007	USA	24-34wk	Dexa X 4, 6mg Q12hr	Beta X 2, 12mg Q24hr	L	L	L	L	U	L	L
Chen 2005	Taiwan	24-34wk	Dexa X 4, 6mg Q12hr	Beta X 2, 12mg Q24hr	U	U	U	U	H	U	U
Subtil 2003	France	27-35wk	Dexa X 4, 6mg Q12hr	Beta X 2, 12mg Q24hr	L	U	H	H	U	L	L

© 2023 Razak A et al. *JAMA Network Open.*

**Repeat antenatal steroids *vs.* no repeat antenatal steroids**											
Garite 2009	Multicenter	25-33wk	Beta (Repeat course)	NS	L	L	L	U	L	L	L
Murphy 2008	Multicenter	25-32wk	Beta x Q14 day till 33wk	Placebo	U	L	L	U	L	L	L
Peltoniemi 2007	Finland	<34wk	Beta (Repeat)	Placebo	L	L	L	L	L	L	H
Crowther 2006	Multicenter	<32 wk	Celestone (Repeat till delivery or 32wk)	NS	L	L	L	U	L	L	L
Wapner 2006	Multicenter	23-32wk	Beta (Repeat once)	Placebo	L	L	L	L	L	L	H
Lee 2004	Multicenter	24-33wk	Beta x till 34wk	Placebo	L	L	L	U	L	L	L
Asghajafari 2002	Canada	24-30wk	Beta x till 33wk	NS	L	L	L	U	L	U	L
Guinn 2001	Multicenter	24-33wk	Beta or Dexa Q wkly till 34 wk	Single course Beta or Dexa	L	L	L	U	L	L	H
**Magnesium sulfate for tocolysis or neuroprotection**											
Crowther 2003	Australia	<30wk	MgSO4	NS	L	L	L	L	L	L	L
Rouse 2008 (BEAM)	USA	24-31wk	MgSO4	Placebo	L	L	L	L	L	L	L
Marret 2006 (PREMAG)	France	<33wk	MgSO4	NS	L	L	U	L	L	L	L
Parashi 2017	Iran	<33wk.	MgSO4	NS	L	L	L	L	L	L	L
Fox 1993	USA	34-37wk	MgSO4	Placebo	L	L	H	U	L	L	L
How 1998	USA	24-34wk	MgSO4	Placebo	L	U	U	U	L	U	L
**Antibiotics for premature rupture of membranes**											
Fuhr 2006	Multicenter	24-33wk	Mezlocillin	Placebo	U	U	L	U	L	U	U
Mercer 1997	Multicenter	24-32wk	Ampicilln+Erythromycin	Placebo	L	L	L	L	L	U	U
Grable 1996	USA	<35wk	Ampicillin	Placebo	L	L	L	L	L	U	L
Owen1993	USA	24-34wk	Ampicillin	Not specified	L	L	H	U	L	U	L
**Cesarean section *vs.* vaginal delivery for preterm birth**											
No RCT											
**Delivery Room Interventions**											
**Lower vs. higher FiO2 for resuscitation**			**FiO2**	**FiO2**							
Oie 2017	Australia	<32 wk	21-100%	100%	L	L	H	L	U	L	L
Aguar 2013	Multicenter	<28wk	30%	60%	L	L	H	L	L	L	L
Boronat 2013	Spain	<30wk	30%	60-65%	L	L	H	L	L	L	L
Kapadia 2013	USA	24-35w	21-100%	100%	L	L	H	U	L	H	L
Vento 2009	Spain	<28wk	21-100%	100%	L	L	H	U	L	L	L
Esrig 2008	Spain	<28wk	30%	90%	L	L	H	L	U	U	L
Wang 2008	USA	23-32wk	21%	100%	U	U	H	U	L	L	L

© 2023 Razak A et al. *JAMA Network Open.*

Lundstrom 1995	Denmark	<33wk	21%	80%	U	U	H	U	L	L	L
**Sustained lung inflation (SLI) *vs.* standard resuscitation**											
Hamd 2020	Egypt	<27-32 wk	SLI 20cm for 15sec, then CPAP 5.	CPAP 5	L	U	U	U	H	H	L
Kirpalani 2019	Multicenter	23-27wk	SLI, 20cm, 15sec, later SLI 25cm 15sec.	IPPV with PEEP	L	L	H	U	L	L	U
Laverde 2019	Italy	28-31wk	SLI 25cm for 15 sec, CPAP 5.	PPV 25/5 R 40-60	U	U	U	U	L	U	L
El Fattah 2017	Egypt	<32wk	SLI 15,20cm for10,20sec each.	CPAP 5	L	U	U	U	L	L	L
Jiravisitkul 2017	Thailand	25-32wk	SLI 25cm 15s, later CPAP 6, 2nd SLI	PIP 15-20cm, PEEP5,	L	L	H	U	L	U	L
Nagan 2017	Canada	23-33wk	2PIP 24cm 20sec 2nd SLI 20 20sec	IPPV later CPAP 5cm	L	U	H	L	H	L	U
Schwaberger 2015	Austria	28-34wk	PIP 30cm,15sec, Rpt 1 or 2 HR <100. CPAP, HR >100.	IPPV, later CPAP	L	L	H	L	L	L	L
Lista 2015	Multicenter	25-29wk	PIP 25cm 15sec, CPAP 5.PIP 20cm 15sec	CPAP 5 or PIP	U	L	H	L	L	L	L
Linder 2015	Germany	25-29wk	SLI 25-30 sec, PIP 20cm, PEEP 4-6	CPAP 5	U	L	H	U	L	L	U
**Delayed cord clamping *vs.* early cord clamping**											
Yunis 2020	Egypt	<34wk	DCC 60sec	ECC 10sec	L	L	H	U	L	L	L
Hemmati 2020	Iran	26-34wk	DCC 30-45sec	ECC 10-15	L	U	H	L	H	L	L
Finn 2019(CUPiD)	Ireland	<32wk	DCC 60sec	ECC 20sec	L	L	H	L	L	L	L
Duley L 2018 (Cord Pilot Trial)	UK	<32wk	DCC 2min Resus bedside	ECC 20sec	L	L	H	L	L	L	L
Armanian 2017	Iran	<34wk	DCC 30-45sec	ECC 5-10sec	L	L	H	U	L	L	U
Tarnow Mordi 2017	Multicenter	<32wk	DCC 60sec	ECC 10sec	L	L	H	U	L	L	L
Backes 2016	USA	23-28wk	DCC 30-45sec	ECC 10sec	L	L	H	L	L	L	L
Dong 2016	China	25-32wk	DCC with 10-20cm below	ECC 10sec	U	U	H	U	L	L	
Kugelman 2007	Israel	24-35wk	DCC 30-45sec	ECC 10sec	U	U	H	U	L	U	U
Mercer 2006	USA	24-32wk	DCC 30-45sec, 10-15 inches below	ECC <10sec	L	L	H	L	L	L	L
Mercer 2003	USA	24-32wk	DCC 30-45sec	ECC 10sec	U	U	H	L	L	L	U
Rabe 2000	Germany	<33wk	DCC 45sec	ECC 20sec	L	L	H	U	L	U	L

© 2023 Razak A et al. *JAMA Network Open.*

Hofmeyr 1998	SA	<35wk	DCC 60sec	ECC <10sec	U	U	H	L	L	L	U
Hofmeyr 1993	SA	<2000g	DCC	ECC	U	U	H	L	L	U	L
Kelly 2021	Canada	24-32wk	DCC 30-45 sec	ECC 10 sec	L	L	H	U	L	L	L
**Umbilical cord milking *vs.* early cord clamping**											
EL Naggar 2020	Canada	<32wk	UCM 10cm/s x3	ECC<10sec	L	L	H	L	L	L	L
Finn 2019(CUPiD)	Ireland	<32wk	UCM+DCC	ECC 20sec	L	L	H	L	L	L	L
Josephsen 2020	USA	24-28wk	UCMX3 2sec	ECC<10sec	U	U	H	U	L	U	U
Juan 2018	China	28-37wk	UCM 20cmX4	ECC	U	U	H	U	L	U	L
Rammohan 2018	India	<37wk	UCMX3 25cm	ECC <10sec	L	L	H	H	L	L	L
Song 2017	China	24-35wk	UCMX4 2sec below introitus (15-20sec)	ECC<10sec	L	U	H	L	L	L	L
Mercer 2016	USA	24-32wk	DCC30-40sec	ECC<10sec	U	U	H	L	L	L	U
Alan 2014	Turkey	<32wk	UCMX3, 25-30cm	ECC<10sec	U	U	H	U	L	L	L
Eliman 2014	USA	24-34wk	UCMX3, DCC 30sec	ECC <5sec	L	L	H	L	L	L	L
Katheria 2014	USA	23-32wk	UCMX3 2 sec	ECC <10sec	L	L	H	L	L	L	L
March 2013	USA	24-28wk	UCMX3	ECC <10sec	L	L	H	U	L	L	L
Hosno 2008	Japan	24-28wk	UCMX2-3	ECC <10sec	U	L	H	U	L	L	L
**Umbilical cord milking *vs.* delayed cord clamping**											
Katheria 2020	Multicenter	23-28wk	UCMX3, 20cm	DCC 60sec	L	L	H	U	L	H	H
Katheria 2019	Multicenter	23-32wk	UCMX3,20cm DCC 60sec	DCC 60sec	L	L	H	L	L	L	H
Finn 2019(CUPiD)	Ireland	<32wk	UCM	DCC	L	L	H	L	L	L	L
Katheria 2015	USA	23-32wk	UCM X4, 20cm,	DCC 45sec	L	L	H	L	L	L	U
Rabe 2011	UK	24-32wk	UCM X4, 20cm	DCC 30sec	L	L	H	U	L	L	U
Bichkar 2019	India	<32wk	UCM x4, 20cm	DCC 30sec	L	L	H	U	L	L	L
**Delayed cord clamping with respiratory support *vs.* delayed cord clamping without respiratory support**											
Katheria 2016	USA	23-32wk	DCC 60sec, CPAP/PPV	DCC	L	L	H	U	L	L	L
**Postnatal Interventions**											
**Head midline position**											
Kochan 2019	USA	<1000g	Supine head elevated 30^0	Supine flat lateral	L	L	H	L	L	U	L
Al Abdi 2015	Saudi Arabia	<30wk	Flat midline head 168hrs	Supine flat lateral	U	U	H	L	L	L	H
Al Abdi 2011	Saudi Arabia	<30wk	Flat midline, chin at 90^0	Supine flat lateral	L	L	H	L	L	L	U

© 2023 Razak A et al. *JAMA Network Open.*

**LISA *vs.* INSURE**											
Han 2020	Multicenter	<32wk	MISA with 5Fr gastric tube.	Surfactant via ETT	L	L	H	H	L	L	L
Bao 2015	China	28-32wk	16G catheter marked to desired depth, X 5 bolus, Later CPAP	Surfactant via ETT	U	U	H	U	L	U	L
Kribs 2015	Multicenter	23-27wk	4Fr catheter attached to a syringe, breathing on CPAP.	Surfactant via ETT	L	L	H	H	L	L	L
Gopel 2011	Multicenter	26-30wk	Spont breathing, CPAP, Surf. using thin catheter.	Rescue surfactant	L	U	H	H	L	L	L
Jena 2019	India	<34 wk	16G Angiocath or 6F feeding tube, Spont breathing.	Surfactant via ETT	L	L	L	U	L	U	L
Gupta 2020	India	24-34wk	MISA with 5Fr gastric tube.	Surfactant via ETT	L	L	H	U	L	L	L
**Volume guarantee *vs.* Conventional ventilation**											

© 2023 Razak A et al. *JAMA Network Open.*

Krishna 2019	India	27-34wk	SIMV+VG 5ml/kg, PEEP 4-6cm H2O	SIMV+PC, PIP 14-18, PEEP 4-6cm	L	L	U	U	L	U	U
Chowdhury	UK	<34wk	SIMV+VT 5ml/kg	SIMV	L	L	H	U	L	L	H
Guven 2013	Turkey	<32wk	SIMV+VG4-5ml/kg	SIMV+PIP 20-25	L	U	H	U	L	U	H
Duman 2012	Turkey	<32wk	VG 4ml/kg PEEP 4-6	PLV+PIP	L	L	H	U	L	L	L
Liu 2011	China	24-32wk	SIPPV+VG 4-6ml/kg	IMV, PIP 20-25	U	U	H	U	L	U	L
Piotrowski 2007	Poland	RDS +	PRVC, VT 5-6 ml/kg	PLV, PEEP3-5	L	L	H	H	L	U	H
Singh 2006	UK	600-1500g	VG 8-10ml/kg, PEEP 3-5cm H2O	SIMV, PEEP 3-5ml/kg	L	L	H	U	L	U	U
D Angio 2005	USA	500-1249g	PRVC,? VT	PLV, PIP (VT 4-6)	L	L	H	U	L	U	L
Nafday 2005	USA	<1500g	PSV-VG 5ml/kg	PLV + SIMV	L	L	U	U	L	U	L
Keszler 2004	USA	<32wk	A/C+VG	A/C	L	L	H	H	L	U	L
Lista 2004	Italy	25-32wk	PSV-VG 5ml/kg PEEP 3.5-4cm H2O	PSV, PEEP 3.5-4cm H2O	L	L	H	U	L	U	H
Piotrowski 1997	Poland	<2500g	PRVC VT 5-6ml/kg	PLV PIP, PEEP 3-5cm H2O	U	L	H	L	L	U	L
Sinha 1997	USA	1200g	VT 5-8ml/kg	PLV PIP (5-8ml/kg)	U	U	H	L	L	U	U
**Elective HFOV *vs.* Conventional ventilation**											
Sun 2014	China	<32wk, <1500g	HFOV MAP 6-8 Increase gradually, 10Hz	SIMV-PSV VT 4-6, PEEP 4-6	L	U	H	U	L	H	L
Salvo 2012	Multicenter	<30wk <1500gg	HFOV 15hz, MAP 6-8, AMP good wiggle.	SIPPV PIP 18-24, PEEP 5-8.	L	U	H	U	L	L	U
Lista 2008	Italy	25-32wk	HFOV MAP 8-10, 10 Hz, Amp 40%.	CV-VG 5ml/kg, PEEP 5.	L	U	H	U	L	L	L
Vento 2005	Italy	24-29wk 500-1000g	MAP 2cm > CV, 10Hz Amp 30%.	SIMV VT 4-6, PEEP 4-5.	L	L	H	U	L	L	L
Schrieber 2003	USA	23-34wk	MAP~ Lung Inflation, Hz10-12.	PIP 16-24, PEEP 4-6.	U	U	H	U	L	L	U
Craft 2003	USA	23-34wk	MAP-optimal lung vol, Hz 10-12.	PIP-good chest rise, PEEP 4-6.	L	L	H	U	L	L	U

© 2023 Razak A et al. *JAMA Network Open.*

Van Reempts 2003	Germany	<32wk	MAP 8-10, Hz 10, Amp-good wiggle.	CV-PIP 20, PEEP 4-6.	U	U	H	L	L	L	L
Courtney 2002	USA	600-1220g	MAP>2 CV, Hz 10-15, Amp good wiggle.	VT 4-7ml/kg, PEEP 4-6.	U	U	H	L	L	L	L
Johnson 2002	Multicenter	<29wk	MAP 8-10, Hz 10, Amp-good wiggle.	CV Rate 60	U	U	H	L	L	L	L
Durand 2001	USA	501-1200g	MAP>2 CV, Hz 10-15, Amp-depends on PCO2.	TV 5-6ml/kg, PEEP 4-6.	U	U	H	L	L	L	U
Moriette 2001	France	24-29wk.	MAP>2 CV, Hz 15, high vol strategy.	SIMV-PIP target TV. PEEP 4-5.	L	L	H	L	L	L	L
Plavka 1999	Czech Republic	500-1499g, <31wk	MAP-step wise increase, Hz 15.	CV-PIP chest rise, PEEP 3-5.	L	L	H	U	L	L	L
Thome 1999	Germany	24-<30wk	MAP>1-2 CV, Hz 10, Amp good wiggle.	IPPV, PIP adjust, PEEP >3, rate 60.	U	L	H	U	L	L	L
Rettwitz-Volk 1998	Multicenter	750-1500g	MAP and Amp- chest rise/Spo2, Hz 15-20.	PIP-chest rise, PEEP 3-4.	L	U	H	U	L	L	L
Gerstmann 1996	Multicenter	<35wk	MAP 1-2cm H2O>CV, I:E 0.33, 10-15Hz.	IMV, PIP 30-35, PEEP 3-7.	L	U	H	U	L	L	H
Ogawa 1993	Czech Republic	500-1499g, <31wk	MAP step wise increase HZ10-15, Amp wiggle.	CV, PIP-chest rise, PEEP 3-5.	L	L	H	U	L	L	L
HiFO study Group 1993	Multicenter	>=500g with sRDS	MAP 2-3cm > CV, Hz15 Amp-adequate.	CV-PIP chest rise, PEEP 4-6.	L	U	H	U	L	U	L
Clark 1992	USA	<35wk, <1751g	MAP 1-2cm H2O above CV, 10Hz.	CV-PIP20-27, PEEP 4-6.	L	U	H	U	L	U	L
HIFI 1989	Multicenter	750-2000g	MAP= or < CV, or 8-10cm,15Hz, AMP chest oscillation	PEEP2-5cm H2O, PIP 20-25cm.	L	U	H	U	L	L	L
**Elective HFJV *vs.* Conventional ventilation**											
Keszler 1997	Multicenter	<36wk	PEEP for recruitment, intermittent sigh breath. PEEP increased, thereby result in MAP inc 0.5-2cm H2O	CV-R 30-60/min, iT 0.3-0.4S, PEEP 4-6 cm H2O	L	L	H	U	L	L	H
Wiswell 1996	USA	<33wk	Freq 420/min, jet time 0.02sec PIP 80-90% of PIP on CV. PEEP 4-5cm H2O,5-10 back up breaths.	CV (setting not mentioned)	U	U	H	L	L	L	L

© 2023 Razak A et al. *JAMA Network Open.*

**Oxygen saturation target after birth: 85-89% *vs.* 91-95%.**			**Target SpO2**	**Target SpO2**							
BOOST-II Australia 2016	Australia	<28wk	91-95%	85-89%	L	L	L	L	L	L	U
BOOST-II UK 2016	UK	<24hr	91-95%	85-89%	L	L	L	L	L	L	U
BOOST-II NZ 2014	New Zealand	<24hr	91-95%	85-89%	L	L	L	L	L	L	L
Schmidt (COT) 2013	Multicenter	<28wk	91-95%	85-89%	L	L	L	L	L	L	L
Vaucher (Support) 2010	Multicenter	<24hr	91-95%	85-89%	L	L	L	L	L	L	L
**Permissive hypercapnia *vs.* normocapnia**											
Thome 2015	Multicenter	23-29wk	PaCO2 55-65 (1-3 days)	PaCO2 40-50 (1-3 days)	L	U	H	L	H	L	H
Thome 2006	USA	23-29wk	PaCO2 55-65 (7 days)	PaCO2 35-45 (7 days)	L	L	H	U	H	U	H
Carlo 2002	Multicenter	501-1000g	PaCO2 >52	PaCO2 <48	L	U	U	U	U	U	H
Carlo 1999	Multicenter	501-1000g	PaCO2 >52, 10 days	PaCO2 <48, 10 days	U	U	U	U	U	U	H
Mariani 1999	USA	601-1250g	PCO2 45-55, 96hr	PCO2 35-45, 96 hr	L	L	U	U	L	L	L
**Early extubation *vs.* delayed extubation**											
Denan 2008	France	24-27wk	Extubated immediately (criteria met)	Delayed extubation 36 hr	L	U	H	U	L	U	L
**Caffeine prophylaxis or treatment for apnea or post-extubation**											
Amaro 2018	USA	23-30wk	Caffeine L- 20mg/kg, M-5mg/kg.	NS	L	U	L	L	L	L	H
CAP Trial 2006	Multicenter	500-1250g	Caffeine L- 20mg/kg, M-5mg/kg.	NS	L	L	L	L	L	L	L
Ramin 2022	Iran	1500-2000g	Caffeine L- 20mg/kg, M-10mg/kg.	No treatment	U	U	H	U	L	L	U
**High dose *vs.* low dose caffeine for apnea or post-extubation**											

© 2023 Razak A et al. *JAMA Network Open.*

Mohd Kori 2021	Malaysia	<32wk	L-Caff 40mg/kg, M-20mg/kg.	L-Caff 20mg /kg, M-10mg/kg maintenance.	L	L	L	U	L	L	L
Vesoulis 2018	USA	<30wk	L-Caff 80mg/kg (36hr), M-10mg/kg.	L-Caff 30mg /kg (36hrs), M-10mg/kg.	L	L	L	L	L	L	L
Mohammad 2015	Egypt	<32wk	L-Caff 40mg/kg, M-20mg/kg.	L-Caff 20mg /kg, M-10mg/kg.	L	U	L	L	L	L	L
McPherson 2015	USA	24-30wk	L-Caff 40mg/kg, 20mg/kg (12hr), M-10mg/kg (24hr, 36hr).	L-Caff 20mg /kg, M-10mg/kg.	L	U	L	L	H	H	L
Gray 2010	Australia	<30wk	L-Caff 80mg/kg, M-20mg/kg .	L-Caff 20mg /kg, M-5 mg/kg.	L	L	L	U	L	L	L
Steer 2003	Australia	<32wk	L-Caff 30mg/kg, M-1mg/kg	L-Caff 3mg /kg, M-1mg/kg	L	L	L	U	U	U	H
**Sedation during ventilation: opioids *vs.* placebo**											
Anand 1999	Multicenter	24-32wk	Morphine 200mc/kg 20,40,60 mc/kg/hr maintenance.	Dextrose 10%	L	L	L	U	L	U	L
Anand 2004	Multicenter	23-32wk	Morphine 100mc/kg 10, 20,30 mc/kg/hr maintenance.	Placebo	L	L	L	L	L	L	L
Simons 2003	Netherlands	23-37wk <3days	Morphine 100mc/kg Later 10mc/kg/hr for 7 days.	Saline+5% Dextrose	L	L	L	L	L	L	L
Siwiec 1999	USA	26-35wk 810-2750g	Morphine 100mc/kg Later 20mc/kg/hr 1-5 days.	NA	U	U	U	U	U	U	U
Lago 1998	Italy	26-34wk	Fentanyl 0.5-2 mcg/kg/hr	NA	U	U	H	U	L	L	L
**Sedation during ventilation: midazolam *vs.* placebo**											

© 2023 Razak A et al. *JAMA Network Open.*

Anand 1999	Multicenter	24-32w	Midaz 200mc/kg 20,40,60 mc/kg/hr Maintenance.	Dextrose 10%	L	L	L	U	L	U	L
**Sedation during ventilation: phenobarbitone *vs.* placebo**											
No Studies											
**Neuromuscular paralysis during ventilation *vs.* placebo**											
Shaw 1993	UK	<2000g	Pancuronium 80mcg/kg IV, repeat dose if needed.	If restless, morphine IV	U	U	U	U	L	U	L
Perlman 1985	USA	750-1500g	NA	NA	U	U	U	L	L	U	L
**Early erythropoiesis-stimulating agents vs. placebo**											
Juul 2020 (PENUT Trial)	Multicenter	24-28wk	EPO 1000u/kg Q48hrX6, 3/wk till 32+6wk.	Saline	L	L	L	L	L	L	L
Sun 2020	China	24-32wk	EPO 500u/kg AD X2 wks, 7 dose.	Saline	L	U	U	U	L	L	H
Natalucci 2016	Switzerland	26-32wk	EPO 3000u/kgX3, 3hr, 12-18, 36-42hr	Saline	L	U	L	L	L	L	H
Song 2016	China	<32wk	EPO 500u/kg ADx2wk	Saline	L	L	H	L	L	U	L
Fauchere 2015	Multicenter	26-32wk	EPO 3000u/kgX3, 3hr, 12-18, 36-42hr	Saline	L	L	L	L	L	L	L
Peltoniemi 2017	Finland	700-1500g	Eprex 250u/kgX6 for 6 days.	Saline	L	L	L	L	L	L	L
Ohls 2013	Multicenter	500-1250g 24-32wk	EPO 400u/kgx3/ wk, Darb 10mcg/kg SC/wk	Placebo	L	L	L	L	U	L	L
Fauchere 2008	Switzerland	401g-1250g	Epoitin beta 3000u/kg, 3-6, 12-18, 36-42hr	Saline	L	L	L	L	L	U	L
Bierer 2006	USA	<32wk	EPO 400mcgX3/wk upto 35wk	Saline	L	U	L	U	L	U	L
Haiden 2005	Austria	<32wk		No placebo	U	L	H	U	L	U	L

© 2023 Razak A et al. *JAMA Network Open.*

Ohls 2001<br><br>Maier 1994	Multicenter<br><br>Multicenter	401-1250g <32wk<br><br>750-1499g	EPO 300u/kg/d or 700U/kg 3x/wk.<br>EPO 400u/kg 3x wk till 35wk or discharge.<br><br>EPO 250u on AD till 40-42wk, 17doses.	Placebo<br><br>No placebo	U<br><br>U	L<br><br>L	L<br><br>L	L<br><br>L	L<br><br>H	U<br><br>U	L<br><br>L
**Volume expansion *vs.* inotropes (any) for hypotension**											
Gill 1993	UK	<1501g	Dopa 5mcg/kg/min, inc by 2.5mcg /kg/min till 10mcg.	4.5% Alb 20ml/kg over 30min.	L	L	H	U	L	L	L
**Dopamine *vs.* dobutamine for hypotension**											
Hentschel 1995	Germany	25-36wk	Dopa 10mcg/kg/min.	Dobu 10mcg/ kg/min.	L	L	H	L	L	L	L
Klarr 1994	USA	<34wk	Dopa at 5mcg /kg/min, max 20mcg.	Dobu at 5mcg /kg/min, max 20mcg.	L	L	L	L	L	L	L
**Indomethacin prophylaxis for PDA *vs.* placebo**											
Kalani 2016	Iran	600-1250g	Indo 0.2mg/kg OD x 3 days.	Standard care	U	U	U	U	L	L	L
Scmidt 2001	Multicenter	500-999g	Indo 0.1mg/kg OD x3 doses.	Saline	L	L	L	L	L	L	L
Yaseen 1997	KSA	<1750g	Indo 0.2mg/kg OD x3 doses.	Saline	L	L	L	L	L	L	L
Couser 1996	USA	23-29wk	Indo 0.1mg/kg OD x 6 doses.	Saline	L	L	L	L	L	L	L
Ment 1994(a)	Multicenter	600-1250g	Indo 0.1mg/kg,6-8hr, ODx2doses.	Saline	L	L	L	L	L	L	L
Ment 1994(b)	Multicenter	600-1250g	Indo 0.1mg/kg,6-8hr, ODx2doses.	Saline	L	L	L	L	L	L	L
Dominaco 1994	Multicenter	600-1250g	Indo 3 doses, 0.2, 0.1,0.1mg/kg at <12hrs, 24, 36hrs.	Saline	U	L	L	L	L	U	L
Morales Suarez 1994	Mexico	<1250g	Indo 0.1mg/kg Q12hrs.	Saline	U	U	L	L	L	L	L
Bada 1989	USA	28-36wk	Indo 0.2mg/kg,6hr 0.1mg/kg, 18hr, 30hr	Placebo	U	U	L	L	L	L	L

© 2023 Razak A et al. *JAMA Network Open.*

Bandastra 1988	USA	<1500g	Indo 0.2mg/kg, 12hr, 0.1mg/kg Q12hr x 2 doses.	Placebo	L	L	L	L	L	L	L
Hanigan 1988	USA	500-1500g	Indo 0.1mg/kg 12,24,48,72hr.	Saline	L	L	L	L	L	L	L
Ment 1988	USA	500-1500g	Indo 0.1mg/kg Q24hr x 3 doses.	Saline	L	L	L	L	L	L	L
Krueger 1987	USA	600-1250g	Indo 0.2mg/kg 24hr of age.	No Treatment	U	U	H	U	L	L	L
Vincer 1987	Multicenter	750-1500g	Indo 0.2mg/kg, 12hr, Q12hr.	Saline	U	U	L	L	L	L	L
Ment 1985	USA	600-1250g	Indo 0.2mg/kg, 0.1mg/kg Q12hr for 5 doses.	Saline	L	L	L	L	L	L	L
**Indomethacin pre-symptomatic treatment for PDA *vs.* placebo**											
Kluckow 2014	Multicenter	<29wk	Indo 0.2mg/kg, then 0.1mg/kg Q24hr x2 days.	Saline	L	L	L	L	L	L	U
**Ibuprofen prophylaxis for PDA *vs.* placebo**											
Kalani 2016	Iran	<32wk <1500g	Ibup 10,5,5mg/kg Q24h for 3 days.	Standard care	U	U	U	U	L	U	L
Kanmaz 2013	Turkey	<28wk <1000g	Ibup 10mg/kg, 12-24hr, then 5mg/ kg Q24hr, 48hr.	No Treatment	L	L	H	U	L	L	H
Dani 2005	Multicenter	<28wk	Ibup 10mg/kg, 6hrs then 5mg/kg after 24hr, 48hr.	Placebo	U	L	L	U	L	U	L
Gournay 2004	Multicenter	<28wk	Ibup 10/5/5 Q24hr.	Saline	U	L	L	L	L	U	L
Van Overmeire 2004	Multicenter	24-30wk	Ibup10mg/kg, 6hr then 5mg/kg Q24hr, 48hr.	Saline	L	L	L	L	L	L	L
De Carolis 2000	Italy	<31wk >500g	Ibup 10mg/kg, 2 then 5mg/kg Q24hr and 48hrs	No Treatment	U	U	H	U	L	U	L
Dani 2000	Multicenter	<34wk	Ibup 10mg/kg, 24hr, then 5mg/kg at 24hr and 48hr	No Treatment	U	L	H	U	L	U	L
Varvarigou 1996	Canada			Placebo	U	U	L	L	L	L	L

© 2023 Razak A et al. *JAMA Network Open.*

		<32wk <1500g	Ibup10mg/g, 3hr then 5mg/kg at 24hr and 48hr								
**Ibuprofen pre-symptomatic treatment for PDA *vs.* placebo**											
Roze 2020 (TRIOCAPI)	Multicenter	24-27wk	Ibup 10mg/kg, then 5mg/kg 24hr, 48 hrs.	Placebo	L	L	L	L	L	L	L
Sosenko 2012	USA	23-32wk	Ibup 10mg/kg then 5mg/kg Q24hr x 2.	5% Dextrose	L	L	L	L	L	L	L
Aranda 2009	Multicenter	<30wk, 500-1000g	Ibup 10mg/kg then 5mg/kg Q24hr x 2.	Placebo	L	L	L	L	L	L	L
**Restrictive *vs.* liberal packed red cell transfusion for anemia**											
Franz 2020 (ETTNO RCT)	Multicenter	400-999g	PRBC depends on critical and non-critical health. (Hb, HCT-table)	NA	L	L	H	L	L	L	L
Kirpalani 2020 (TOP Trial)	Multicenter	<1000g 22-29wk	PRBC transfusion depends on algorithm.	NA	L	L	H	U	L	L	L
Chen 2009	Taiwan	<1500g	Liberals HCT >45,40,30% (Vent, NIV, Spont breathing) Restrictive HCT kept >35%, 30, 22%.	NA	L	L	H	U	L	U	L
Kirpalani 2006	Multicenter	<31wk <1000g	BT depends upon respiratory support, age and cap/central HCT.	NA	L	L	H	L	L	L	L
Bell 2005	USA	500-1300g	PRBC given according to cap HCT value.	NA	L	L	H	L	H	H	L
Connelly 1999	Canada	<1500g	PRBC according to Hb value at various postnatal week+ resp support	NA	L	L	H	H	L	L	L
**Low *vs.* high threshold for platelet transfusion for thrombocytopenia**											
Curley 2019 (PlaNet 2)	Multicenter	<34wk Plt <50k	Plt transfusion 15ml/kg when Plt <25K.	Plt transfusion 15ml/ kg when Plt <50K.	L	U	H	L	L	L	L
Kumar 2019	India	<35wk			L	L	H	U	L	L	L

© 2023 Razak A et al. *JAMA Network Open.*

		Plt <100k	Plt transfusion to keep >100K.	Plt transfusion 15ml/kg when Plt <50K.							
**Prophylactic plasma administration *vs.* placebo to reduce IVH/PVL**											
NNI Trial group 1996	UK	<32wk	Propylactic plasma infusion 20ml/kg.	10% Dextrose	L	L	H	H	L	U	L
Beverly 1985	UK	<1500g <32wk	FFP 10ml/kg at admission and then at 24hr of age	NA	U	H	H	L	L	U	L
**Prophylactic factor VII administration *vs.* placebo to reduce IVH/PVL**											
NO STUDIES											
**Prophylactic antithrombin III administration vs. placebo to reduce IVH/PVL**											
Fulia 2003	Italy	<30wk Anti III <40%	Anti III loading dose 2 ml/kg (100 U/kg) IV then 1ml/kg (50U/kg) Q8hr.	5% Dextrose	L	H	U	U	L	U	L
Schmidt 1998	Canada	750-1900g	Anti III loading dose 2 ml/kg (100 U/kg) 1ml/kg (50U/kg) Q6hr, 48hr.	1% Albumin	L	L	L	L	L	L	U
**Prophylactic ethamsylate administration vs. placebo to reduce IVH/PVL**											
Sanghvi 1999	India	<34wk	Etham 12.5 mg at1 hr then Q6hr X 4 days.	Routine therapy	L	L	U	L	H	L	L
EC Trial group 1994	Multicenter	<32wk <4hr	Etham12.5mg/k then 15 dose Q6hr x 4 days.	Placebo	L	H	L	L	U	L	L
Chen 1993	Taiwan	<1751g	Etham 12.5mg/kg, 1 hr, then Q6hr for 4 days.	Saline	U	U	L	L	L	L	L
Amato 1992	Switzerland	Pre-term	Etham 12.5 mg/kg 4hr of birth, then Q6hr x 4 days.	Saline	U	U	U	U	L	L	L
Benson 1986	UK	<1500g	Etham 12.5 mg/kg 1 hr of birth, then Q6hr for 4 days.	Placebo	L	L	L	L	L	L	L
Morgan 1981	UK	<1500g	Etham 12.5 mg/kg 2 hr of birth, then Q6hr for 4 days.	Placebo	L	L	L	L	L	L	L

© 2023 Razak A et al. *JAMA Network Open.*

**Prophylactic heparin administration vs. placebo to reduce IVH/PVL**											
Chang 1997	USA	<31wk	Heparin 1U/ml through UVC.	No Heparin	U	U	L	L	L	U	U
**Stem cell therapy**											
No RCT											
**Vitamin A supplementation *vs.* placebo for the outcome severe IVH**											
Sun 2020	China	<28 days <96hr	Vit A 1500IU/day, until 28 days or discharge, IM.	Placebo	U	U	L	L	L	L	L
Kiatchoosakun 2014	Thailand	<1500g	Vit A 5000IU Mon, Wed, Fri for 4wk IM.	Sham Procedure	L	L	L	U	L	U	L
Mactier 2012	Glasgow	<1501g <32wk	Vit A 10,000 IU IM x 3/wk Max 12 doses.	NA	L	L	L	L	L	L	L
Ravishankar 2003	USA	<32wk 500-1500G	Vit A 50,000 IU/mL, 1, 3 and 7 days, IM.	Sham Procedure	U	L	L	L	L	U	L
Tyson 1999	Multicenter	401-1000g	Vit A 5000IU Mon, Wed, Fri for 4wk, IM.	Sham Procedure	L	L	L	L	L	L	L
Bental 1994	SA	1000-1500g, <=34wk	Vit A 4000 units IM x 3/wk total 12 doses.	NA	L	U	U	U	L	L	U
Papagaroufalis 1991	Greece	25-29wk 560-1240g	Vit A 5000U IM AD X 8 dose over 15days.	NS	U	U	H	U	L	U	L
**Vitamin E supplementation *vs.* placebo for the outcome severe IVH**											
Barekatein 2018	Iran	<30wk	10U of Vit E oral x3 days.	Distilled water	U	U	U	U	L	L	L
Fish 1990	USA	<1000g	Vit E IM 15mg, then 10mg OD 1,2,4,6 days.	Placebo	L	L	L	L	H	U	L
Phleps 1987	USA			Placebo	L	L	L	L	L	U	L

© 2023 Razak A et al. *JAMA Network Open.*

Sinha 1987	UK	<1500g <33wk	Vit E 20mg/kg IV repeated on D2, D3, Cont'd till retinal exam.	No Placebo	L	L	H	U	L	U	L
Hittner 1984	USA	≤32wk	Vit E 20mg/kg IM D0, then Q24hr and 48hr.	Placebo	L	L	L	L	L	L	L
Speer 1984	USA	<15000g +RDS	Vit E 100mg/kg 24hr of age and then IM injection at 15, 10, 10, and 10 mg/kg on days 1,2,4 and 6.	Placebo	U	U	U	L	L	U	L
Hittner 1981	USA	<1500g,	Vit E 100mg/kg via NGT, IM D1,2,4,6.	Placebo	L	L	L	L	L	L	L
		<1500g,	Vit E 100 mg/kg/day till stay.								
**Vitamin K supplementation *vs.* placebo for the outcome severe IVH**											
No RCT											

NOTE: AD: Alternate days, **Alb:** Albumin, **Amp:** Amplitude, **Anti III:** Antithrombin III, **Beta:** Betamethasone, **Caff:** Caffeine, **Cap:** capillary, **cm**: centimeter, **CPAP:** Continuous Positive Airway Pressure, **CV**: Conventional ventilation, **DCC:** Delayed Cord Clamping, **Dexa:** Dexamethasone, **Dobu:** Dobutamine, **Dopa:** Dopamine, **ECC:** Early Cord Clamping, **EPO:** Erythropoietin, **Etham:** Ethamsylate, **ETT:** Endotracheal Tube, **FFP:** Fresh frozen plasma. **Fr**: French, **G**: Gauge: **g**: gram, **H:** High, **Hb:** Hemoglobin, **HCT:** Hematocrit, **HFOV:** High Frequency Oscillation Ventilation, **Hz:** Hertz, **hr**: Hour, **H2O**: Water, **Ibup:** Ibuprofen, **IM:** Intramuscular, **IMV:** Intermittent mandatory ventilation, **Indo:** Indomethacin, **IPPV:** Intermittent Positive Pressure Ventilation, **IVH:** Intraventricular Hemorrhage, **IU:** International Unit, **IV**: Intravenous, **K**: Thousand, **Kg**: kilogram, **KSA:** kingdom of Saudi Arabia, **L:** Loading dose, **M:** Maintenance dose, **MAP:** Mean Airway Pressure, **L**: Low **MISA:** Minimally invasive surfactant adminstration**, Midaz:** Midazolam, **MgSO4:** Magnesium Sulphate, **NA:** Not Available, **NGT**: Nasogastric tube, **NIV:** Non-invasive ventilation, **NS:** Normal Saline, **OD**: Once Daily, **PC:** Pressure Control**, PEEP:** Positive End Expiratory Pressure, **PIP:** Peak Inspiratory Pressure, **PPV:** Positive Pressure Ventilation, **Plt:** Platelet, **PLV:** Pressure-limited ventilation**, PaCO2:**Partial pressure of carbon dioxide, **PRVC:** Pressure regulated volume control, **PRBC:** Packed Red blood Cell, **PSV:** Pressure support ventilation, **PVL:** Peri-ventricular leukomalecia, **Q (12,24,36,48)**: Every (12,24,36,48), **SIPPV:** Synchronous intermittent positive pressure ventilation, **SIMV:** Synchronized intermittent mandatory ventilation, **SA**: South Africa, **SC**: Subcutaneous, **Sec:** Seconds, **SLI:** Sustained Lung inflation, **SpO2**: Oxygen saturation, **U**: Uncertain, **UVC:** Umbilical

© 2023 Razak A et al. *JAMA Network Open.*

Venous Catheter, **UCM:** Umbilical Cord Milking, **USA:** United States of America**, UK:** United Kingdom, **VG:** Volume Guarantee, **Vit A:** Vitamin A, **Vit E:** Vitamin E, **Vit K:** Vitamin K, **VT:** Tidal Volume, **Wk:** Week, **X**: times.

© 2023 Razak A et al. *JAMA Network Open.*

e-Table 2: Summary of meta-analysis of interventions on cystic periventricular leukomalacia and severe brain injury.

			RR (95% CI)	Heterogeneity (I^2)	GRADE
CYSTIC PVL					
**Antenatal Interventions**					
Antenatal corticosteroids for lung maturity	01	326	5.25(0.25, 108.51)	NA	Low[3]
Betamethasone ***vs.*** Dexamethasone for lung maturity	01	157	0.47 (0.04, 5.07)	NA	Low[1, 3]
Repeat antenatal steroids ***vs.*** no repeat antenatal steroids	03	3113	0.98 (0.46, 2.07)	00	Moderate[1]
Magnesium sulfate for neuroprotection or tocolysis	02	1900	0.97 (0.66, 1.42)	00	High
**Delivery Room Interventions**					
Lower vs. higher FiO2 for resuscitation at birth	02	157	0.33 (0.01, 7.97)	NA	Low[1, 3]
Sustained inflation ***vs.*** standard resuscitation	05	635	0.59 (0.24, 1.44)	00	Low[1, 3] (
Umbilical cord milking ***vs.*** immediate cord clamping	01	40	0.50 (0.05, 5.08)	NA	Very low[1, 3]
**Postnatal Interventions**					
Supine head midline vs Supine head rotated	02	228	3.25(0.14, 76.01)	NA	Low[1, 3]
LISA ***vs.*** INSURE	02	431	0.69 (0.30, 1.57)	75	Very low[1, 2, 3]
Volume guarantee ***vs.*** pressure limited ventilation	01	40	3.0 (0.13, 69.52)	NA	Low[1, 3]
Elective HFOV ***vs.*** conventional ventilation	04	1365	0.76 (0.49, 1.16)	00	Low[1, 3]
Elective HFJV ***vs.*** conventional ventilation	01	64	5.0 (1.19, 21.04)	NA	Low[1, 3]
Oxygen saturation target after birth: 85-89% ***vs.*** 91-95%	01	1272	1.23(0.73, 2.08)	NA	High
Permissive hypercapnia ***vs.*** normocapnia	01	49	1.04 (0.16, 6.81)	NA	Low[1, 3]
Caffeine prophylaxis or treatment for apnea or post-extubation	01	2016	0.65 (0.39, 1.07)	NA	High
High dose ***vs.*** low dose caffeine for apnea or post-extubation	01	246	0.48 (0.17, 1.33)	00	Low[1, 3]
Sedation during ventilation: opioids ***vs.*** no opioids	01	734	0.79 (0.49, 1.29)	NA	Moderate[3]
Neuromuscular paralysis during ventilation	01	193	1.26 (0.35, 4.56)	NA	Low[1, 3]
Early erythropoiesis-stimulating agents	03	1551	0.59 (0.42, 0.83)	00	Low[1]
Indomethacin prophylaxis for PDA	02	289	0.36 (0.12, 1.06)	00	Moderate[3]
Indomethacin pre-symptomatic treatment for PDA	01	92	1.09 (0.29, 4.10)	NA	Low[1, 3]
Ibuprofen prophylaxis for PDA	03	701	1.21 (0.63, 2.30)	00	Moderate[1]
Restrictive ***vs.*** liberal packed cell transfusion for anemia	03	1558	0.91 (0.56, 1.47)	34	Moderate[1]
SEVERE BRAIN INJURY					

© 2023 Razak A et al. *JAMA Network Open.*

**Antenatal Interventions**					
Antenatal corticosteroids for lung maturity	01	1530	1.78 (0.45,7.08)	NA	High
**Delivery Room Interventions**					
Delayed cord clamping ***vs.*** immediate cord clamping	02	2905	1.16 (0.80,1.69)	00	Moderate[1]
**Postnatal Interventions**					
Elective HFOV *vs.* conventional ventilation	04	1769	0.79 (0.63, 0.99)	00	Low[1, 3]
Oxygen saturation target after birth: 85-89% *vs.* 91-95%	04	3348	0.94 (0.80, 1.10)	16	High
Caffeine prophylaxis or treatment for apnea or post-extubation	01	1933	0.91 (0.73, 1.14)	NA	High
Indomethacin prophylaxis for PDA	02	1215	0.85 (0.70, 1.05)	04	Moderate[3]
Restrictive *vs.* liberal packed cell transfusion for anemia	03	2248	0.94 (0.78, 1.13)	40	Moderate[1]

Note: **CI**: confidence interval; **FiO2**: fraction of inspired oxygen; **GRADE**: Grading of Recommendations Assessment, Development and Evaluation—[1]Risk of bias, [2]heterogeneity, [3]imprecision, [4]publication bias, [5]inconsistency; **HFJV**: high-frequency oscillatory ventilation; **HFOV**: high-frequency oscillatory ventilation; **INSURE**: Intubate-surfactant-extubate; **IVH**: intraventricular hemorrhage; **LISA**: less-invasive surfactant treatment; **NA**: not available; **PDA**: patent ductus arteriosus; **PVL**: periventricular leukomalacia; **RCTs**: randomized clinical trials; **RR**: relative risk, **vs**.: Versus.

© 2023 Razak A et al. *JAMA Network Open.*

e-Table 3: Summary of sensitivity and subgroup analysis of interventions on severe intraventricular hemorrhage and cystic periventricular leukomalacia

Antenatal corticosteroids for lung maturity	NP	NP	NP	NP
Dexamethasone ***vs.*** Betamethasone for lung maturity	NP	NP	NP	NP
Repeat antenatal steroids ***vs.*** no repeat antenatal steroids	NP	NP	NP	NP
Magnesium sulfate for tocolysis or neuroprotection	NP	NP	NP	NP
Antibiotics for premature rupture of membranes	NP	NP	NP	NP
Cesarean section ***vs.*** vaginal delivery for preterm birth	NP	NP	NP	NP
Lower vs. higher FiO2 for resuscitation	NP	NP	NP	NP
Sustained inflation ***vs.*** standard resuscitation	NP	NP	NP	NP
Delayed cord clamping ***vs.*** immediate cord clamping	NP	NP	NP	NP
Umbilical cord milking ***vs.*** immediate cord clamping	NP	NP	NP	NP
Umbilical cord milking ***vs.*** delayed cord clamping	NP	NP	NP	NP
Delayed cord clamping with respiratory support ***vs.*** delayed cord clamping without respiratory support	NP	NP	NP	NP
Head midline position	NP	NP	NP	NP
LISA ***vs.*** INSURE	NP	NP	No high-quality studies	Subgroup data not available
Volume guarantee ***vs.*** conventional ventilation	NP	NP	NP	NP
Elective HFOV ***vs.*** conventional ventilation	NP	NP	NP	NP
Elective HFJV ***vs.*** conventional ventilation	NP	NP	NP	NP
Oxygen saturation target after birth: 85-89% ***vs.*** 91-95%	NP	NP	NP	NP
Permissive hypercapnia ***vs.*** normocapnia	NP	NP	NP	NP
Early extubation ***vs.*** delayed extubation	NP	NP	NP	NP
Caffeine prophylaxis or treatment for apnea or post-extubation	0.86 (0.67,1.11), I^2=NA n=1933, 1 trial (GRADE)	Subgroup data not available	NP	NP

© 2023 Razak A et al. *JAMA Network Open.*

High dose ***vs.*** low dose caffeine for apnea or post-extubation	NP	NP	NP	NP
Sedation during ventilation: midazolam ***vs.*** no midazolam	NP	NP	NP	NP
Sedation during ventilation: opioids ***vs.*** no opioids	NP	NP	NP	NP
Sedation during ventilation: phenobarbitone ***vs.*** no phenobarbitone	NP	NP	NP	NP
Neuromuscular paralysis during ventilation	No high-quality studies	Subgroup data not available	NP	NP
Early erythropoiesis-stimulating agents	0.99 (0.76. 1.31), I^2=42%, n=1372, 6 trials (GRADE-low)	Subgroup data not available	NP	NP
Volume expansion ***vs.*** inotropes (any) for hypotension	NP	NP	NP	NP
Dopamine ***vs.*** dobutamine for hypotension	NP	NP	NP	NP
Indomethacin prophylaxis for PDA	NP	NP	NP	NP
Indomethacin pre-symptomatic treatment for PDA	NP	NP	NP	NP
Ibuprofen prophylaxis for PDA	NP	NP	NP	NP
Ibuprofen pre-symptomatic treatment for PDA	NP	NP	NP	NP
Restrictive ***vs.*** liberal packed cell transfusion for anemia	NP	NP	NP	NP
Low ***vs.*** high threshold for platelet transfusion for thrombocytopenia	NP	NP	NP	NP
Prophylactic plasma administration to reduce IVH/PVL	NP	NP	NP	NP
Prophylactic factor VII administration to reduce IVH/PVL	NP	NP	NP	NP
Prophylactic antithrombin III administration to reduce IVH/PVL	NP	NP	NP	NP
Prophylactic ethamsylate administration to reduce IVH/PVL	NP	NP	NP	NP
Prophylactic heparin administration to reduce IVH/PVL	NP	NP	NP	NP
Stem cell therapy	NP	NP	NP	NP
Vitamin A supplementation	NP	NP	NP	NP

© 2023 Razak A et al. *JAMA Network Open.*

Vitamin E supplementation	NP	NP	NP	NP
Vitamin K supplementation	NP	NP	NP	NP

Note: **CI**: confidence interval; **FiO2**: fraction of inspired oxygen; **GRADE**: Grading of Recommendations Assessment, Development and Evaluation—[1]Risk of bias, [2]heterogeneity, [3]imprecision, [4]publication bias, [5]inconsistency; **HFJV**: high-frequency oscillatory ventilation; **HFOV**: high-frequency oscillatory ventilation; **INSURE**: Intubate-surfactant-extubate; **IVH**: intraventricular hemorrhage; **LISA**: less-invasive surfactant treatment; **NA**: not available; **NP**: not performed (no significant heterogeneity in the main meta-analysis); **PDA**: patent ductus arteriosus; **PVL**: periventricular leukomalacia; **RCTs**: randomised clinical trials; **RR**: relative risk, **vs.**: Versus, **NP**: ??

© 2023 Razak A et al. *JAMA Network Open.*

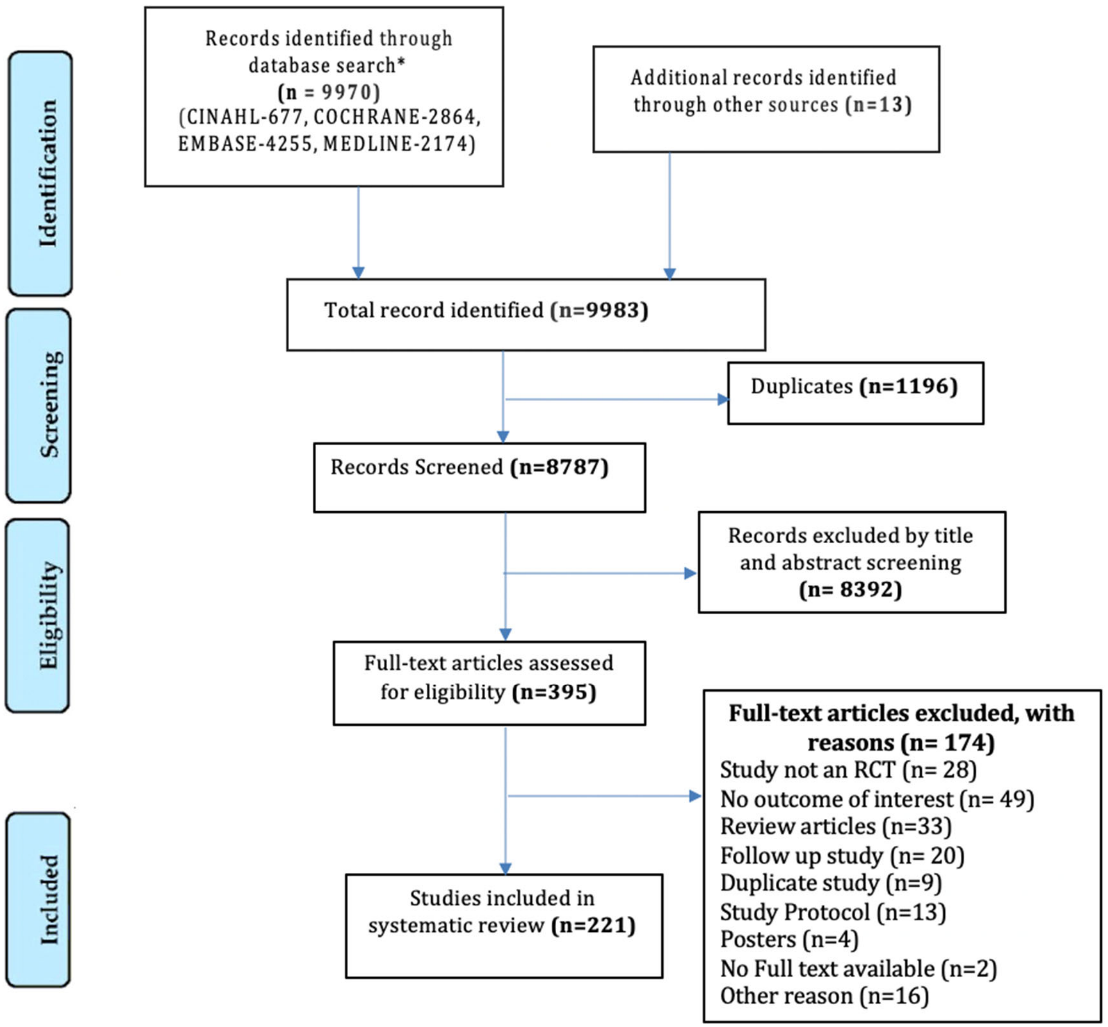

eFigure 1. Study Flow diagram outlining stages of search results and filtering process [as per Preferred Reporting Items for Systematic Reviews and Meta-Analyses (PRISMA) guidelines]

© 2023 Razak A et al. *JAMA Network Open.*

Study or Subgroup	Betamethasone Events	Betamethasone Total	Dexamethasone Events	Dexamethasone Total	Weight	Risk Ratio M-H, Fixed, 95% CI
Chen 2005	3	81	2	76	29.5%	1.41 [0.24, 8.19]
Crowther 2019	5	737	3	752	42.5%	1.70 [0.41, 7.09]
Elimian 2007	7	100	2	105	27.9%	3.67 [0.78, 17.27]
Subtil 2003	0	69	0	36		Not estimable
**Total (95% CI)**		**987**		**969**	**100.0%**	**2.17 [0.89, 5.25]**
Total events	15		7			

Heterogeneity: $Chi^2 = 0.79$, df = 2 (P = 0.67); $I^2 = 0\%$
Test for overall effect: Z = 1.71 (P = 0.09)

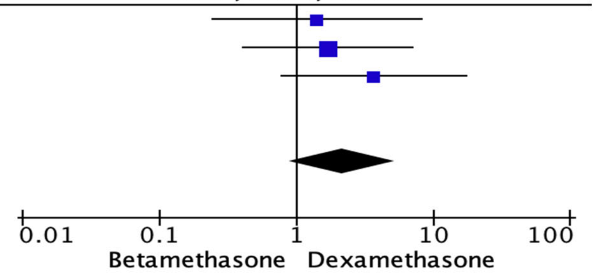

eFigure 2. Forest plot for comparison—Betamethasone for lung maturity vs Dexamethasone for lung maturity for outcome severe IVH.

Study or Subgroup	Repeat Course Events	Repeat Course Total	Single Course Events	Single Course Total	Weight	Risk Ratio M-H, Fixed, 95% CI
Asghajafari 2002	0	9	1	7	3.4%	0.27 [0.01, 5.70]
Crowther 2006	5	567	8	577	16.2%	0.64 [0.21, 1.93]
Garite 2009	6	272	4	274	8.1%	1.51 [0.43, 5.30]
Guinn 2001	9	248	2	235	4.2%	4.26 [0.93, 19.53]
Lee 2004	5	81	2	80	4.1%	2.47 [0.49, 12.36]
Murphy 2008	6	1164	9	1140	18.6%	0.65 [0.23, 1.83]
Peltoniemi 2007	6	159	4	167	8.0%	1.58 [0.45, 5.48]
Wapner 2006	15	250	18	242	37.4%	0.81 [0.42, 1.56]
**Total (95% CI)**		**2750**		**2722**	**100.0%**	**1.06 [0.73, 1.56]**
Total events	52		48			

Heterogeneity: $Chi^2 = 8.07$, df = 7 (P = 0.33); $I^2 = 13\%$
Test for overall effect: Z = 0.32 (P = 0.75)

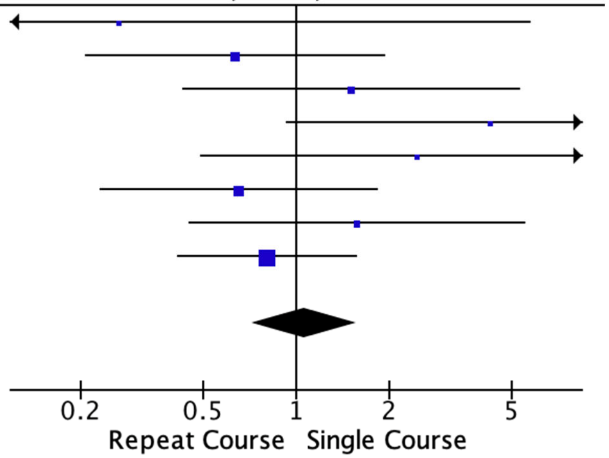

eFigure 3. Forest plot for comparison—Antenatal steroid for lung maturity: repeat course vs single course for outcome severe IVH.

© 2023 Razak A et al. *JAMA Network Open.*

Study or Subgroup	MgSO4 Events	MgSO4 Total	Placebo Events	Placebo Total	Weight	Risk Ratio M-H, Fixed, 95% CI
**1.1.1 MgSO4 for Neuroprotection**						
Crowther 2003	49	620	50	615	48.2%	0.97 [0.67, 1.42]
Marret 2006	11	341	13	324	12.8%	0.80 [0.37, 1.77]
Parashi 2017	0	60	0	60		Not estimable
Rouse 2008	23	1112	38	1184	35.3%	0.64 [0.39, 1.07]
**Subtotal (95% CI)**		**2133**		**2183**	**96.3%**	**0.83 [0.63, 1.10]**
Total events	83		101			
Heterogeneity: Chi² = 1.62, df = 2 (P = 0.45); I² = 0%						
Test for overall effect: Z = 1.30 (P = 0.19)						
**1.1.2 MgSO4 for Tocolysis**						
Fox 1993	0	45	0	45		Not estimable
How 1998	0	84	3	69	3.7%	0.12 [0.01, 2.24]
**Subtotal (95% CI)**		**129**		**114**	**3.7%**	**0.12 [0.01, 2.24]**
Total events	0		3			
Heterogeneity: Not applicable						
Test for overall effect: Z = 1.42 (P = 0.15)						
**Total (95% CI)**		**2262**		**2297**	**100.0%**	**0.80 [0.61, 1.06]**
Total events	83		104			
Heterogeneity: Chi² = 3.33, df = 3 (P = 0.34); I² = 10%						
Test for overall effect: Z = 1.53 (P = 0.12)						
Test for subgroup differences: Chi² = 1.67, df = 1 (P = 0.20), I² = 40.2%						

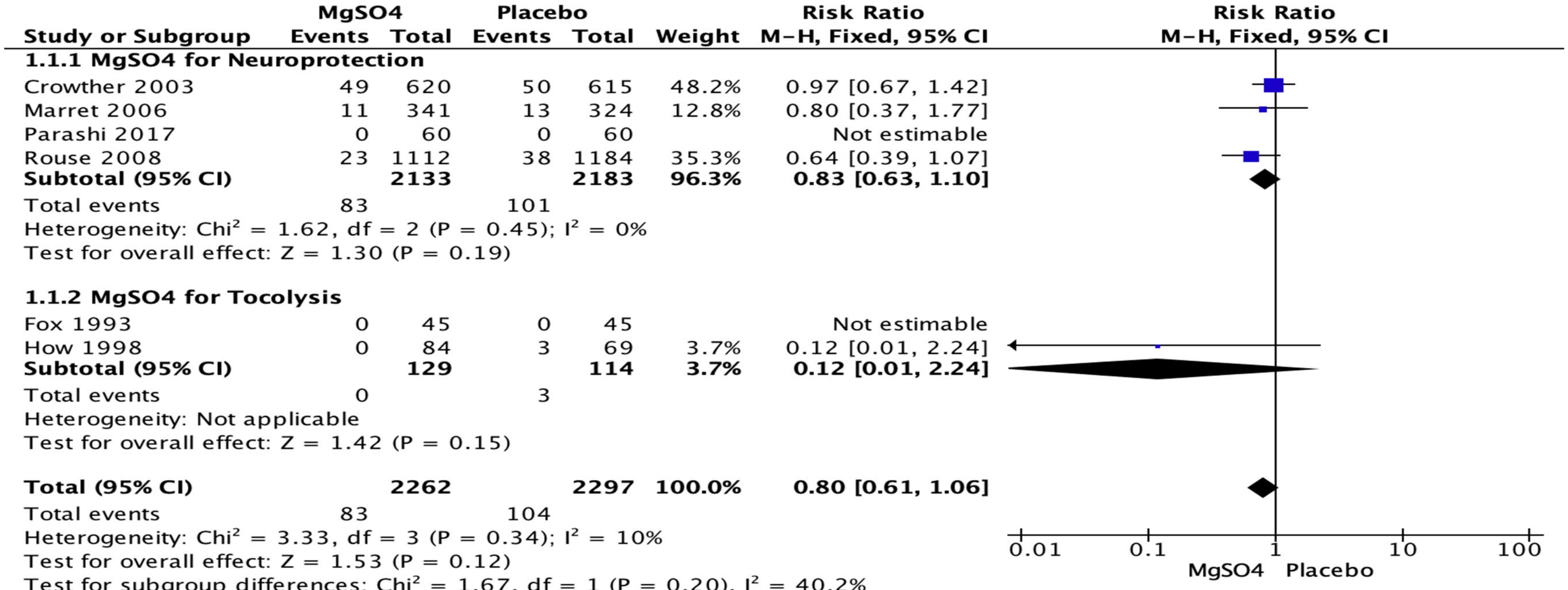

eFigure 4. Forest plot for comparison—Antenatal MgSO4 therapy for Neuroprotection or Tocolysis vs placebo for the outcome severe IVH.

© 2023 Razak A et al. *JAMA Network Open.*

Study or Subgroup	Antibiotics for PROM Events	Antibiotics for PROM Total	Placebo Events	Placebo Total	Weight	Risk Ratio M-H, Fixed, 95% CI
Fuhr 2006	0	47	2	58	7.8%	0.25 [0.01, 5.00]
Grable 1996	0	31	0	29		Not estimable
Mercer 1997	19	299	24	312	81.7%	0.83 [0.46, 1.48]
Owen 1993	1	59	3	58	10.5%	0.33 [0.04, 3.06]
**Total (95% CI)**		**436**		**457**	**100.0%**	**0.73 [0.42, 1.26]**
Total events	20		29			

Heterogeneity: $Chi^2 = 1.17$, df = 2 (P = 0.56); $I^2 = 0\%$

Test for overall effect: Z = 1.14 (P = 0.26)

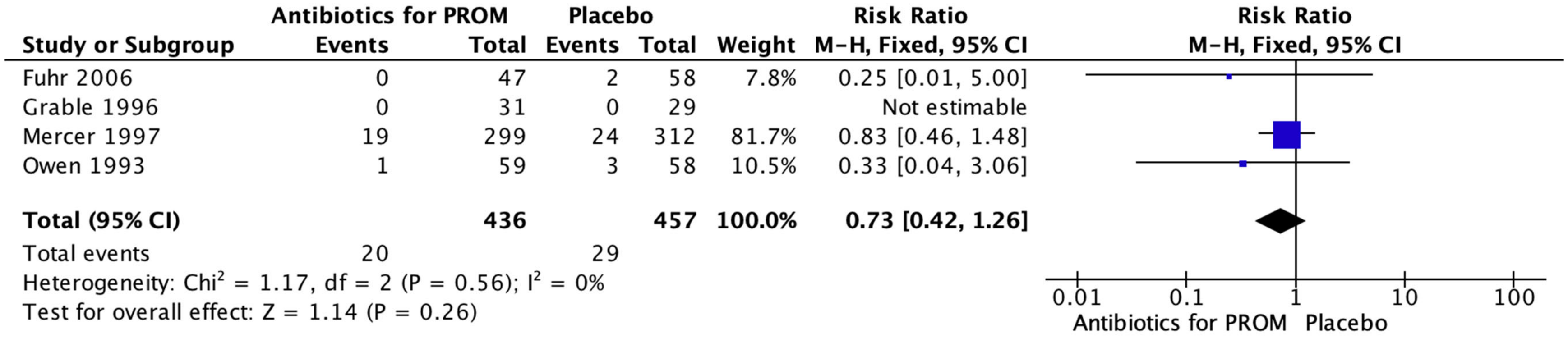

eFigure 5. Forest plot for comparison—Antibiotics for PPROM vs placebo for the outcome severe IVH.

© 2023 Razak A et al. *JAMA Network Open.*

Study or Subgroup	Cord Milking Events	Cord Milking Total	DCC Events	DCC Total	Weight	Risk Ratio M-H, Fixed, 95% CI
Bichkar 2019	1	25	2	24	11.7%	0.48 [0.05, 4.95]
Finn 2019	1	18	0	14	3.2%	2.37 [0.10, 54.08]
Katheria 2015	3	98	5	99	28.6%	0.61 [0.15, 2.47]
Katheria 2019	20	236	8	238	45.7%	2.52 [1.13, 5.61]
Katheria 2020	5	27	0	29	2.8%	11.79 [0.68, 203.52]
Rabe 2011	0	27	1	31	8.0%	0.38 [0.02, 8.98]
**Total (95% CI)**		**431**		**435**	**100.0%**	**1.82 [1.03, 3.21]**
Total events	30		16			

Heterogeneity: $Chi^2 = 6.86$, df = 5 (P = 0.23); $I^2 = 27\%$
Test for overall effect: Z = 2.05 (P = 0.04)

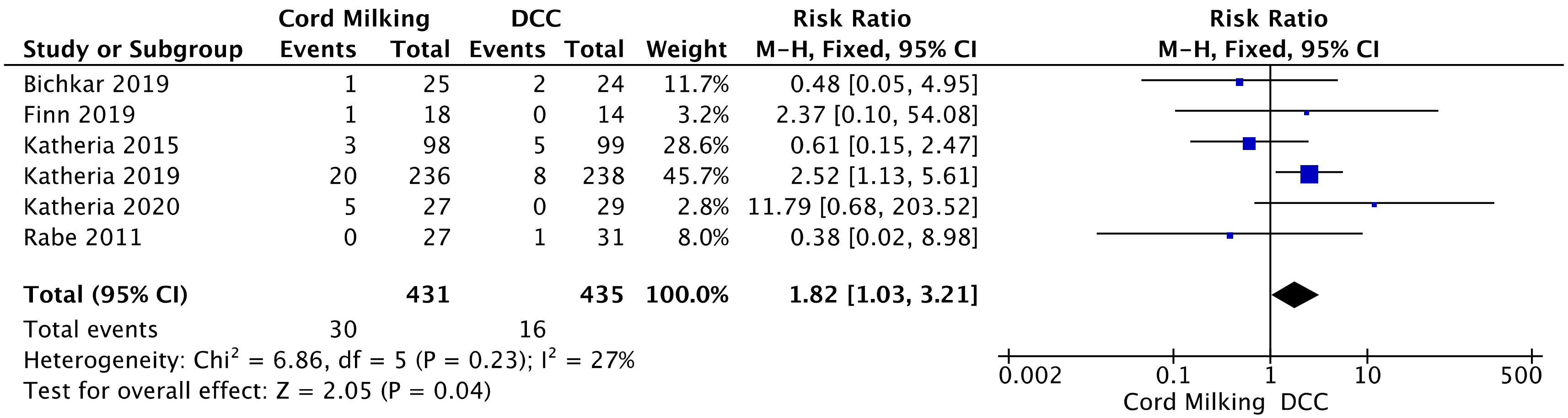

eFigure 6. Forest plot for umbilical cord milking vs. delayed cord clamping in preterm infants for the outcome severe IVH.

Study or Subgroup	Fio2 < 0.4 Events	Fio2 < 0.4 Total	Fio2 > 0.4 Events	Fio2 > 0.4 Total	Weight	Risk Ratio M-H, Fixed, 95% CI
Aguar 2013	3	34	3	26	8.3%	0.76 [0.17, 3.48]
Boronat 2013	19	133	18	120	46.0%	0.95 [0.52, 1.73]
Escrig 2008	2	19	4	23	8.8%	0.61 [0.12, 2.95]
Kapadia 2013	1	44	1	44	2.4%	1.00 [0.06, 15.49]
Lundstrom 1995	2	34	3	35	7.2%	0.69 [0.12, 3.85]
Oei 2017	2	144	6	143	14.6%	0.33 [0.07, 1.61]
Vento 2009	7	37	5	41	11.5%	1.55 [0.54, 4.47]
Wang 2008	2	18	0	23	1.1%	6.32 [0.32, 123.86]
**Total (95% CI)**		**463**		**455**	**100.0%**	**0.92 [0.61, 1.40]**
Total events	38		40			

Heterogeneity: $Chi^2 = 4.60$, df = 7 (P = 0.71); $I^2 = 0\%$
Test for overall effect: Z = 0.37 (P = 0.71)

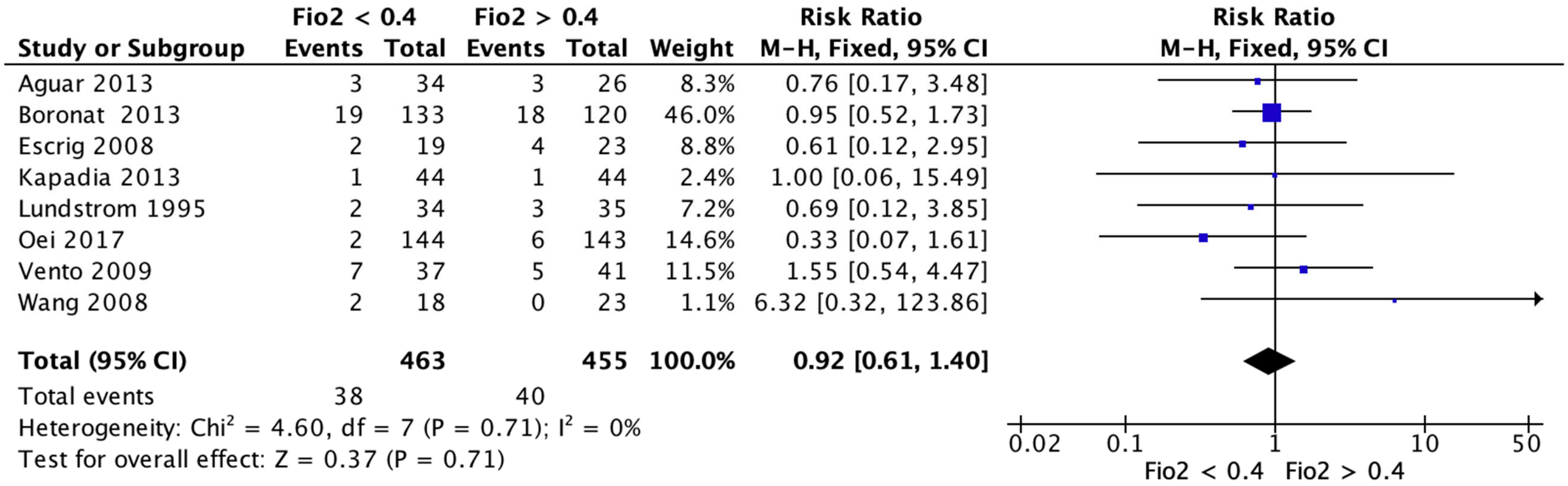

eFigure 7. Forest plot for comparison—lower FiO2 for resuscitation vs. higher FiO2 at resuscitation for the outcome severe IVH.

© 2023 Razak A et al. *JAMA Network Open.*

Study or Subgroup	Sustained Inflation Events	Sustained Inflation Total	Standard resuscitation Events	Standard resuscitation Total	Weight	Risk Ratio M-H, Fixed, 95% CI
El fattah 2017	0	80	1	20	3.5%	0.09 [0.00, 2.05]
Hamd 2020	7	40	3	36	4.6%	2.10 [0.59, 7.52]
Jiravisitkul 2017	0	43	2	38	3.9%	0.18 [0.01, 3.58]
Kirpalani 2019	21	215	22	211	32.6%	0.94 [0.53, 1.65]
Laverde 2019	1	22	1	22	1.5%	1.00 [0.07, 15.00]
Linder 2005	3	31	2	30	3.0%	1.45 [0.26, 8.09]
Lista 2015	12	148	8	143	11.9%	1.45 [0.61, 3.44]
Nagan 2017	17	76	26	86	35.8%	0.74 [0.44, 1.25]
Schmolzer 2018	1	5	2	4	3.3%	0.40 [0.05, 2.98]
Schwaberger 2015	0	20	0	20		Not estimable
**Total (95% CI)**		**680**		**610**	**100.0%**	**0.92 [0.67, 1.26]**
Total events	62		67			

Heterogeneity: $Chi^2 = 7.56$, df = 8 (P = 0.48); $I^2 = 0\%$
Test for overall effect: Z = 0.51 (P = 0.61)

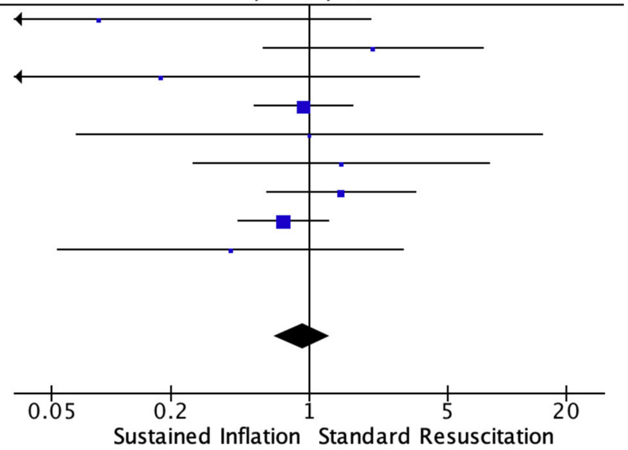

eFigure 8. Forest plot for comparison—Sustained inflation during resuscitation vs. standard resuscitation for the outcome severe IVH.

Study or Subgroup	DCC Events	DCC Total	ECC Events	ECC Total	Weight	Risk Ratio M-H, Fixed, 95% CI
Armanian 2017	1	30	0	30	1.0%	3.00 [0.13, 70.83]
Backes 2016	1	17	4	20	7.5%	0.29 [0.04, 2.39]
Dong 2016	8	46	5	44	10.5%	1.53 [0.54, 4.32]
Duley L 2018	6	134	7	132	14.4%	0.84 [0.29, 2.45]
Finn 2019	0	14	2	12	5.5%	0.17 [0.01, 3.29]
Hemmati 2020	1	69	4	79	7.6%	0.29 [0.03, 2.50]
Hofmeyr 1988	2	23	0	13	1.3%	2.92 [0.15, 56.51]
Hofmeyr 1993	1	40	2	46	3.8%	0.57 [0.05, 6.11]
Kelly 2021	0	19	1	19	3.1%	0.33 [0.01, 7.70]
Kugelman 2007	0	30	1	35	2.8%	0.39 [0.02, 9.16]
Mercer 2003	0	16	0	16		Not estimable
Mercer 2006	0	36	1	36	3.1%	0.33 [0.01, 7.92]
Rabe 2000	0	19	0	20		Not estimable
Tarnow Mordi 2017	24	734	17	712	35.3%	1.37 [0.74, 2.53]
Yunis 2020	1	30	2	30	4.1%	0.50 [0.05, 5.22]
**Total (95% CI)**		**1257**		**1244**	**100.0%**	**0.96 [0.65, 1.42]**
Total events	45		46			

Heterogeneity: $Chi^2 = 8.54$, df = 12 (P = 0.74); $I^2 = 0\%$
Test for overall effect: Z = 0.20 (P = 0.84)

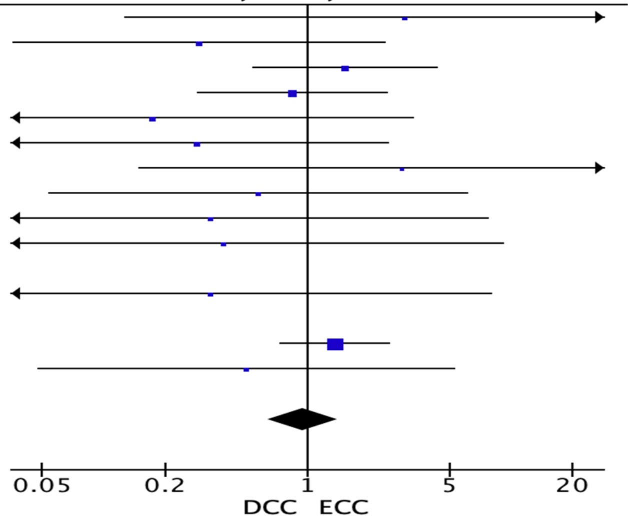

eFigure 9. Forest plot for comparison—Delayed cord clamping vs. early cord clamping for the outcome severe IVH.

© 2023 Razak A et al. *JAMA Network Open.*

Study or Subgroup	Cord Milking Events	Cord Milking Total	ECC Events	ECC Total	Weight	Risk Ratio M-H, Fixed, 95% CI
Alan 2014	3	22	0	22	1.2%	7.00 [0.38, 128.02]
Elimian 2014	3	99	3	101	7.2%	1.02 [0.21, 4.93]
El Naggar 2020	5	37	5	36	12.2%	0.97 [0.31, 3.08]
Finn 2019	1	18	2	12	5.8%	0.33 [0.03, 3.28]
Hosono 2008	2	20	4	20	9.7%	0.50 [0.10, 2.43]
Josephson 2020	2	29	3	27	7.5%	0.62 [0.11, 3.43]
Juan 2018	1	48	3	54	6.8%	0.38 [0.04, 3.49]
Katheria 2014	2	30	4	30	9.7%	0.50 [0.10, 2.53]
March 2013	12	36	12	39	27.8%	1.08 [0.56, 2.10]
Mercer 2016	3	100	3	99	7.3%	0.99 [0.20, 4.79]
Rammohan 2018	0	30	1	30	3.6%	0.33 [0.01, 7.87]
Song 2017	2	32	0	34	1.2%	5.30 [0.26, 106.40]
**Total (95% CI)**		**501**		**504**	**100.0%**	**0.91 [0.61, 1.37]**
Total events	36		40			

Heterogeneity: Chi^2 = 6.54, df = 11 (P = 0.84); I^2 = 0%
Test for overall effect: Z = 0.44 (P = 0.66)

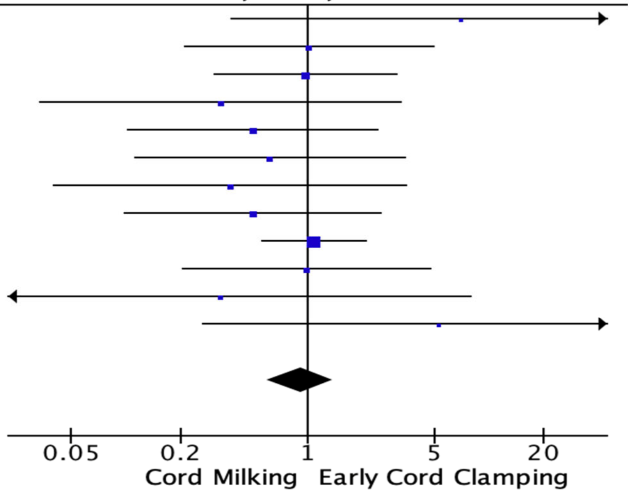

eFigure 10. Forest plot for comparison—Umbilical cord milking vs. early cord clamping for the outcome severe IVH.

Study or Subgroup	DCC with Resp Support Events	DCC with Resp Support Total	DCC alone Events	DCC alone Total	Weight	Risk Ratio M-H, Fixed, 95% CI
Katheria 2016	4	75	3	75	100.0%	1.33 [0.31, 5.75]
**Total (95% CI)**		**75**		**75**	**100.0%**	**1.33 [0.31, 5.75]**
Total events	4		3			

Heterogeneity: Not applicable
Test for overall effect: Z = 0.39 (P = 0.70)

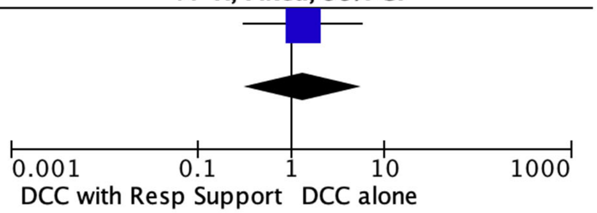

eFigure 11. Forest plot for comparison—Delayed cord clamping with respiratory support vs. delayed cord clamping without respiratory support for the outcome severe IVH.

© 2023 Razak A et al. *JAMA Network Open.*

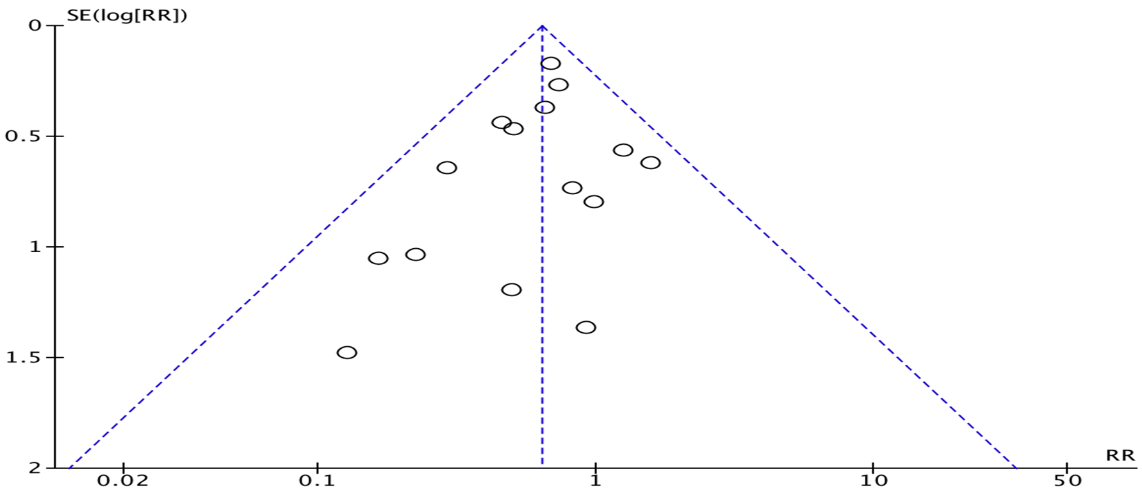

eFigure 12. Funnel plot of Indomethacin prophylaxis for patent ductus arteriosus vs. placebo for outcome severe IVH.

Egger's regression intercept

Intercept	-0.44728
Standard error	0.38610
95% lower limit (2-tailed)	-1.28140
95% upper limit (2-tailed)	0.38685
t-value	1.15845
df	13.00000
P-value (1-tailed)	0.13376
P-value (2-tailed)	0.26752

eFigure 13. Egger's regression test for assessing publication bias for indomethacin prophylaxis for patent ductus arteriosus

© 2023 Razak A et al. *JAMA Network Open.*

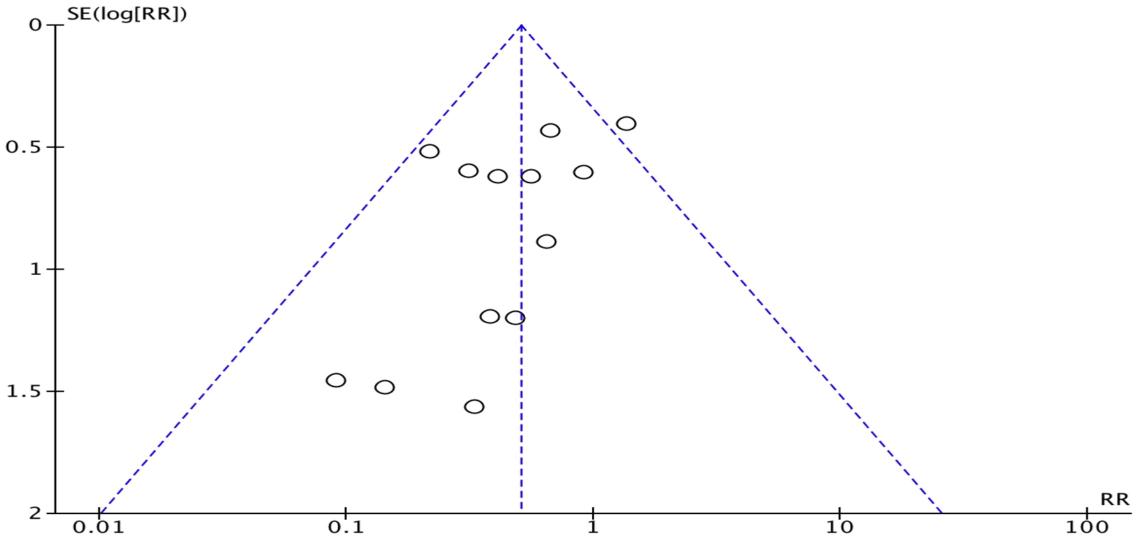

eFigure 14. Funnel plot of volume guarantee ventilation vs. pressure limited ventilation for outcome severe IVH.

Egger's regression intercept

Intercept	-1.25674
Standard error	0.64404
95% lower limit (2-tailed)	-2.67426
95% upper limit (2-tailed)	0.16077
t-value	1.95136
df	11.00000
P-value (1-tailed)	0.03847
P-value (2-tailed)	0.07694

eFigure 15. Egger's regression test for assessing publication bias volume guarantee ventilation vs. pressure limited ventilation for outcome severe IVH

© 2023 Razak A et al. *JAMA Network Open.*

Study or Subgroup	Erythropoietin Events	Erythropoietin Total	Placebo Events	Placebo Total	Weight	Risk Ratio M-H, Fixed, 95% CI
Bierer 2006	0	7	1	8	0.6%	0.38 [0.02, 7.96]
Fauchere 2008	4	30	0	15	0.3%	4.65 [0.27, 81.01]
Fauchere 2015	13	229	10	214	4.3%	1.21 [0.54, 2.71]
Haiden 2005	0	21	2	19	1.1%	0.18 [0.01, 3.56]
Juul 2020	57	476	64	460	27.1%	0.86 [0.62, 1.20]
Maier 1994	2	120	1	121	0.4%	2.02 [0.19, 21.95]
Natalucci 2016	10	191	10	174	4.3%	0.91 [0.39, 2.14]
Ohls 2001	14	146	10	144	4.2%	1.38 [0.63, 3.01]
Ohls 2013	3	32	7	30	3.0%	0.40 [0.11, 1.41]
Peltoniemi 2017	2	21	0	18	0.2%	4.32 [0.22, 84.48]
Song 2016	24	366	60	377	24.6%	0.41 [0.26, 0.65]
Sun 2020	37	950	72	948	30.0%	0.51 [0.35, 0.75]
**Total (95% CI)**		**2589**		**2528**	**100.0%**	**0.68 [0.57, 0.83]**
Total events	166		237			

Heterogeneity: $Chi^2 = 19.93$, df = 11 (P = 0.05); $I^2 = 45\%$

Test for overall effect: Z = 3.92 (P < 0.0001)

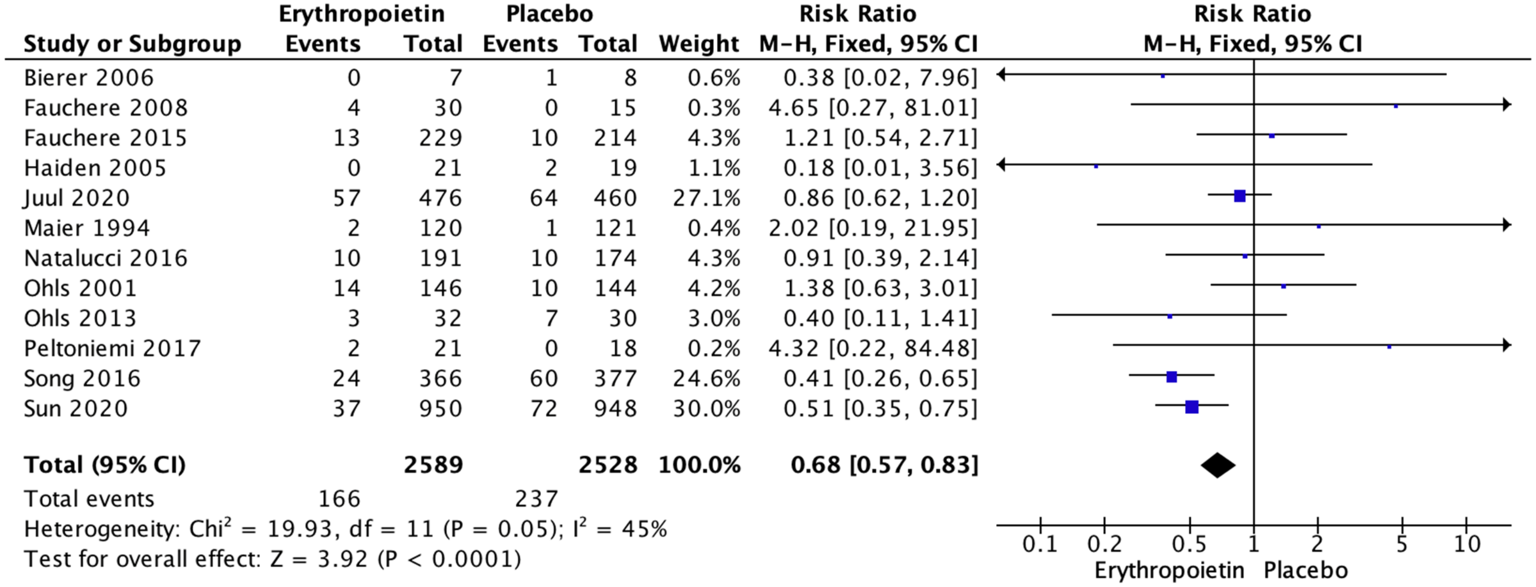

eFigure 16. Forest plot for comparison—Early erythropoiesis-stimulating agents vs. placebo for outcome severe IVH

© 2023 Razak A et al. *JAMA Network Open.*

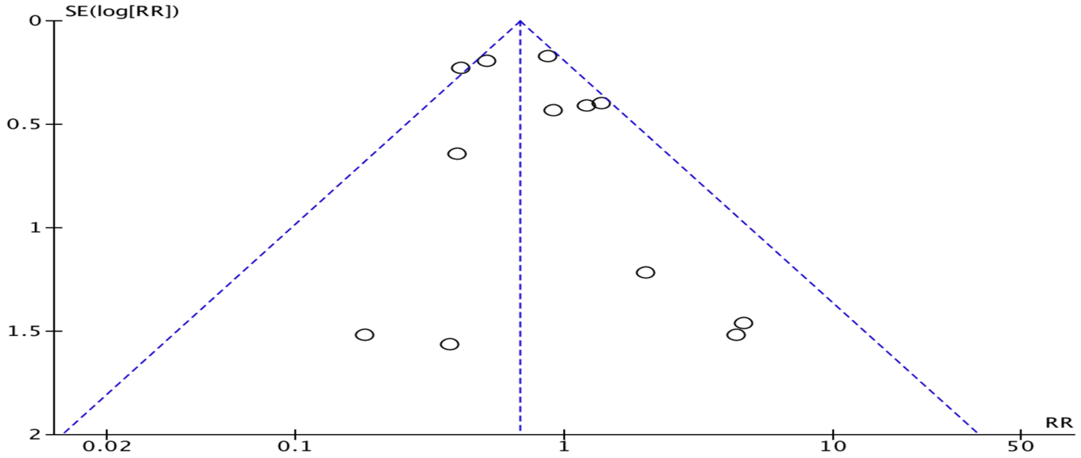

eFigure 17. Funnel plot of Early erythropoiesis-stimulating agents vs. placebo for outcome severe IVH.

© 2023 Razak A et al. *JAMA Network Open.*

Study or Subgroup	Ethamsylate Events	Ethamsylate Total	Placebo Events	Placebo Total	Weight	Risk Ratio M-H, Fixed, 95% CI
Amato 1992	0	10	2	10	3.6%	0.20 [0.01, 3.70]
Benson 1986	8	162	17	168	24.2%	0.49 [0.22, 1.10]
Chen 1993	9	86	20	85	29.2%	0.44 [0.21, 0.92]
EC trial group 1994	21	165	21	169	30.1%	1.02 [0.58, 1.80]
Morgan 1981	5	35	6	35	8.7%	0.83 [0.28, 2.48]
Sanghvi 1999	3	93	3	99	4.2%	1.06 [0.22, 5.14]
**Total (95% CI)**		**551**		**566**	**100.0%**	**0.68 [0.48, 0.97]**
Total events	46		69			

Heterogeneity: $Chi^2 = 5.08$, df = 5 (P = 0.41); $I^2 = 2\%$
Test for overall effect: Z = 2.16 (P = 0.03)

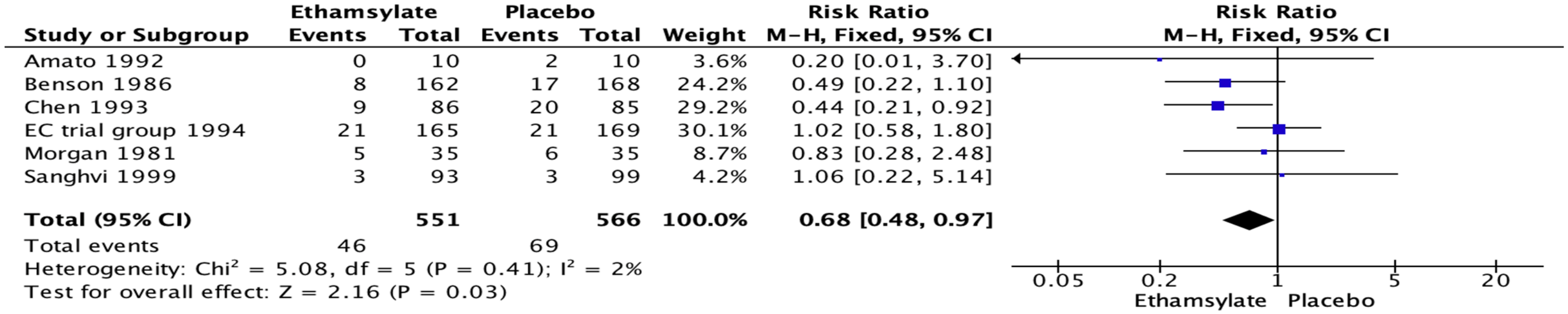

eFigure 18. Forest plot for comparison— prophylactic ethamsylate administration vs. placebo for the outcome severe IVH.

Study or Subgroup	Supine head Midline Events	Supine head Midline Total	Supine head Rotated Events	Supine head Rotated Total	Weight	Risk Ratio M-H, Fixed, 95% CI
Al Abdi 2011	2	23	1	25	4.8%	2.17 [0.21, 22.40]
Al Abdi 2015	1	31	1	31	5.0%	1.00 [0.07, 15.28]
Kochan 2019	11	90	18	90	90.2%	0.61 [0.31, 1.22]
**Total (95% CI)**		**144**		**146**	**100.0%**	**0.71 [0.37, 1.33]**
Total events	14		20			

Heterogeneity: $Chi^2 = 1.12$, df = 2 (P = 0.57); $I^2 = 0\%$
Test for overall effect: Z = 1.08 (P = 0.28)

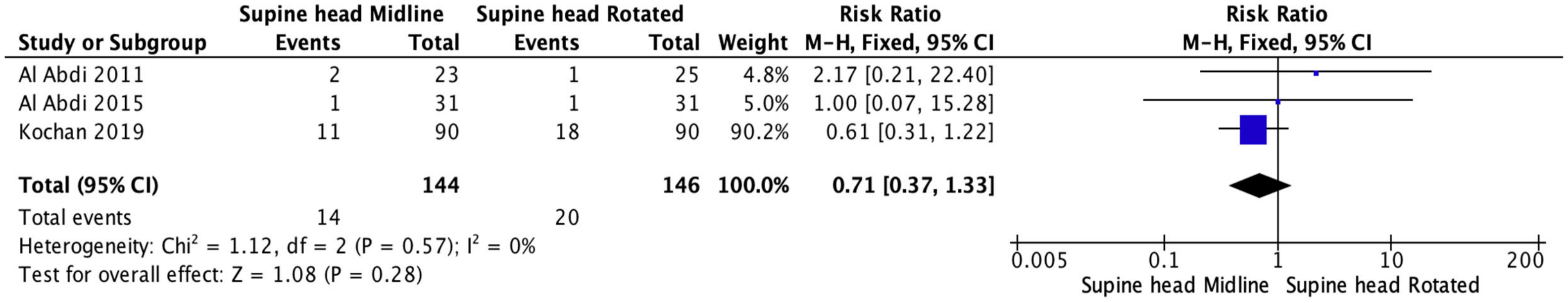

eFigure 19. Forest plot for comparison— Supine head midline vs. supine head rotated for the outcome severe IVH.

Study or Subgroup	LISA Events	LISA Total	INSURE Events	INSURE Total	Weight	Risk Ratio M-H, Fixed, 95% CI
Bao 2015	1	47	0	43	1.1%	2.75 [0.12, 65.76]
Gopel 2011	8	108	6	112	13.0%	1.38 [0.50, 3.85]
Gupta 2020	0	29	1	29	3.3%	0.33 [0.01, 7.86]
Han 2020	10	151	10	147	22.3%	0.97 [0.42, 2.27]
Jena 2019	5	175	4	175	8.8%	1.25 [0.34, 4.58]
Kribs 2015	11	107	23	104	51.4%	0.46 [0.24, 0.90]
**Total (95% CI)**		**617**		**610**	**100.0%**	**0.79 [0.52, 1.20]**
Total events	35		44			

Heterogeneity: $Chi^2 = 5.17$, df = 5 (P = 0.39); $I^2 = 3\%$
Test for overall effect: Z = 1.11 (P = 0.27)

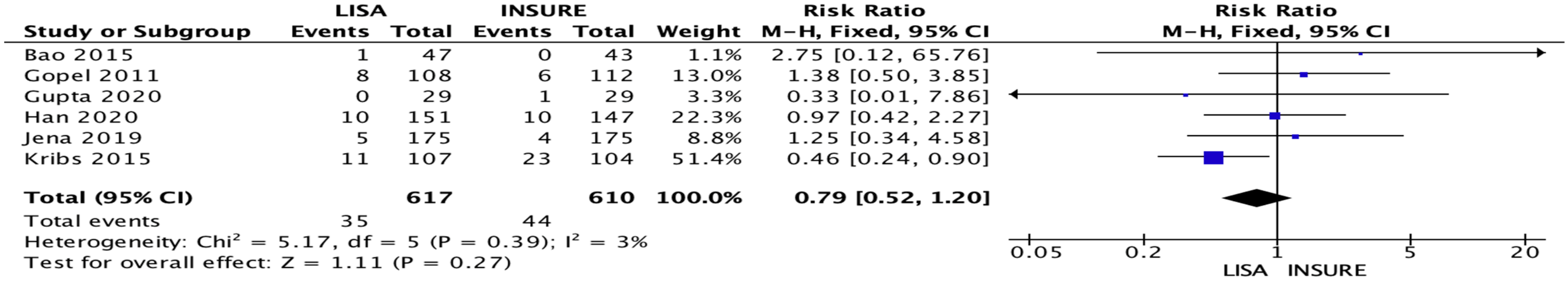

© 2023 Razak A et al. *JAMA Network Open.*

eFigure 20. Forest plot for comparison— LISA vs. INSURE method for surfactant administration for the outcome severe IVH

Study or Subgroup	High Frequency Vent. Events	High Frequency Vent. Total	Conventional Vent. Events	Conventional Vent. Total	Weight	Risk Ratio M-H, Fixed, 95% CI
Clark 1992	6	30	6	26	2.2%	0.87 [0.32, 2.36]
Courtney 2002	45	244	45	254	15.3%	1.04 [0.72, 1.51]
Craft 2003	4	22	5	24	1.7%	0.87 [0.27, 2.84]
Durand 2001	1	24	4	24	1.4%	0.25 [0.03, 2.08]
Gerstmann 1996	2	64	6	61	2.1%	0.32 [0.07, 1.51]
HIFI 1989	84	327	63	346	21.3%	1.41 [1.06, 1.88]
HiFO study Group 1993	6	81	2	84	0.7%	3.11 [0.65, 14.97]
Johnson 2002	38	390	55	392	19.1%	0.69 [0.47, 1.02]
Lista 2008	1	19	1	21	0.3%	1.11 [0.07, 16.47]
Moriette 2001	34	139	19	134	6.7%	1.73 [1.04, 2.87]
Ogawa 1993	2	46	1	46	0.3%	2.00 [0.19, 21.30]
Plavka 1999	0	19	1	18	0.5%	0.32 [0.01, 7.30]
Rettwitz-Volk 1998	5	46	2	50	0.7%	2.72 [0.55, 13.33]
Salvo 2012	3	39	6	39	2.1%	0.50 [0.13, 1.86]
Scheiber 2003	18	102	14	105	4.8%	1.32 [0.70, 2.52]
Sun 2014	30	177	27	179	9.3%	1.12 [0.70, 1.81]
Thome 1999	19	140	18	144	6.2%	1.09 [0.60, 1.98]
Van Reempts 2003	14	147	13	153	4.4%	1.12 [0.55, 2.30]
Vento 2005	3	20	2	20	0.7%	1.50 [0.28, 8.04]
**Total (95% CI)**		**2076**		**2120**	**100.0%**	**1.11 [0.96, 1.29]**
Total events	315		290			

Heterogeneity: $Chi^2 = 21.54, df = 18 (P = 0.25); I^2 = 16\%$
Test for overall effect: Z = 1.41 (P = 0.16)

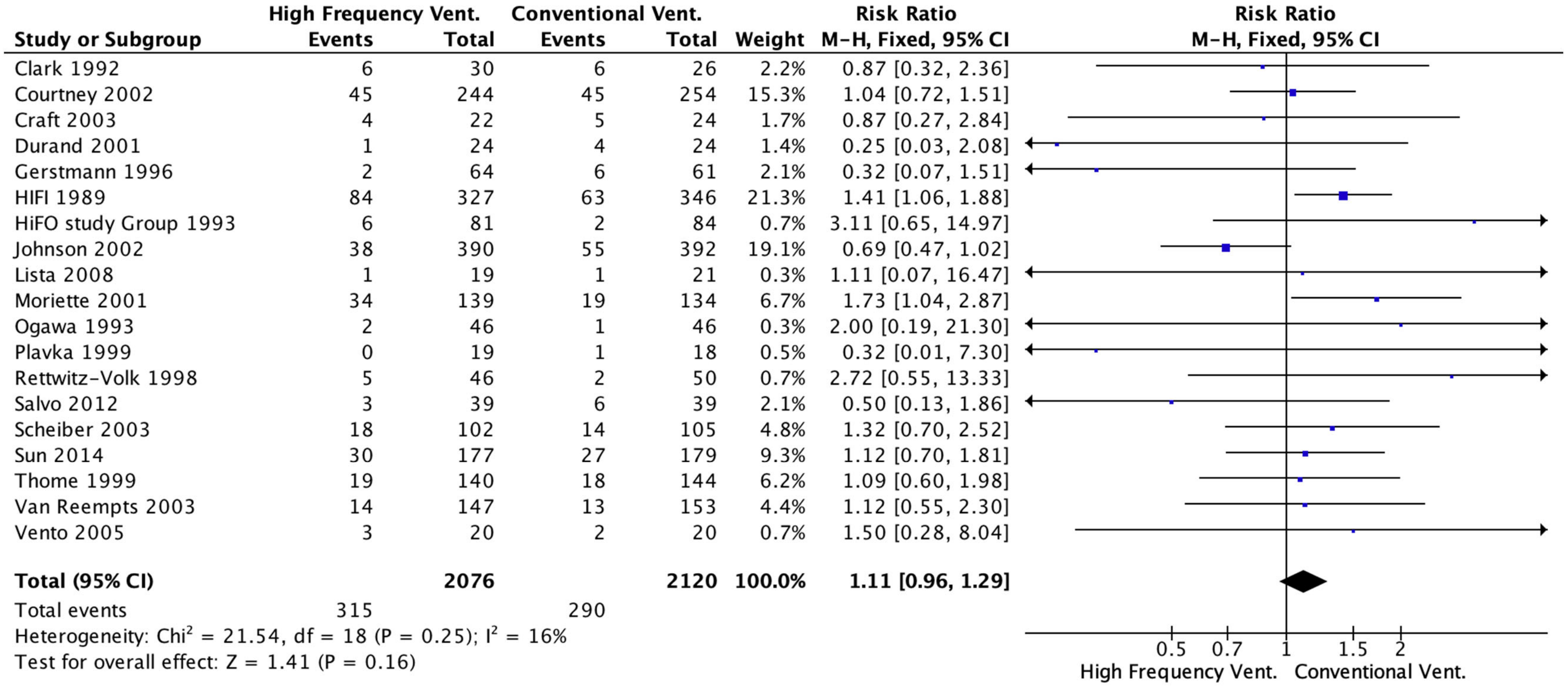

eFigure 21. Forest plot for comparison— Elective HFOV vs. conventional ventilation method for the outcome severe IVH.

© 2023 Razak A et al. *JAMA Network Open.*

Study or Subgroup	Jet Ventilation Events	Jet Ventilation Total	Conventional Ventilation Events	Conventional Ventilation Total	Weight	Risk Ratio M-H, Fixed, 95% CI
Keszler 1997	9	61	9	59	53.0%	0.97 [0.41, 2.27]
Wiswell 1996	15	37	8	36	47.0%	1.82 [0.88, 3.77]
**Total (95% CI)**		**98**		**95**	**100.0%**	**1.37 [0.79, 2.37]**
Total events	24		17			

Heterogeneity: $Chi^2 = 1.24$, df = 1 (P = 0.27); $I^2 = 19\%$
Test for overall effect: Z = 1.13 (P = 0.26)

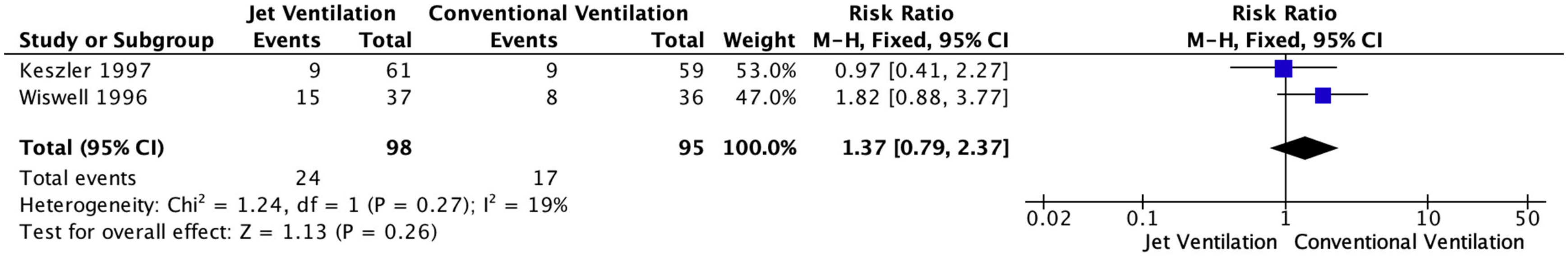

eFigure 22. Forest plot for comparison— Elective HFJV vs. conventional ventilation method for the outcome severe IVH.

Study or Subgroup	High Oxygen Sats Events	High Oxygen Sats Total	Low Oxygen Sats Events	Low Oxygen Sats Total	Weight	Risk Ratio M-H, Fixed, 95% CI
BOOST-II Australia 2016	56	566	58	565	25.9%	0.96 [0.68, 1.37]
BOOST-II NZ 2014	12	169	13	170	5.8%	0.93 [0.44, 1.98]
BOOST-II UK 2016	58	476	69	468	31.0%	0.83 [0.60, 1.14]
Voucher (SUPPORT Trial) 2010	81	640	83	630	37.3%	0.96 [0.72, 1.28]
**Total (95% CI)**		**1851**		**1833**	**100.0%**	**0.92 [0.77, 1.10]**
Total events	207		223			

Heterogeneity: $Chi^2 = 0.57$, df = 3 (P = 0.90); $I^2 = 0\%$
Test for overall effect: Z = 0.95 (P = 0.34)

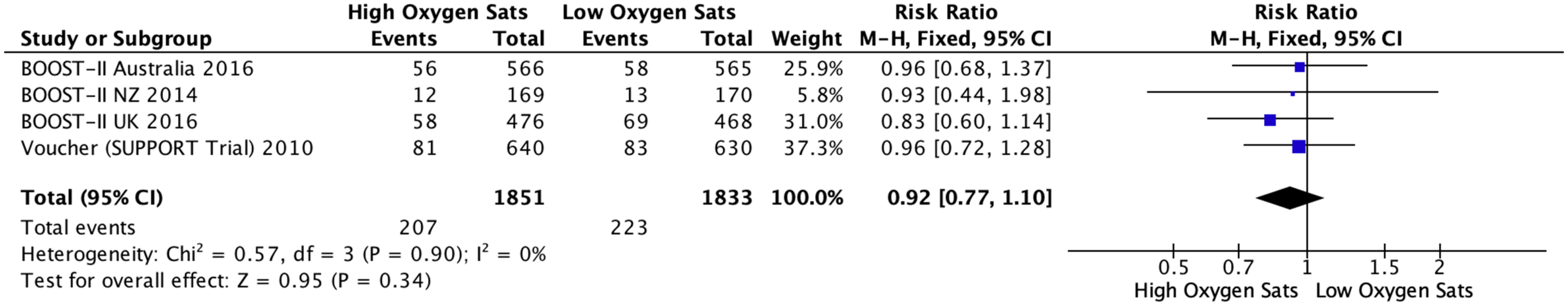

eFigure 23. Forest plot for comparison— high oxygen saturation target (91-95%) vs low oxygen saturation target (85-89%) in NICU for the outcome severe IVH.

© 2023 Razak A et al. *JAMA Network Open.*

Study or Subgroup	Permissive Hypercapnia Events	Permissive Hypercapnia Total	Normal PCO2 Events	Normal PCO2 Total	Weight	Risk Ratio M-H, Fixed, 95% CI
Carlo 1999	21	109	29	111	32.1%	0.74 [0.45, 1.21]
Carlo 2002	20	109	26	111	28.8%	0.78 [0.47, 1.32]
Mariani 1999	7	24	5	25	5.5%	1.46 [0.54, 3.97]
Thome 2006	8	33	9	32	10.2%	0.86 [0.38, 1.95]
Thome 2015	26	178	21	180	23.4%	1.25 [0.73, 2.14]
**Total (95% CI)**		**453**		**459**	**100.0%**	**0.92 [0.71, 1.21]**
Total events	82		90			

Heterogeneity: $Chi^2 = 3.24$, df = 4 (P = 0.52); $I^2 = 0\%$
Test for overall effect: Z = 0.59 (P = 0.56)

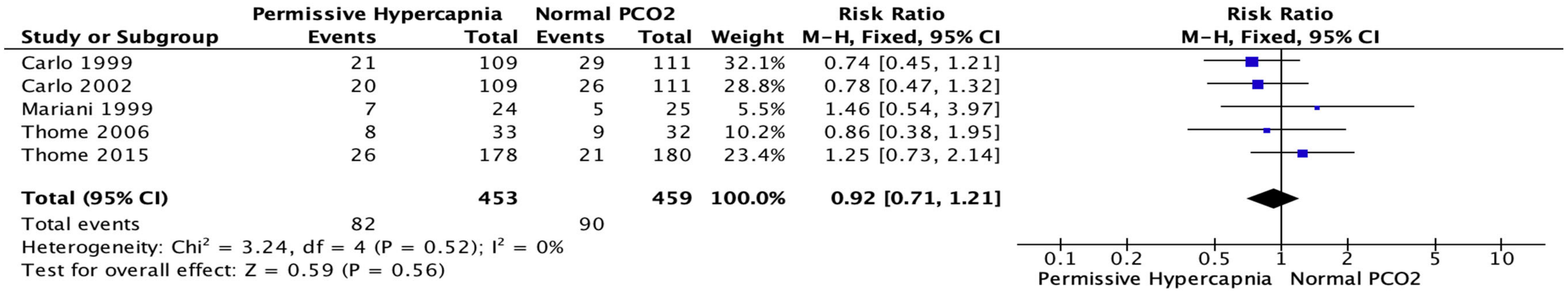

eFigure 24. Forest plot for comparison— permissive hypercapnia vs. normocapnia during ventilation for the outcome severe IVH.

Study or Subgroup	Early Extubation Events	Early Extubation Total	Delayed Extubation Events	Delayed Extubation Total	Weight	Risk Ratio M-H, Fixed, 95% CI
Danan 2008	2	44	6	42	100.0%	0.32 [0.07, 1.49]
**Total (95% CI)**		**44**		**42**	**100.0%**	**0.32 [0.07, 1.49]**
Total events	2		6			

Heterogeneity: Not applicable
Test for overall effect: Z = 1.45 (P = 0.15)

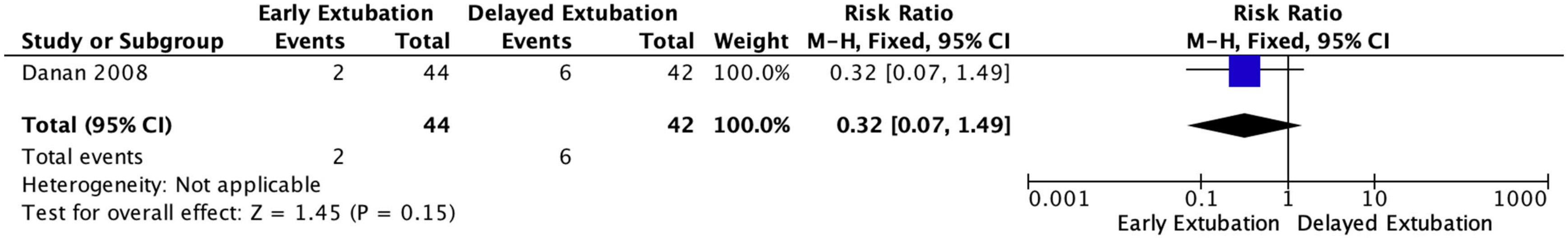

eFigure 25. Forest plot for comparison— early extubation vs. delayed extubation during ventilation for the outcome severe IVH.

Study or Subgroup	Caffeine Events	Caffeine Total	Placebo Events	Placebo Total	Weight	Risk Ratio M-H, Fixed, 95% CI
Amaro 2018	12	41	6	42	4.8%	2.05 [0.85, 4.94]
Cap Trial 2006	99	967	115	966	93.9%	0.86 [0.67, 1.11]
Ramin 2022	0	45	1	45	1.2%	0.33 [0.01, 7.97]
**Total (95% CI)**		**1053**		**1053**	**100.0%**	**0.91 [0.72, 1.16]**
Total events	111		122			

Heterogeneity: $Chi^2 = 3.84$, df = 2 (P = 0.15); $I^2 = 48\%$
Test for overall effect: Z = 0.76 (P = 0.45)

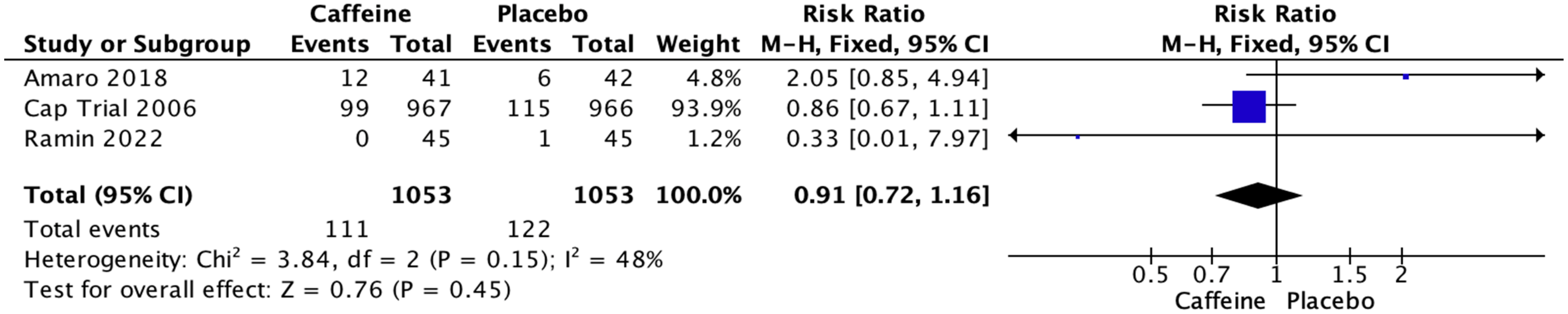

eFigure 26. Forest plot for comparison—Caffeine prophylaxis or treatment for apnea or post-extubation vs. placebo for the outcome severe IVH.

© 2023 Razak A et al. *JAMA Network Open.*

Study or Subgroup	High Dose caffeine Events	Total	Standard Dose caffeine Events	Total	Weight	Risk Ratio M-H, Fixed, 95% CI
Gray 2010	7	120	4	126	21.7%	1.84 [0.55, 6.12]
McPherson 2015	4	37	4	37	22.3%	1.00 [0.27, 3.70]
Mohammed 2015	7	60	5	60	27.8%	1.40 [0.47, 4.17]
Mohd Kori 2021	0	40	2	38	14.3%	0.19 [0.01, 3.84]
Steer 2003	2	45	0	40	2.9%	4.46 [0.22, 90.14]
Vesoulis 2018	3	29	2	30	10.9%	1.55 [0.28, 8.62]
**Total (95% CI)**		**331**		**331**	**100.0%**	**1.34 [0.74, 2.41]**
Total events	23		17			
Heterogeneity: Chi² = 2.73, df = 5 (P = 0.74); I² = 0%						
Test for overall effect: Z = 0.97 (P = 0.33)						

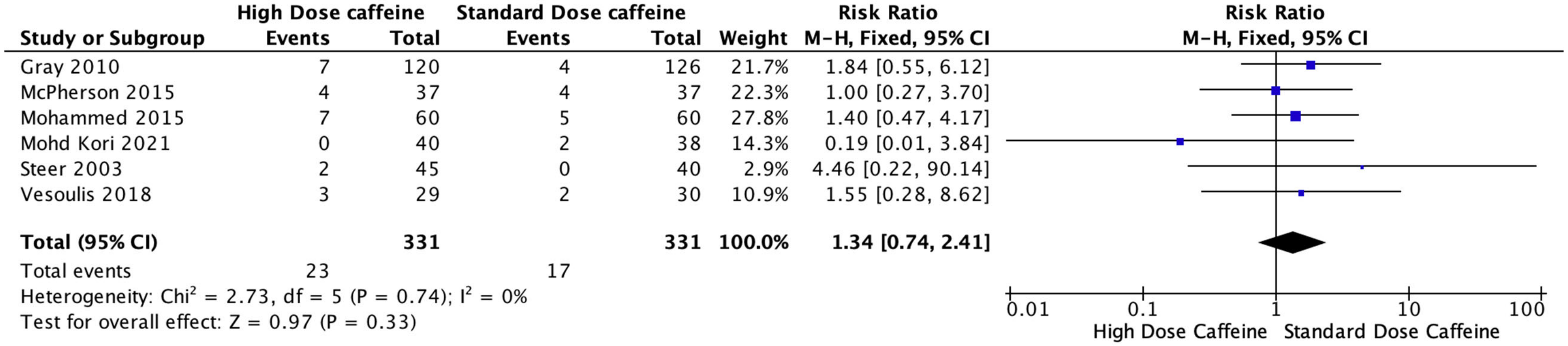

eFigure 27. Forest plot for comparison—high dose vs. low dose caffeine prophylaxis for apnea or post-extubation for the outcome severe IVH.

Study or Subgroup	Midazolam Events	Total	Placebo Events	Total	Weight	Risk Ratio M-H, Fixed, 95% CI
Anand 1999	5	22	3	21	100.0%	1.59 [0.43, 5.84]
**Total (95% CI)**		**22**		**21**	**100.0%**	**1.59 [0.43, 5.84]**
Total events	5		3			
Heterogeneity: Not applicable						
Test for overall effect: Z = 0.70 (P = 0.48)						

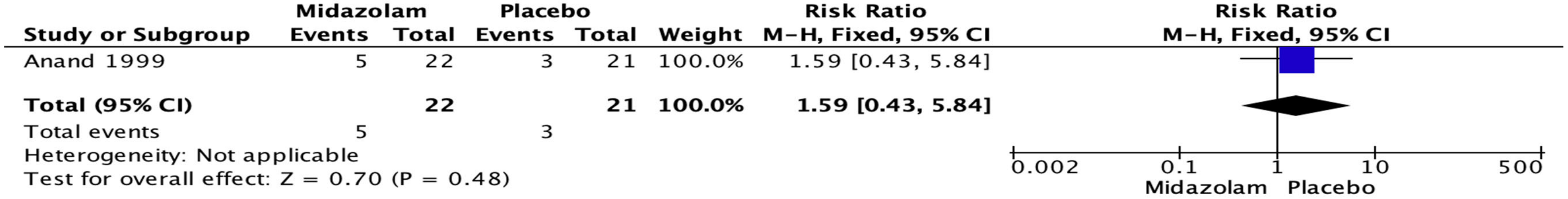

eFigure 28. Forest plot for comparison— Sedation during ventilation: midazolam vs. placebo for the outcome severe IVH.

Study or Subgroup	Opiods Events	Total	Placebo Events	Total	Weight	Risk Ratio M-H, Fixed, 95% CI
Anand 1999	0	22	3	21	6.0%	0.14 [0.01, 2.50]
Anand 2004	55	411	46	429	75.8%	1.25 [0.86, 1.80]
Lago1998	0	26	2	27	4.1%	0.21 [0.01, 4.12]
Simons 2003	3	73	7	77	11.5%	0.45 [0.12, 1.68]
Siwiec 1999	0	10	1	10	2.5%	0.33 [0.02, 7.32]
**Total (95% CI)**		**542**		**564**	**100.0%**	**1.02 [0.73, 1.43]**
Total events	58		59			
Heterogeneity: Chi² = 6.05, df = 4 (P = 0.20); I² = 34%						
Test for overall effect: Z = 0.14 (P = 0.89)						

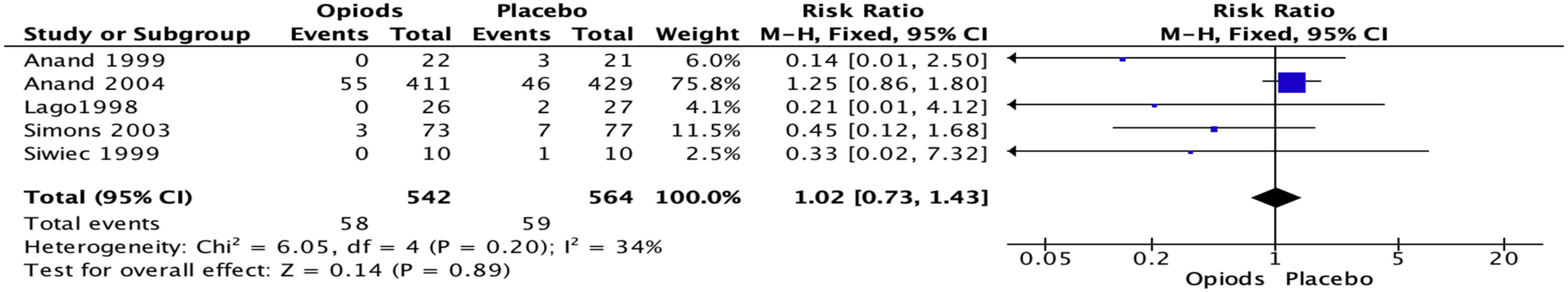

eFigure 29. Forest plot for comparison— Sedation during ventilation: opioid vs. placebo for the outcome severe IVH.

© 2023 Razak A et al. *JAMA Network Open.*

Study or Subgroup	Muscle Relaxant Events	Muscle Relaxant Total	Placebo Events	Placebo Total	Weight	Risk Ratio M-H, Fixed, 95% CI
Perlman 1985	0	14	7	10	46.5%	0.05 [0.00, 0.77]
Shaw 1993	9	96	10	97	53.5%	0.91 [0.39, 2.14]
**Total (95% CI)**		**110**		**107**	**100.0%**	**0.51 [0.25, 1.06]**
Total events	9		17			
Heterogeneity: $Chi^2 = 4.55$, df = 1 (P = 0.03); $I^2 = 78\%$						
Test for overall effect: Z = 1.82 (P = 0.07)						

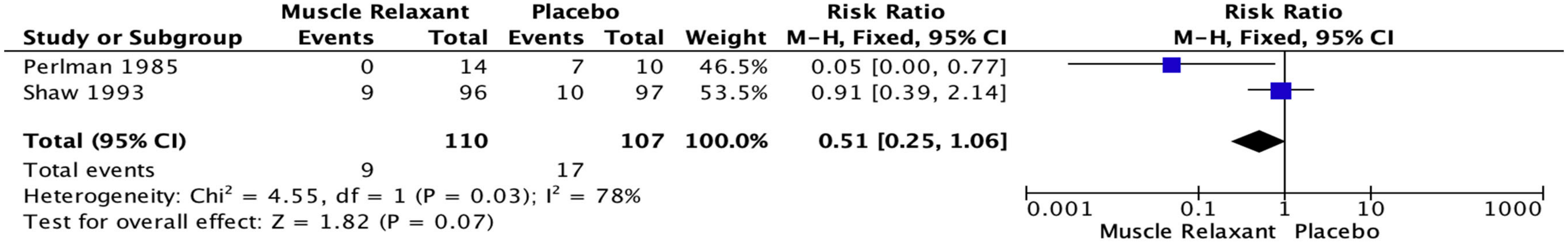

eFigure 30. Forest plot for comparison— Neuromuscular paralysis during ventilation vs. placebo for the outcome severe IVH.

Study or Subgroup	Albumin Events	Albumin Total	Dopamine Events	Dopamine Total	Weight	Risk Ratio M-H, Fixed, 95% CI
Gill 1993	17	20	11	19	100.0%	1.47 [0.96, 2.25]
**Total (95% CI)**		**20**		**19**	**100.0%**	**1.47 [0.96, 2.25]**
Total events	17		11			
Heterogeneity: Not applicable						
Test for overall effect: Z = 1.77 (P = 0.08)						

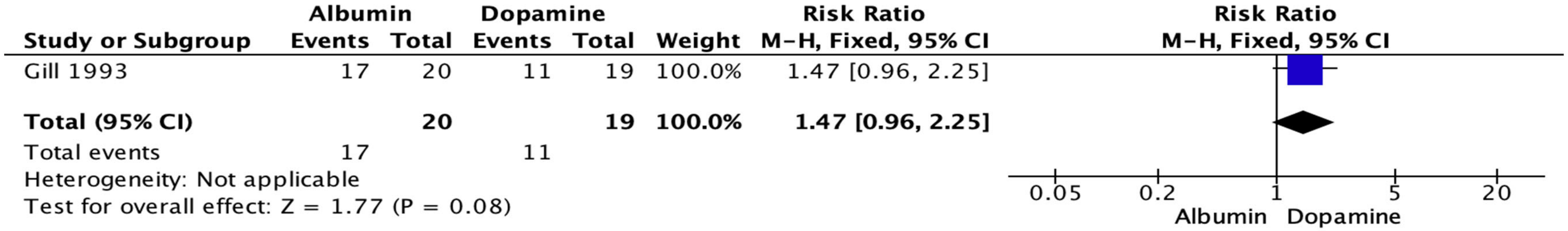

eFigure 31. Forest plot for comparison— volume expansion vs. inotropes (any) for hypotension for the outcome severe IVH.

Study or Subgroup	Dopamine Events	Dopamine Total	Dobutamine Events	Dobutamine Total	Weight	Risk Ratio M-H, Fixed, 95% CI
Hentschel 1995	0	10	1	10	43.2%	0.33 [0.02, 7.32]
Klarr 1994	2	31	2	32	56.8%	1.03 [0.15, 6.88]
**Total (95% CI)**		**41**		**42**	**100.0%**	**0.73 [0.15, 3.50]**
Total events	2		3			
Heterogeneity: $Chi^2 = 0.38$, df = 1 (P = 0.54); $I^2 = 0\%$						
Test for overall effect: Z = 0.39 (P = 0.69)						

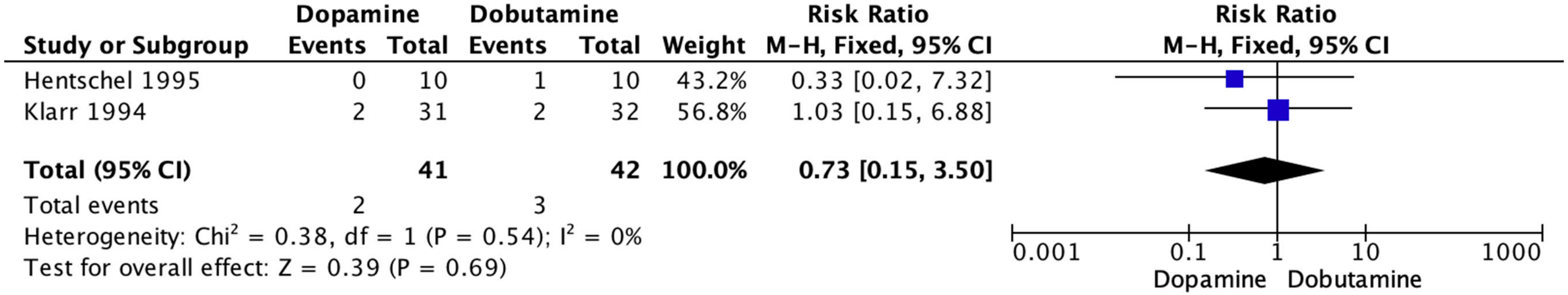

eFigure 32. Forest plot for comparison— Dopamine vs. dobutamine for hypotension for the outcome severe IVH.

© 2023 Razak A et al. *JAMA Network Open.*

Study or Subgroup	Ibuprofen Events	Ibuprofen Total	Placebo Events	Placebo Total	Weight	Risk Ratio M-H, Fixed, 95% CI
Dani 2000	0	40	0	40		Not estimable
Dani 2005	8	77	8	78	14.4%	1.01 [0.40, 2.56]
De Carolis 2000	2	23	1	23	1.8%	2.00 [0.19, 20.55]
Gournay 2004	7	65	15	66	27.0%	0.47 [0.21, 1.09]
Kalani 2016 (a)	0	31	6	31	11.8%	0.08 [0.00, 1.31]
Kanmaz 2013	1	19	6	17	11.5%	0.15 [0.02, 1.12]
Van Overmeire 2004	17	205	18	210	32.3%	0.97 [0.51, 1.82]
Varvarigou 1996	2	23	0	11	1.2%	2.50 [0.13, 48.06]
**Total (95% CI)**		**483**		**476**	**100.0%**	**0.68 [0.46, 1.01]**
Total events	37		54			

Heterogeneity: $Chi^2 = 8.65$, df = 6 (P = 0.19); $I^2 = 31\%$

Test for overall effect: Z = 1.92 (P = 0.05)

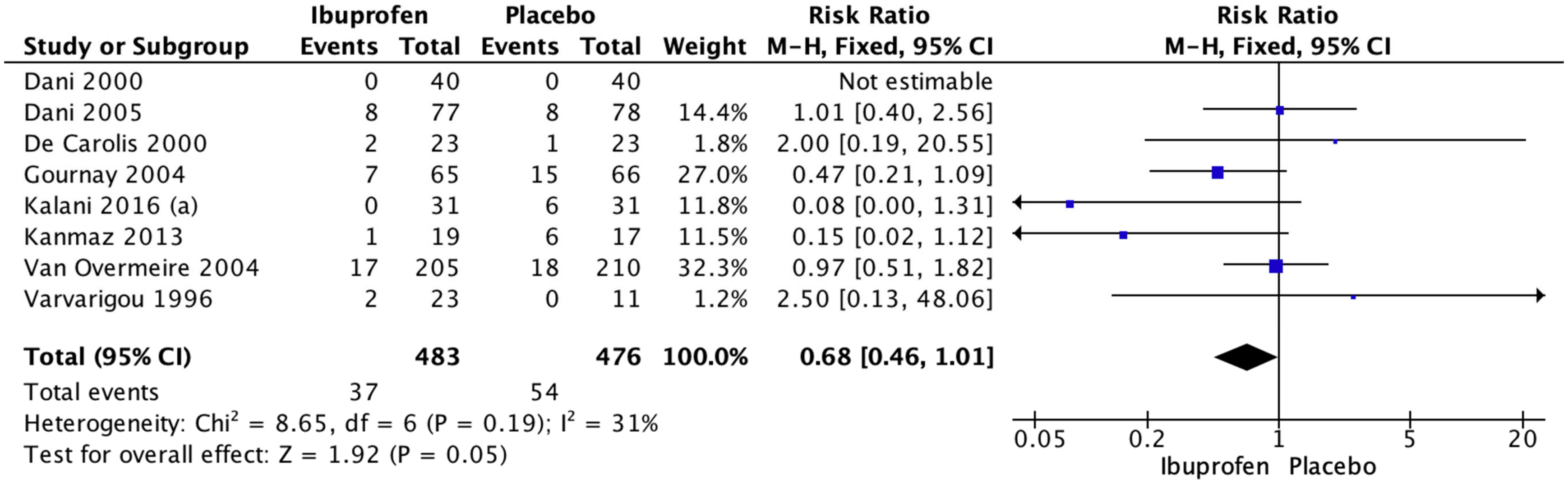

eFigure 33. Forest plot for comparison— Ibuprofen prophylaxis for PDA vs. placebo for the outcome severe IVH.

Study or Subgroup	Indomethacin Events	Indomethacin Total	Placebo Events	Placebo Total	Weight	Risk Ratio M-H, Random, 95% CI
Kluckow 2014	2	44	6	48	100.0%	0.36 [0.08, 1.71]
**Total (95% CI)**		**44**		**48**	**100.0%**	**0.36 [0.08, 1.71]**
Total events	2		6			

Heterogeneity: Not applicable

Test for overall effect: Z = 1.28 (P = 0.20)

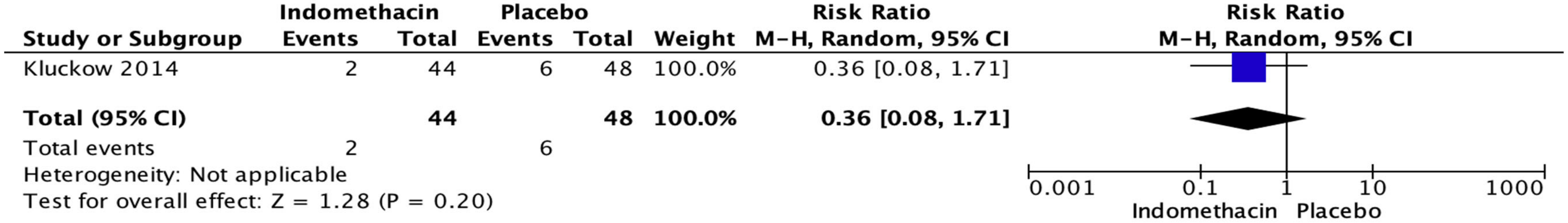

eFigure 34. Forest plot for comparison— Indomethacin pre-symptomatic treatment for PDA vs. placebo for the outcome severe IVH.

© 2023 Razak A et al. *JAMA Network Open.*

Study or Subgroup	Ibuprofen Events	Ibuprofen Total	Placebo Events	Placebo Total	Weight	Risk Ratio M-H, Random, 95% CI
Aranda 2009	11	67	11	67	36.5%	1.00 [0.47, 2.15]
Roze (TRIOCAPI) 2020	18	114	11	114	43.0%	1.64 [0.81, 3.31]
Sosenko 2012	6	54	7	51	20.4%	0.81 [0.29, 2.25]
**Total (95% CI)**		**235**		**232**	**100.0%**	**1.18 [0.75, 1.88]**
Total events	35		29			

Heterogeneity: $Tau^2 = 0.00$; $Chi^2 = 1.53$, df = 2 (P = 0.46); $I^2 = 0\%$
Test for overall effect: Z = 0.72 (P = 0.47)

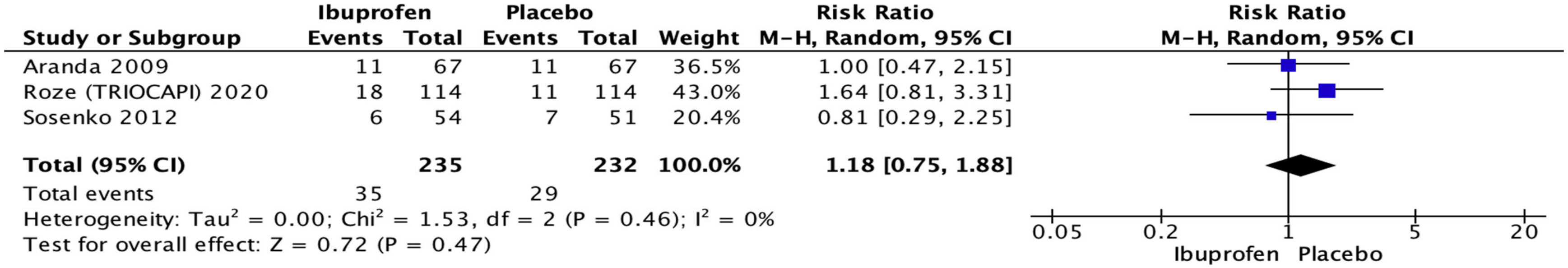

eFigure 35. Forest plot for comparison— Ibuprofen pre-symptomatic treatment for PDA vs. placebo for the outcome severe IVH.

Study or Subgroup	Liberal transfusion Events	Liberal transfusion Total	Restrictive transfusion Events	Restrictive transfusion Total	Weight	Risk Ratio M-H, Fixed, 95% CI
Bell 2005	8	51	5	49	12.6%	1.54 [0.54, 4.38]
Chen 2009	2	16	1	17	2.4%	2.13 [0.21, 21.22]
Connnelly 1999	1	11	1	13	2.3%	1.18 [0.08, 16.78]
Franz 2020	27	479	35	521	82.8%	0.84 [0.52, 1.36]
**Total (95% CI)**		**557**		**600**	**100.0%**	**0.97 [0.63, 1.47]**
Total events	38		42			

Heterogeneity: $Chi^2 = 1.55$, df = 3 (P = 0.67); $I^2 = 0\%$
Test for overall effect: Z = 0.16 (P = 0.87)

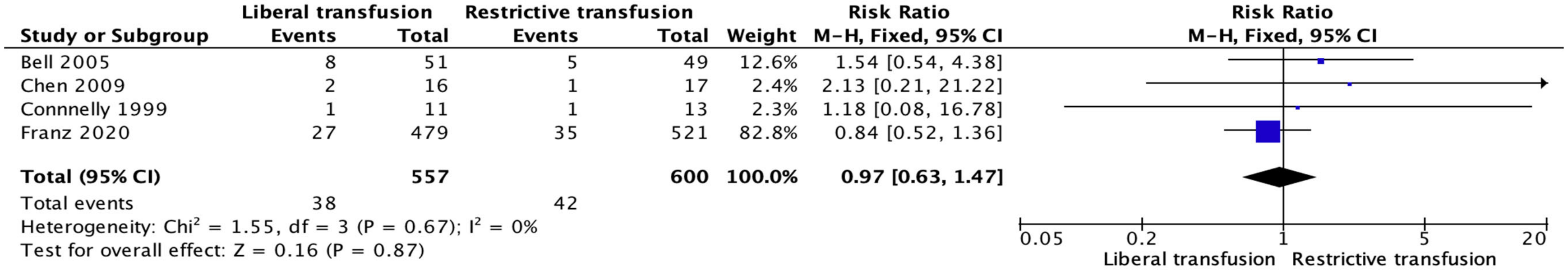

eFigure 36. Forest plot for comparison— Restrictive vs. liberal packed red cell transfusion for anemia for the outcome severe IVH.

Study or Subgroup	Plasma Events	Plasma Total	Placebo Events	Placebo Total	Weight	Risk Ratio M-H, Fixed, 95% CI
Beverley 1985	3	36	8	37	33.1%	0.39 [0.11, 1.34]
NNI Trial Group 1996	13	257	16	258	66.9%	0.82 [0.40, 1.66]
**Total (95% CI)**		**293**		**295**	**100.0%**	**0.67 [0.37, 1.24]**
Total events	16		24			

Heterogeneity: $Chi^2 = 1.05$, df = 1 (P = 0.31); $I^2 = 5\%$
Test for overall effect: Z = 1.27 (P = 0.20)

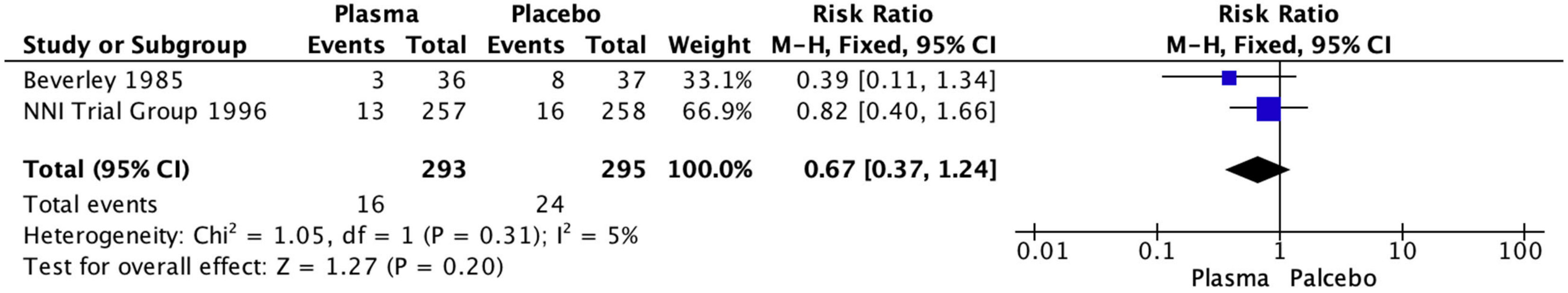

eFigure 37. Forest plot for comparison— Prophylactic plasma administration vs. placebo for the outcome severe IVH.

© 2023 Razak A et al. *JAMA Network Open.*

Study or Subgroup	Antithrombin III Events	Antithrombin III Total	Placebo Events	Placebo Total	Weight	Risk Ratio M-H, Fixed, 95% CI
Fulia 2003	4	30	6	30	60.6%	0.67 [0.21, 2.13]
Schmidt 1998	5	56	4	59	39.4%	1.32 [0.37, 4.66]
**Total (95% CI)**		**86**		**89**	**100.0%**	**0.92 [0.40, 2.14]**
Total events	9		10			

Heterogeneity: $Chi^2 = 0.61$, df = 1 (P = 0.44); $I^2 = 0\%$

Test for overall effect: Z = 0.19 (P = 0.85)

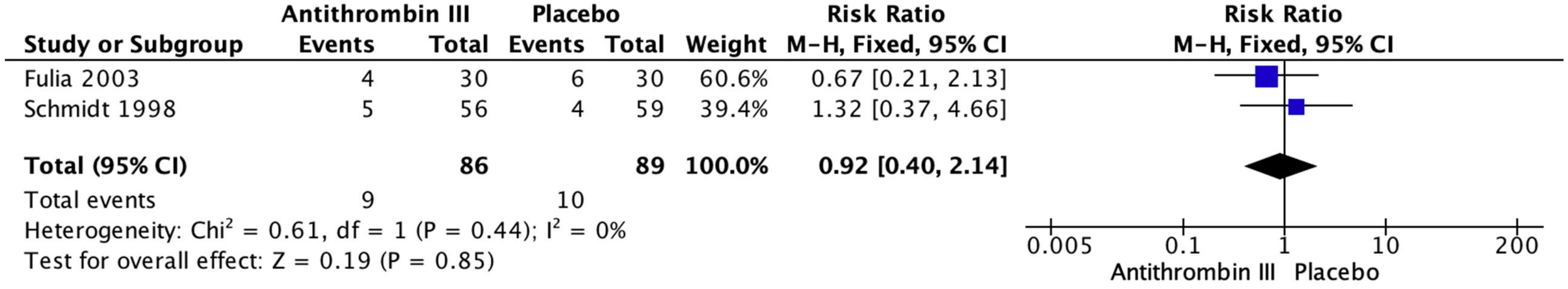

eFigure 38. Forest plot for comparison— Prophylactic antithrombin III administration vs. placebo for the outcome severe IVH.

Study or Subgroup	Heparin Events	Heparin Total	Placebo Events	Placebo Total	Weight	Risk Ratio M-H, Fixed, 95% CI
Chang 1997	6	53	7	54	100.0%	0.87 [0.31, 2.43]
**Total (95% CI)**		**53**		**54**	**100.0%**	**0.87 [0.31, 2.43]**
Total events	6		7			

Heterogeneity: Not applicable

Test for overall effect: Z = 0.26 (P = 0.80)

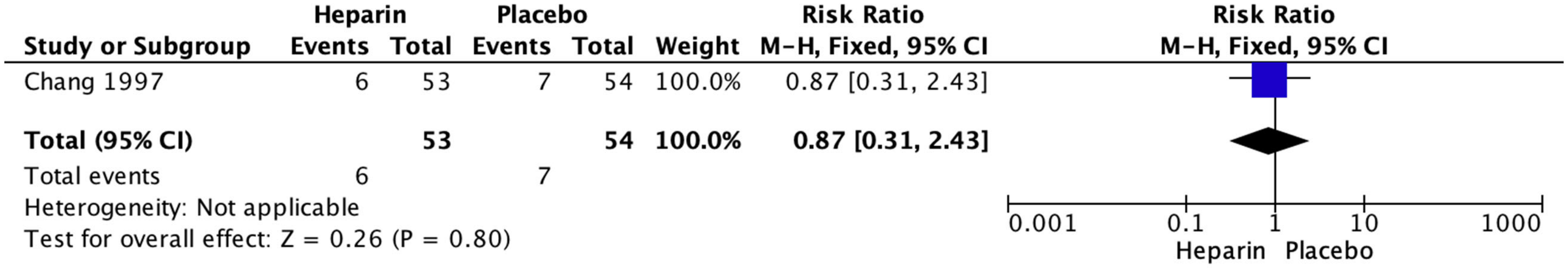

eFigure 39. Forest plot for comparison— Prophylactic heparin administration vs. placebo for the outcome severe IVH.

© 2023 Razak A et al. *JAMA Network Open.*

Study or Subgroup	Vitamin A Events	Vitamin A Total	Placebo Events	Placebo Total	Weight	Risk Ratio M-H, Fixed, 95% CI
Bental 1994	5	31	4	29	3.6%	1.17 [0.35, 3.93]
Kiatchoosakun 2014	0	40	2	40	2.2%	0.20 [0.01, 4.04]
Mactier 2012	5	42	3	46	2.5%	1.83 [0.46, 7.18]
Papagaroufalis 1991	3	23	12	24	10.3%	0.26 [0.08, 0.81]
Ravishankar 2003	6	22	9	18	8.6%	0.55 [0.24, 1.24]
Sun 2020	4	132	5	130	4.4%	0.79 [0.22, 2.87]
Tyson 1999	73	405	78	402	68.4%	0.93 [0.70, 1.24]
**Total (95% CI)**		**695**		**689**	**100.0%**	**0.84 [0.65, 1.07]**
Total events	96		113			

Heterogeneity: $Chi^2 = 8.06$, df = 6 (P = 0.23); $I^2 = 26\%$
Test for overall effect: Z = 1.43 (P = 0.15)

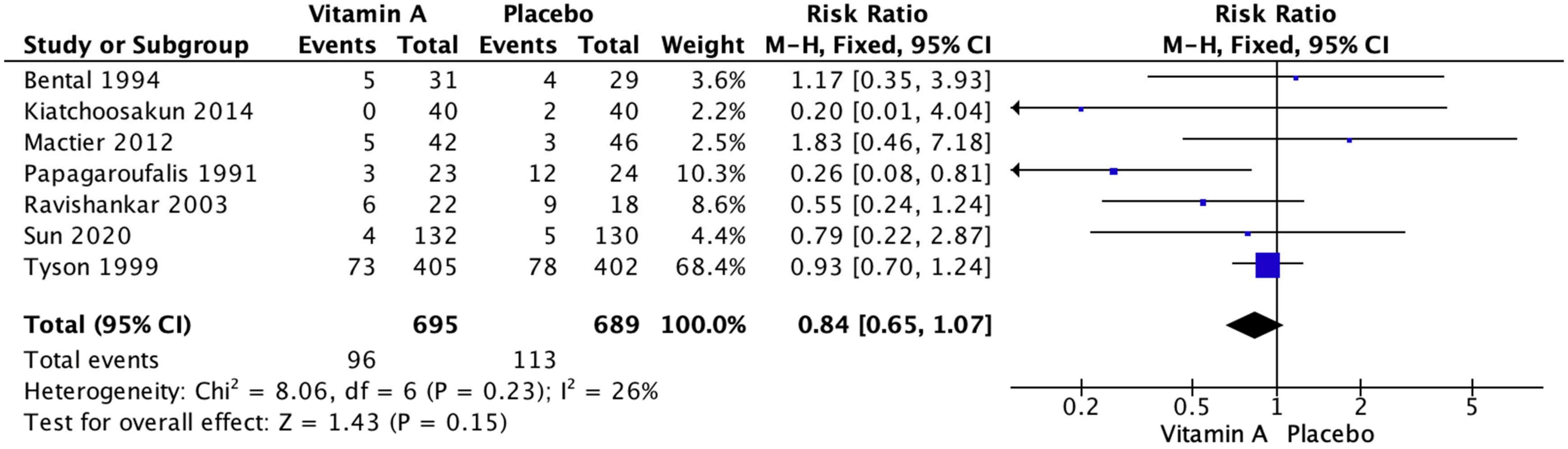

eFigure 40. Forest plot for comparison— Vitamin A supplementation vs. placebo for the outcome severe IVH.

Study or Subgroup	Vitamin E Events	Vitamin E Total	Placebo Events	Placebo Total	Weight	Risk Ratio M-H, Fixed, 95% CI
Barekatein 2018	0	38	2	38	4.5%	0.20 [0.01, 4.03]
Fish 1990	11	73	19	79	32.6%	0.63 [0.32, 1.23]
Hittner 1981	11	50	8	51	14.1%	1.40 [0.62, 3.19]
Hittner 1984	3	68	3	67	5.4%	0.99 [0.21, 4.71]
Phelps 1987	23	140	15	147	26.1%	1.61 [0.88, 2.96]
Sinha 1987	2	102	7	108	12.1%	0.30 [0.06, 1.42]
Speer 1984	2	64	3	70	5.1%	0.73 [0.13, 4.22]
**Total (95% CI)**		**535**		**560**	**100.0%**	**0.96 [0.68, 1.36]**
Total events	52		57			

Heterogeneity: $Chi^2 = 8.43$, df = 6 (P = 0.21); $I^2 = 29\%$
Test for overall effect: Z = 0.23 (P = 0.82)

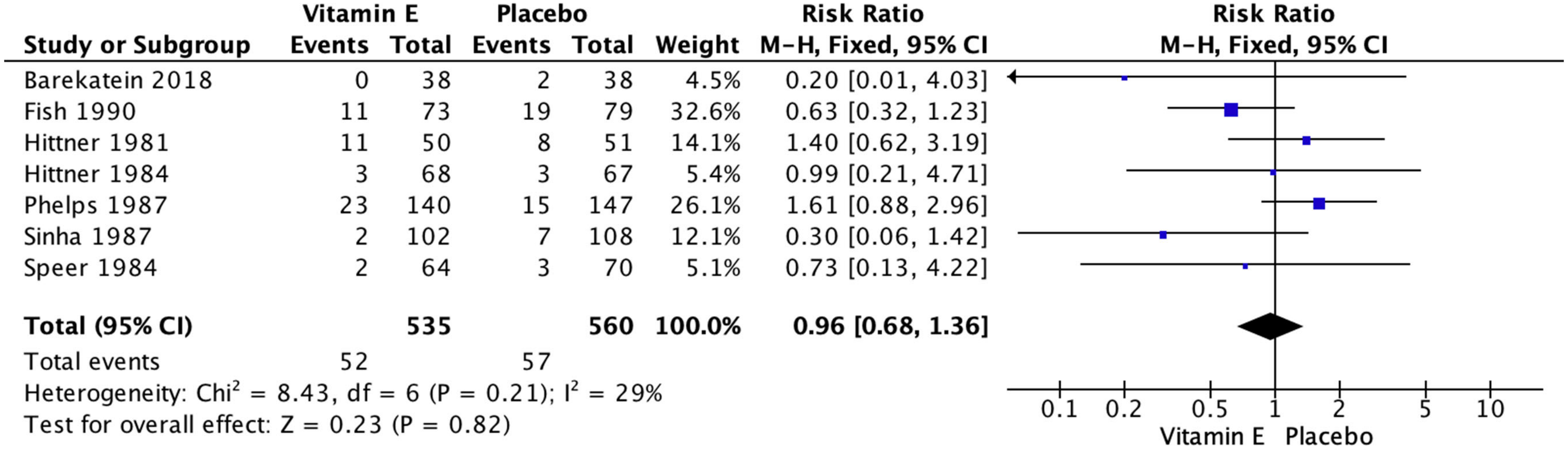

eFigure 41. Forest plot for comparison— Vitamin E supplementation vs. placebo for the outcome severe IVH.

© 2023 Razak A et al. *JAMA Network Open.*

Study or Subgroup	Erythropoietin Events	Erythropoietin Total	Placebo Events	Placebo Total	Weight	Risk Ratio M-H, Fixed, 95% CI
Fauchere 2015	4	229	3	214	4.0%	1.25 [0.28, 5.50]
Natalucci 2016	1	191	2	174	2.7%	0.46 [0.04, 4.98]
Song 2016	41	366	74	377	93.3%	0.57 [0.40, 0.81]
**Total (95% CI)**		**786**		**765**	**100.0%**	**0.59 [0.42, 0.83]**
Total events	46		79			

Heterogeneity: $Chi^2 = 1.05$, df = 2 (P = 0.59); $I^2 = 0\%$
Test for overall effect: Z = 3.01 (P = 0.003)

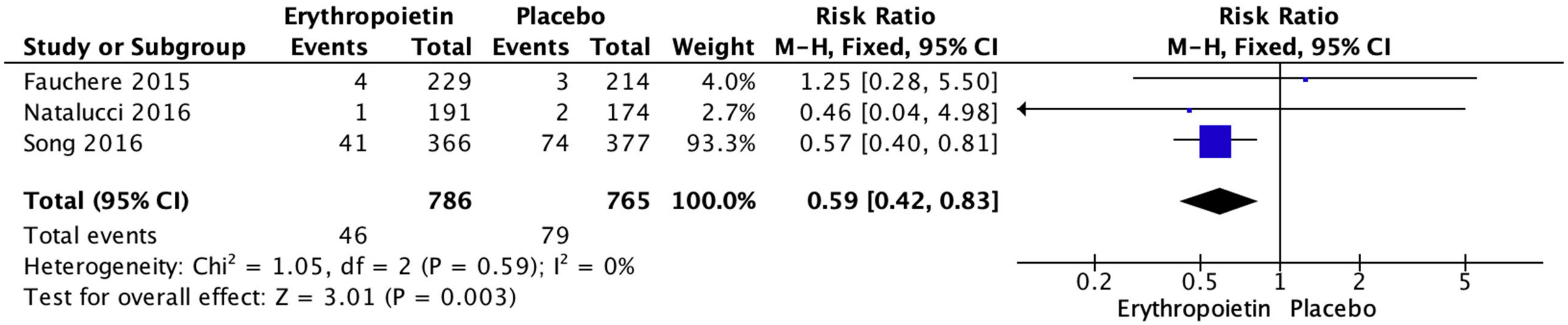

eFigure 42. Forest plot for comparison—Early erythropoiesis-stimulating agents vs. placebo for outcome cPVL

Study or Subgroup	Jet Ventillation Events	Jet Ventillation Total	Conventional Ventillation Events	Conventional Ventillation Total	Weight	Risk Ratio M-H, Fixed, 95% CI
Wiswell 1996	10	32	2	32	100.0%	5.00 [1.19, 21.04]
**Total (95% CI)**		**32**		**32**	**100.0%**	**5.00 [1.19, 21.04]**
Total events	10		2			

Heterogeneity: Not applicable
Test for overall effect: Z = 2.20 (P = 0.03)

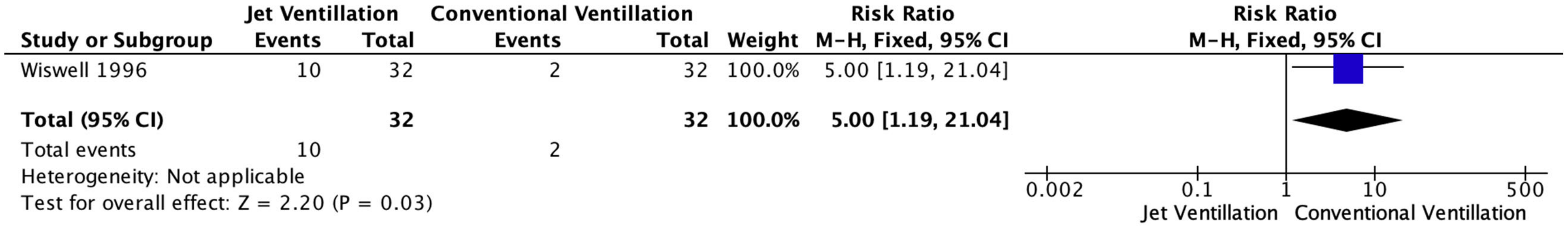

eFigure 43. Forest plot for comparison— Elective high-frequency jet ventilation vs. conventional ventilation method for the outcome cPVL.

Study or Subgroup	Any Steriods Events	Any Steriods Total	Placebo Events	Placebo Total	Weight	Risk Ratio M-H, Fixed, 95% CI
Peltoniemi 2007	2	159	0	167	100.0%	5.25 [0.25, 108.51]
**Total (95% CI)**		**159**		**167**	**100.0%**	**5.25 [0.25, 108.51]**
Total events	2		0			

Heterogeneity: Not applicable
Test for overall effect: Z = 1.07 (P = 0.28)

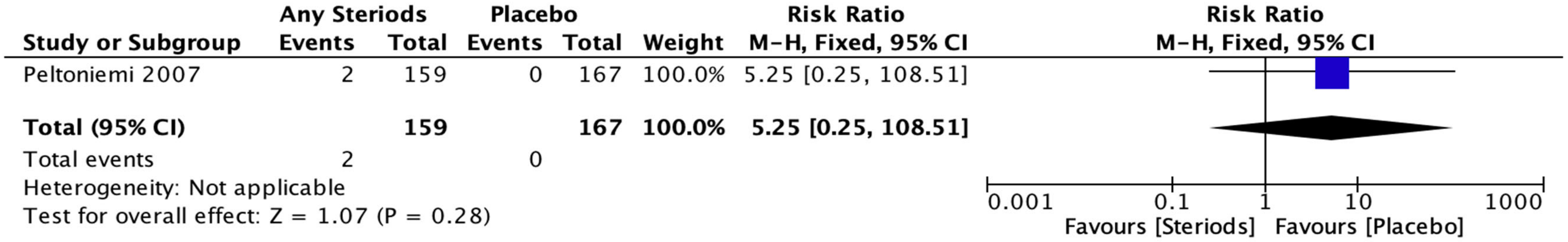

eFigure 44. Forest plot for comparison—Any steroids for lung maturity vs. placebo for the outcome cPVL.

© 2023 Razak A et al. *JAMA Network Open.*

Study or Subgroup	Betamethasone Events	Betamethasone Total	Dexamethasone Events	Dexamethasone Total	Weight	Risk Ratio M-H, Fixed, 95% CI
Chen 2005	1	81	2	76	100.0%	0.47 [0.04, 5.07]
**Total (95% CI)**		**81**		**76**	**100.0%**	**0.47 [0.04, 5.07]**
Total events	1		2			
Heterogeneity: Not applicable						
Test for overall effect: Z = 0.62 (P = 0.53)						

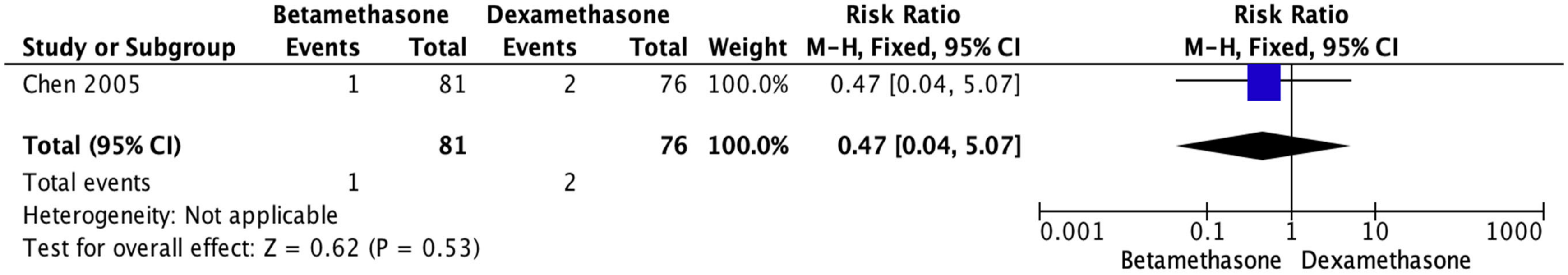

eFigure 45. Forest plots for comparison—Betamethasone for lung maturity vs. Betamethasone for lung maturity for outcome cPVL.

Study or Subgroup	Repeat Course Events	Repeat Course Total	Single Course Events	Single Course Total	Weight	Risk Ratio M-H, Fixed, 95% CI
Guinn 2001	2	248	3	235	22.5%	0.63 [0.11, 3.75]
Murphy 2008	9	1164	10	1140	73.9%	0.88 [0.36, 2.16]
Peltoniemi 2007	2	159	0	167	3.6%	5.25 [0.25, 108.51]
**Total (95% CI)**		**1571**		**1542**	**100.0%**	**0.98 [0.46, 2.07]**
Total events	13		13			
Heterogeneity: Chi^2 = 1.47, df = 2 (P = 0.48); I^2 = 0%						
Test for overall effect: Z = 0.05 (P = 0.96)						

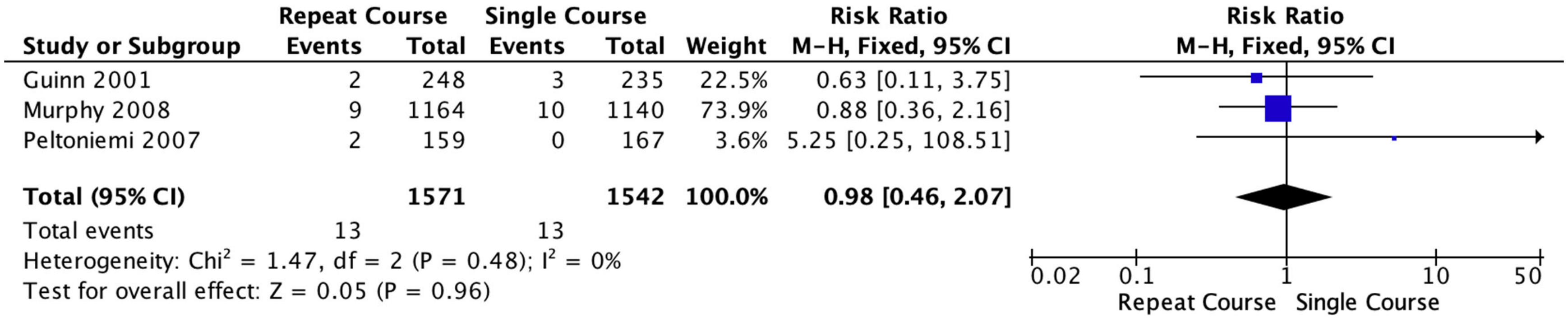

eFigure 46. Forest plot for comparison—Antenatal steroid for lung maturity: repeat course vs. single course for outcome cPVL.

© 2023 Razak A et al. *JAMA Network Open.*

Study or Subgroup	MgSO4 Events	MgSO4 Total	Placebo Events	Placebo Total	Weight	Risk Ratio M-H, Fixed, 95% CI
Crowther 2003	22	620	21	615	42.3%	1.04 [0.58, 1.87]
Marret 2006	27	341	28	324	57.7%	0.92 [0.55, 1.52]
**Total (95% CI)**		**961**		**939**	**100.0%**	**0.97 [0.66, 1.42]**
Total events	49		49			

Heterogeneity: $Chi^2 = 0.10, df = 1 (P = 0.75); I^2 = 0\%$

Test for overall effect: Z = 0.16 (P = 0.87)

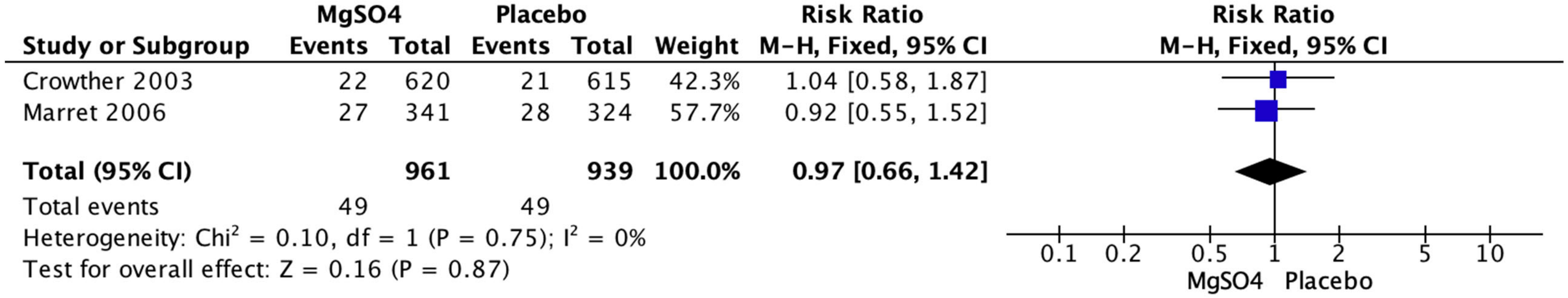

eFigure 47. Forest plot for comparison—Antenatal MgSO4 therapy for Neuroprotection vs. placebo for the outcome cPVL.

Study or Subgroup	Fio2 < 0.4 Events	Fio2 < 0.4 Total	Fio2 > 0.4 Events	Fio2 > 0.4 Total	Weight	Risk Ratio M-H, Fixed, 95% CI
Kapadia 2013	0	44	1	44	100.0%	0.33 [0.01, 7.97]
Lundstrom 1995	0	34	0	35		Not estimable
**Total (95% CI)**		**78**		**79**	**100.0%**	**0.33 [0.01, 7.97]**
Total events	0		1			

Heterogeneity: Not applicable

Test for overall effect: Z = 0.68 (P = 0.50)

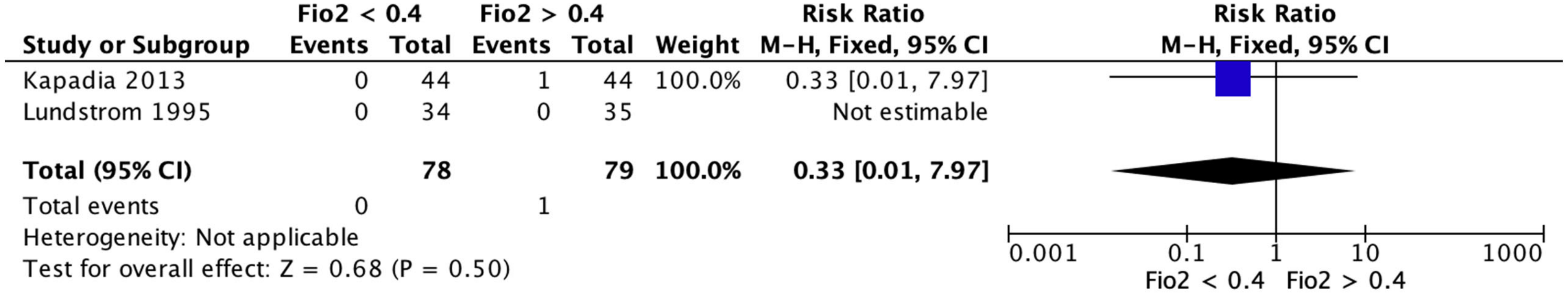

eFigure 48. Forest plot for comparison—lower FiO2 for resuscitation vs. higher FiO2 for resuscitation for the outcome cPVL.

© 2023 Razak A et al. *JAMA Network Open.*

Study or Subgroup	Sustained Inflation Events	Sustained Inflation Total	Standard Resuscitation Events	Standard Resuscitation Total	Weight	Risk Ratio M-H, Fixed, 95% CI
Jiravisitkul 2017	1	43	1	38	8.4%	0.88 [0.06, 13.65]
Linder 2005	2	31	4	30	32.3%	0.48 [0.10, 2.45]
Lista 2015	1	148	5	143	40.4%	0.19 [0.02, 1.63]
Nagan 2017	1	76	2	86	14.9%	0.57 [0.05, 6.12]
Schwaberger 2015	2	20	0	20	4.0%	5.00 [0.26, 98.00]
**Total (95% CI)**		**318**		**317**	**100.0%**	**0.59 [0.24, 1.44]**
Total events	7		12			

Heterogeneity: $Chi^2 = 3.17$, df = 4 (P = 0.53); $I^2 = 0\%$
Test for overall effect: Z = 1.16 (P = 0.25)

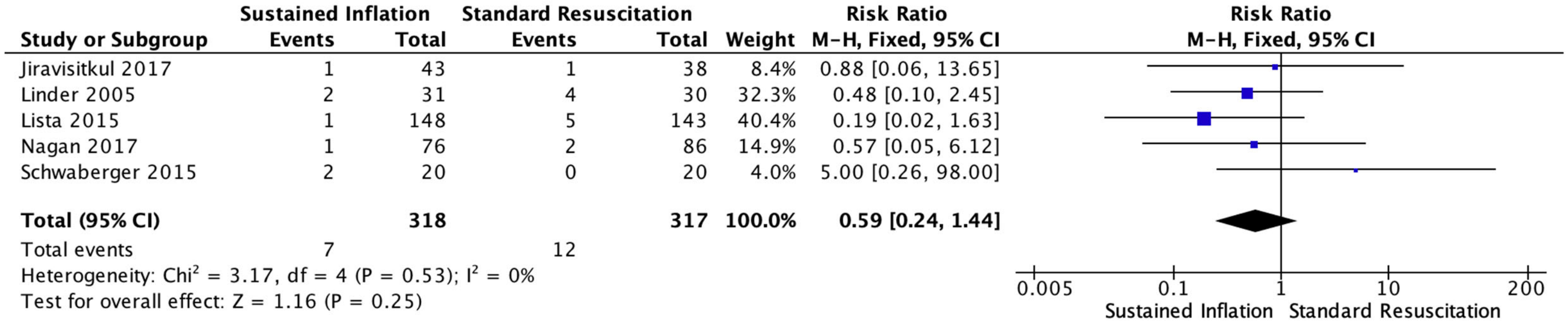

eFigure 49. Forest plot for comparison—Sustained inflation during resuscitation vs. standard resuscitation for the outcome cPVL.

Study or Subgroup	Cord Milking Events	Cord Milking Total	ECC Events	ECC Total	Weight	Risk Ratio M-H, Fixed, 95% CI
Hosono 2008	1	20	2	20	100.0%	0.50 [0.05, 5.08]
**Total (95% CI)**		**20**		**20**	**100.0%**	**0.50 [0.05, 5.08]**
Total events	1		2			

Heterogeneity: Not applicable
Test for overall effect: Z = 0.59 (P = 0.56)

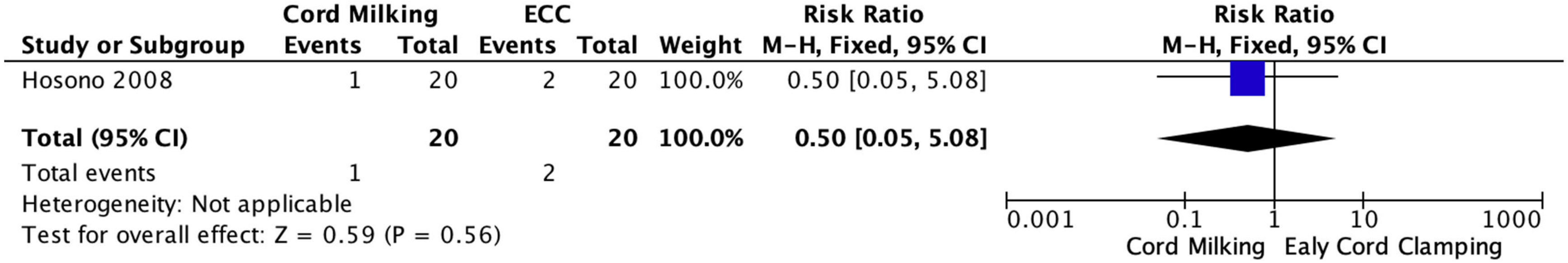

eFigure 50. Forest plot for comparison—Umbilical cord milking vs. early cord clamping for the outcome cPVL.

Study or Subgroup	Supine head Midline Events	Supine head Midline Total	Supine head Rotated Events	Supine head Rotated Total	Weight	Risk Ratio M-H, Fixed, 95% CI
Al Abdi 2011	1	23	0	25	100.0%	3.25 [0.14, 76.01]
Kochan 2019	0	90	0	90		Not estimable
**Total (95% CI)**		**113**		**115**	**100.0%**	**3.25 [0.14, 76.01]**
Total events	1		0			

Heterogeneity: Not applicable
Test for overall effect: Z = 0.73 (P = 0.46)

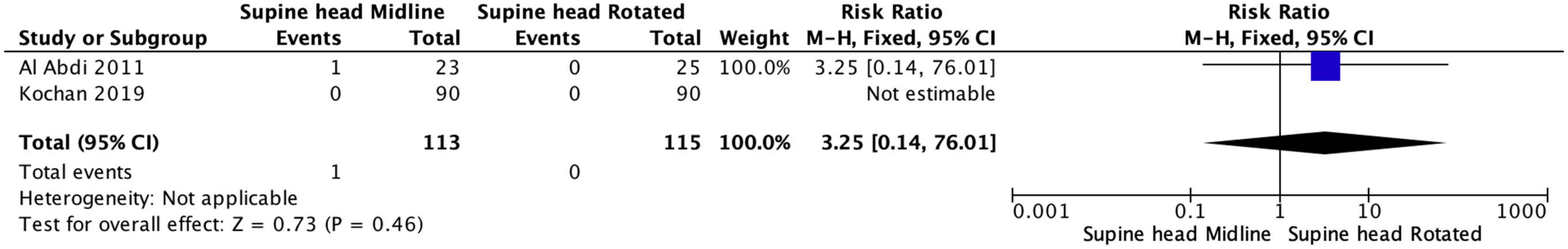

eFigure 51. Forest plot for comparison— supine head midline vs. supine head rotated for the outcome cPVL.

© 2023 Razak A et al. *JAMA Network Open.*

Study or Subgroup	LISA Events	LISA Total	INSURE Events	INSURE Total	Weight	Risk Ratio M-H, Fixed, 95% CI
Gopel 2011	5	108	2	112	15.0%	2.59 [0.51, 13.08]
Kribs 2015	4	107	11	104	85.0%	0.35 [0.12, 1.07]
**Total (95% CI)**		**215**		**216**	**100.0%**	**0.69 [0.30, 1.57]**
Total events	9		13			

Heterogeneity: $Chi^2 = 3.96$, df = 1 (P = 0.05); $I^2 = 75\%$
Test for overall effect: Z = 0.89 (P = 0.38)

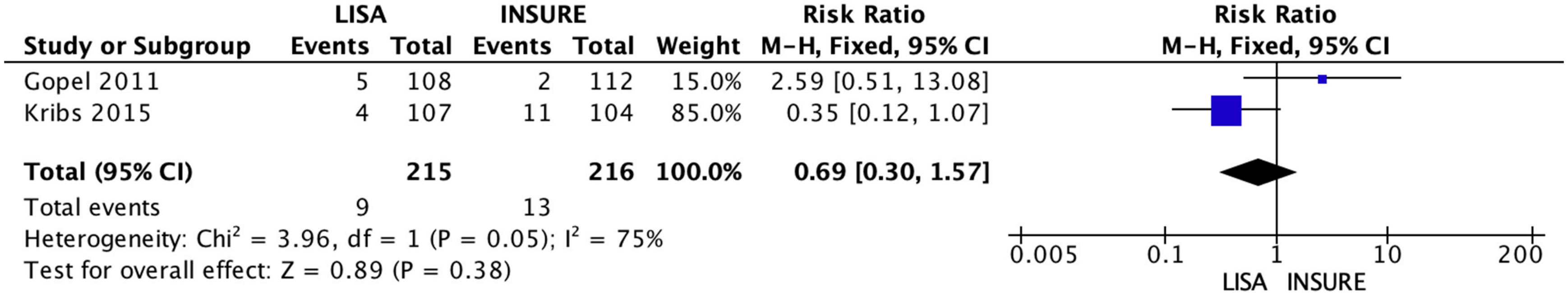

eFigure 52. Forest plot for comparison— LISA vs. INSURE method for surfactant administration for the outcome severe cPVL.

Study or Subgroup	Volume Target Events	Volume Target Total	Pressure Limit Events	Pressure Limit Total	Weight	Risk Ratio M-H, Fixed, 95% CI
Chowdhury 2013	1	20	0	20	100.0%	3.00 [0.13, 69.52]
**Total (95% CI)**		**20**		**20**	**100.0%**	**3.00 [0.13, 69.52]**
Total events	1		0			

Heterogeneity: Not applicable
Test for overall effect: Z = 0.69 (P = 0.49)

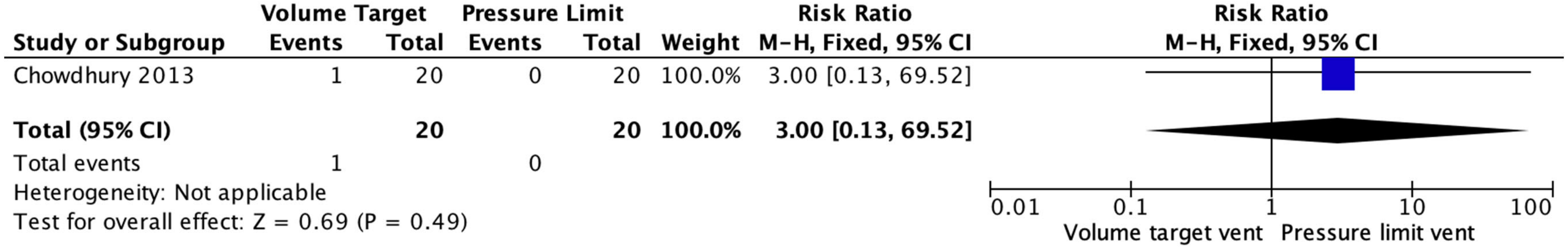

eFigure 53. Forest plot for comparison— Volume target ventilation vs. pressure limited ventilation for the outcome severe cPVL.

© 2023 Razak A et al. *JAMA Network Open.*

Study or Subgroup	High Frequency Vent Events	High Frequency Vent Total	Conventional Vent Events	Conventional Vent Total	Weight	Risk Ratio M-H, Fixed, 95% CI
Courtney 2002	18	244	26	254	56.0%	0.72 [0.41, 1.28]
Durand 2001	3	24	2	24	4.4%	1.50 [0.27, 8.19]
Johnson 2002	11	390	17	392	37.3%	0.65 [0.31, 1.37]
Plavka 1999	2	19	1	18	2.3%	1.89 [0.19, 19.13]
**Total (95% CI)**		**677**		**688**	**100.0%**	**0.76 [0.49, 1.16]**
Total events	34		46			
Heterogeneity: $Chi^2 = 1.42$, df = 3 (P = 0.70); $I^2 = 0\%$						
Test for overall effect: Z = 1.28 (P = 0.20)						

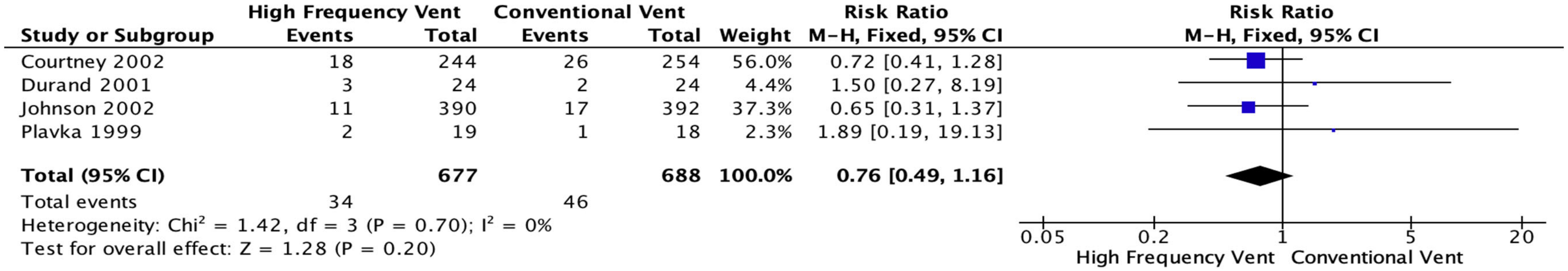

eFigure 54. Forest plot for comparison— Elective HFOV vs. conventional ventilation method for the outcome cPVL.

Study or Subgroup	High Oxygen Saturation Events	High Oxygen Saturation Total	Low Oxygen Saturation Events	Low Oxygen Saturation Total	Weight	Risk Ratio M-H, Fixed, 95% CI
Voucher (SUPPORT Trial) 2010	30	641	24	631	100.0%	1.23 [0.73, 2.08]
**Total (95% CI)**		**641**		**631**	**100.0%**	**1.23 [0.73, 2.08]**
Total events	30		24			
Heterogeneity: Not applicable						
Test for overall effect: Z = 0.77 (P = 0.44)						

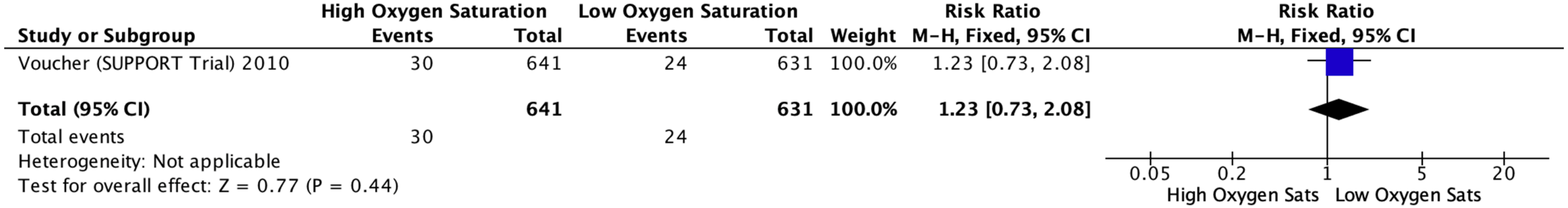

eFigure 55. Forest plot for comparison— high oxygen saturation target (91-95%) vs. low oxygen saturation target (85-89%) in NICU for the outcome cPVL.

Study or Subgroup	Permissive hypercapnea Events	Permissive hypercapnea Total	Normal PCO2 Events	Normal PCO2 Total	Weight	Risk Ratio M-H, Fixed, 95% CI
Mariani 1999	2	24	2	25	100.0%	1.04 [0.16, 6.81]
**Total (95% CI)**		**24**		**25**	**100.0%**	**1.04 [0.16, 6.81]**
Total events	2		2			
Heterogeneity: Not applicable						
Test for overall effect: Z = 0.04 (P = 0.97)						

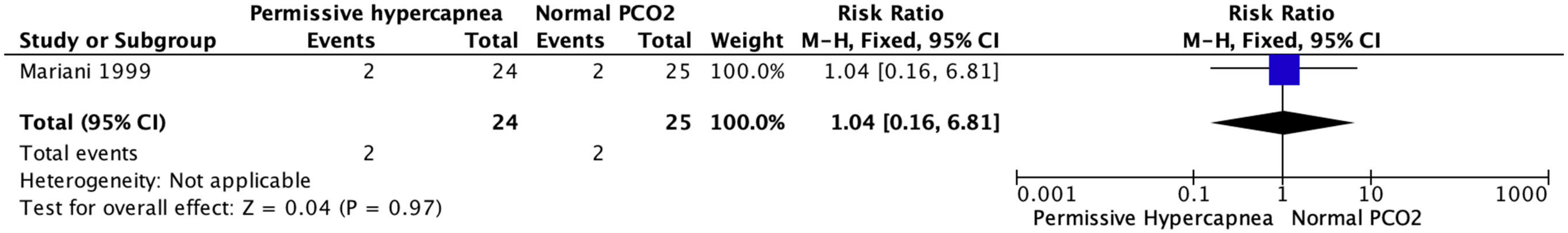

eFigure 56. Forest plot for comparison— permissive hypercapnia vs normocapnia during ventilation for the outcome cPVL.

© 2023 Razak A et al. *JAMA Network Open.*

Study or Subgroup	Caffeine Events	Caffeine Total	Placebo Events	Placebo Total	Weight	Risk Ratio M-H, Fixed, 95% CI
Cap Trial 2006	24	967	37	966	100.0%	0.65 [0.39, 1.07]
**Total (95% CI)**		**967**		**966**	**100.0%**	**0.65 [0.39, 1.07]**
Total events	24		37			
Heterogeneity: Not applicable						
Test for overall effect: Z = 1.68 (P = 0.09)						

Risk Ratio M-H, Fixed, 95% CI

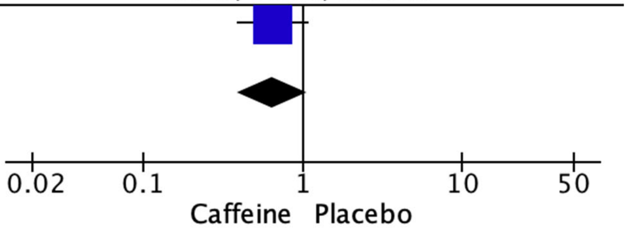

eFigure 57. Forest plot for comparison—Caffeine prophylaxis or treatment for apnea or post-extubation vs. placebo for the outcome cPVL.

Study or Subgroup	High Dose Caffiene Events	High Dose Caffiene Total	Standard Dose Caffeine Events	Standard Dose Caffeine Total	Weight	Risk Ratio M-H, Fixed, 95% CI
Gray 2010	5	120	11	126	100.0%	0.48 [0.17, 1.33]
**Total (95% CI)**		**120**		**126**	**100.0%**	**0.48 [0.17, 1.33]**
Total events	5		11			
Heterogeneity: Not applicable						
Test for overall effect: Z = 1.41 (P = 0.16)						

Risk Ratio M-H, Fixed, 95% CI

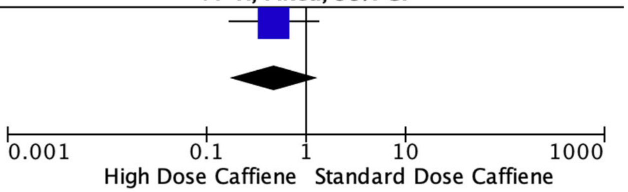

eFigure 58. Forest plot for comparison—high dose caffeine prophylaxis for apnea or post-extubation vs. low dose caffeine for the outcome cPVL.

Study or Subgroup	Opiods Events	Opiods Total	Placebo Events	Placebo Total	Weight	Risk Ratio M-H, Fixed, 95% CI
Anand 2004	27	367	34	367	100.0%	0.79 [0.49, 1.29]
**Total (95% CI)**		**367**		**367**	**100.0%**	**0.79 [0.49, 1.29]**
Total events	27		34			
Heterogeneity: Not applicable						
Test for overall effect: Z = 0.93 (P = 0.35)						

Risk Ratio M-H, Fixed, 95% CI

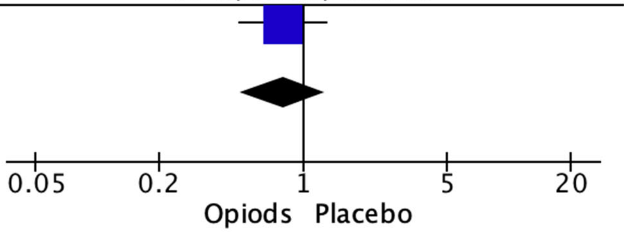

eFigure 59. Forest plot for comparison— Sedation during ventilation: opioid vs. placebo for the outcome cPVL.

© 2023 Razak A et al. *JAMA Network Open.*

Study or Subgroup	Muscle Relaxant Events	Muscle Relaxant Total	Placebo Events	Placebo Total	Weight	Risk Ratio M-H, Random, 95% CI	Risk Ratio M-H, Random, 95% CI
Shaw 1993	5	96	4	97	100.0%	1.26 [0.35, 4.56]	
**Total (95% CI)**		**96**		**97**	**100.0%**	**1.26 [0.35, 4.56]**	
Total events	5		4				
Heterogeneity: Not applicable							
Test for overall effect: Z = 0.36 (P = 0.72)							

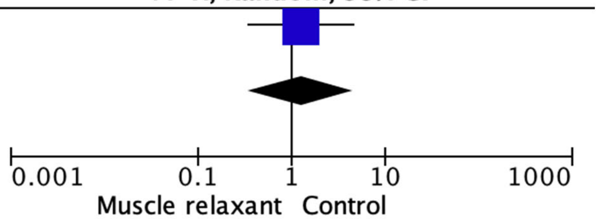

eFigure 60. Forest plot for comparison— Neuromuscular paralysis during ventilation vs. placebo for the outcome cPVL.

Study or Subgroup	Indomethacin Events	Indomethacin Total	Placebo Events	Placebo Total	Weight	Risk Ratio M-H, Fixed, 95% CI	Risk Ratio M-H, Fixed, 95% CI
Bandastra 1988	1	99	4	100	34.2%	0.25 [0.03, 2.22]	
Couser 1996	3	43	8	47	65.8%	0.41 [0.12, 1.45]	
**Total (95% CI)**		**142**		**147**	**100.0%**	**0.36 [0.12, 1.06]**	
Total events	4		12				
Heterogeneity: $Chi^2 = 0.14$, df = 1 (P = 0.70); $I^2 = 0\%$							
Test for overall effect: Z = 1.86 (P = 0.06)							

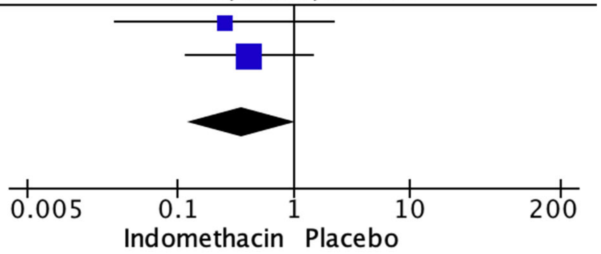

eFigure 61. Forest plot for comparison— Indomethacin prophylaxis for PDA vs. placebo for the outcome cPVL.

Study or Subgroup	Indomethacin Events	Indomethacin Total	Placebo Events	Placebo Total	Weight	Risk Ratio M-H, Fixed, 95% CI	Risk Ratio M-H, Fixed, 95% CI
Kluckow 2014	4	44	4	48	100.0%	1.09 [0.29, 4.10]	
**Total (95% CI)**		**44**		**48**	**100.0%**	**1.09 [0.29, 4.10]**	
Total events	4		4				
Heterogeneity: Not applicable							
Test for overall effect: Z = 0.13 (P = 0.90)							

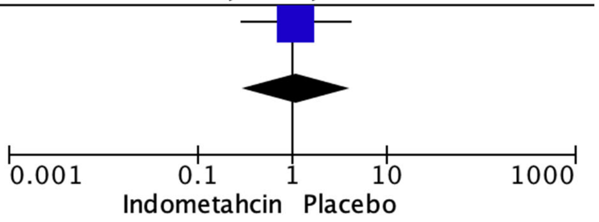

eFigure 62. Forest plot for comparison— Indomethacin pre-symptomatic treatment for PDA vs. placebo for the outcome cPVL.

© 2023 Razak A et al. *JAMA Network Open.*

Study or Subgroup	Ibuprofen Events	Ibuprofen Total	Placebo Events	Placebo Total	Weight	Risk Ratio M-H, Fixed, 95% CI
Dani 2005	3	77	4	78	25.1%	0.76 [0.18, 3.28]
Gournay 2004	6	65	7	66	43.8%	0.87 [0.31, 2.45]
Van Overmeire 2004	10	205	5	210	31.1%	2.05 [0.71, 5.89]
**Total (95% CI)**		**347**		**354**	**100.0%**	**1.21 [0.63, 2.30]**
Total events	19		16			

Heterogeneity: $Chi^2 = 1.73$, df = 2 (P = 0.42); $I^2 = 0\%$

Test for overall effect: Z = 0.58 (P = 0.56)

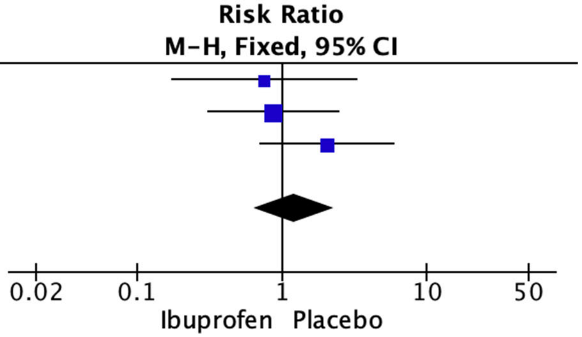

eFigure 63. Forest plot for comparison— Ibuprofen prophylaxis for PDA vs. placebo for the outcome cPVL.

Study or Subgroup	Liberal transfusion Events	Liberal transfusion Total	Restrictive transfusion Events	Restrictive transfusion Total	Weight	Risk Ratio M-H, Fixed, 95% CI
Bell 2005	0	51	4	49	13.5%	0.11 [0.01, 1.93]
Franz 2020	22	491	25	516	71.7%	0.92 [0.53, 1.62]
Kirpalani 2006	8	228	5	223	14.9%	1.56 [0.52, 4.71]
**Total (95% CI)**		**770**		**788**	**100.0%**	**0.91 [0.56, 1.47]**
Total events	30		34			

Heterogeneity: $Chi^2 = 3.04$, df = 2 (P = 0.22); $I^2 = 34\%$

Test for overall effect: Z = 0.39 (P = 0.70)

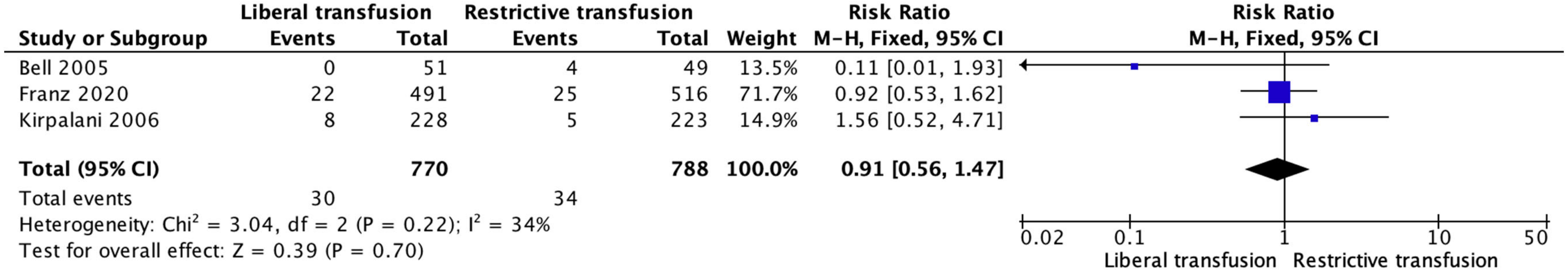

eFigure 64. Forest plot for comparison— Restrictive vs. liberal packed red cell transfusion for anemia for the outcome cPVL.

© 2023 Razak A et al. *JAMA Network Open.*

Study or Subgroup	HFOV Events	HFOV Total	Conventional Ventilation Events	Conventional Ventilation Total	Weight	Risk Ratio M-H, Fixed, 95% CI
Courtney 2002	50	244	59	254	41.6%	0.88 [0.63, 1.23]
Durand 2001	3	24	4	24	2.9%	0.75 [0.19, 3.00]
Johnson 2002	54	393	75	393	54.0%	0.72 [0.52, 0.99]
Plavka 1999	2	19	2	18	1.5%	0.95 [0.15, 6.03]
**Total (95% CI)**		**680**		**689**	**100.0%**	**0.79 [0.63, 0.99]**
Total events	109		140			

Heterogeneity: $Chi^2 = 0.78$, df = 3 (P = 0.85); $I^2 = 0\%$
Test for overall effect: Z = 2.02 (P = 0.04)

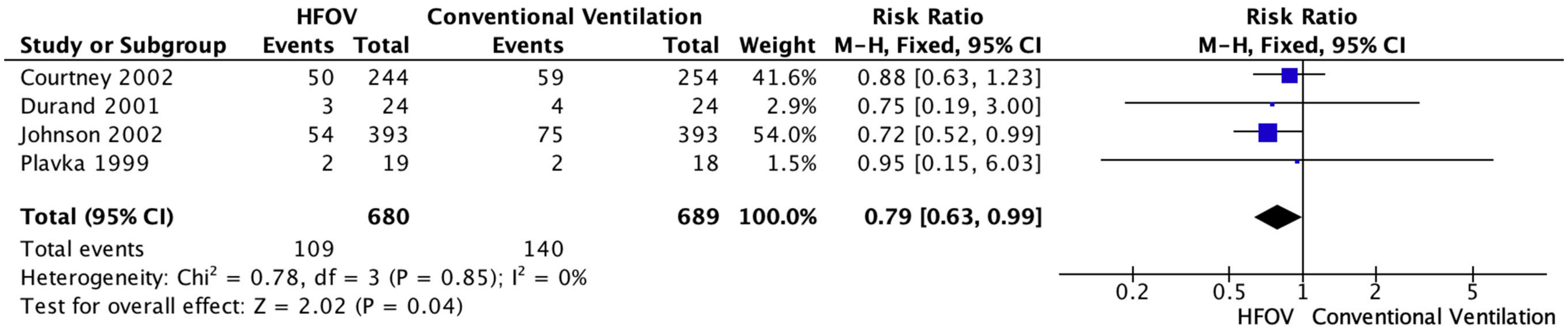

eFigure 65. Forest plot for comparison— Elective HFOV vs. conventional ventilation method for the outcome severe brain injury.

Study or Subgroup	Antenatal Steriods Events	Antenatal Steriods Total	Placebo Events	Placebo Total	Weight	Risk Ratio M-H, Fixed, 95% CI
The WHO Action Trials 2020	6	810	3	720	100.0%	1.78 [0.45, 7.08]
**Total (95% CI)**		**810**		**720**	**100.0%**	**1.78 [0.45, 7.08]**
Total events	6		3			

Heterogeneity: Not applicable
Test for overall effect: Z = 0.82 (P = 0.41)

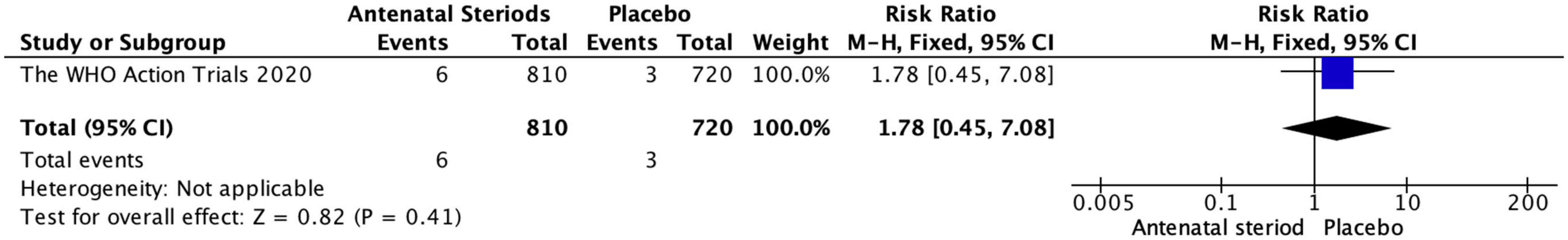

eFigure 66. Forest plot for comparison—Any steroids for lung maturity vs. placebo for the outcome severe brain injury

Study or Subgroup	DCC Events	DCC Total	ECC Events	ECC Total	Weight	Risk Ratio M-H, Fixed, 95% CI
Hemmati 2020	6	810	3	720	6.6%	1.78 [0.45, 7.08]
Tarnow Mordi 2017	51	691	45	684	93.4%	1.12 [0.76, 1.65]
**Total (95% CI)**		**1501**		**1404**	**100.0%**	**1.16 [0.80, 1.69]**
Total events	57		48			

Heterogeneity: $Chi^2 = 0.40$, df = 1 (P = 0.53); $I^2 = 0\%$
Test for overall effect: Z = 0.80 (P = 0.42)

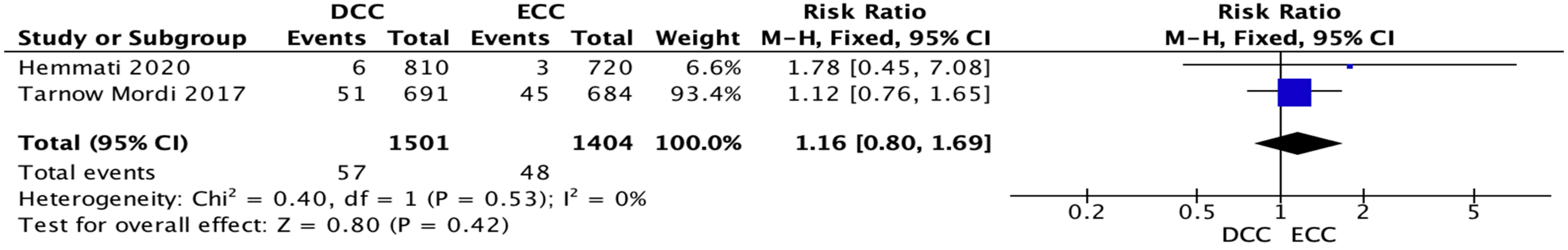

eFigure 67. Forest plot for comparison—Delayed cord clamping vs. early cord clamping for the outcome severe brain injury

© 2023 Razak A et al. *JAMA Network Open.*

Study or Subgroup	High Oxy Sats Events	High Oxy Sats Total	Low Oxy Sats Events	Low Oxy Sats Total	Weight	Risk Ratio M-H, Fixed, 95% CI
BOOST-II Australia 2016	49	512	37	506	14.8%	1.31 [0.87, 1.97]
BOOST-II NZ 2014	15	160	21	161	8.3%	0.72 [0.38, 1.34]
BOOST-II UK 2016	49	410	56	412	22.3%	0.88 [0.61, 1.26]
Schmidt (COT trial) 2013	123	598	136	589	54.6%	0.89 [0.72, 1.11]
**Total (95% CI)**		**1680**		**1668**	**100.0%**	**0.94 [0.80, 1.10]**
Total events	236		250			
Heterogeneity: Chi² = 3.59, df = 3 (P = 0.31); I² = 16%						
Test for overall effect: Z = 0.80 (P = 0.42)						

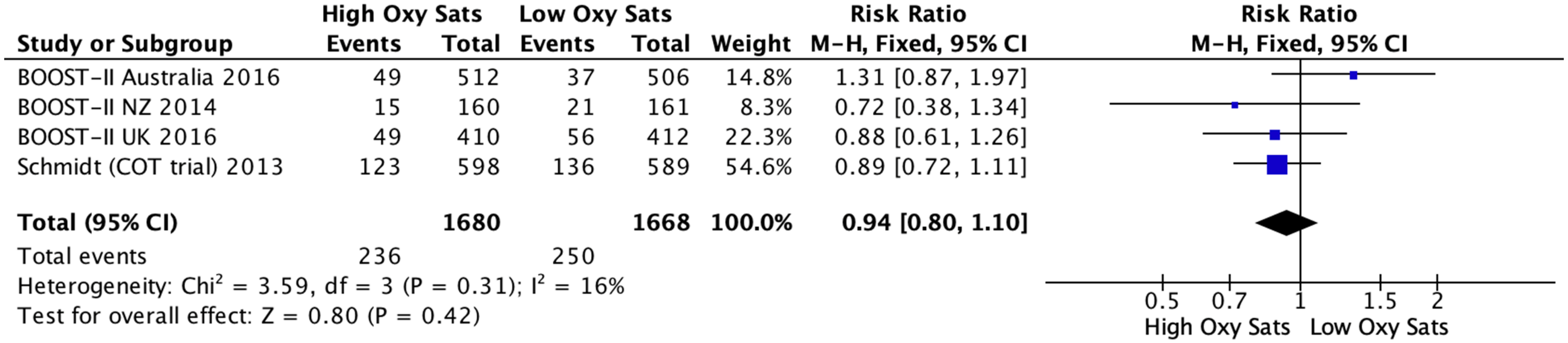

eFigure 68. Forest plot for comparison— High vs. low oxygen saturation targets for the outcome severe brain injury

Study or Subgroup	Caffeine Events	Caffeine Total	Placebo Events	Placebo Total	Weight	Risk Ratio M-H, Fixed, 95% CI
Cap Trial 2006	126	967	138	966	100.0%	0.91 [0.73, 1.14]
**Total (95% CI)**		**967**		**966**	**100.0%**	**0.91 [0.73, 1.14]**
Total events	126		138			
Heterogeneity: Not applicable						
Test for overall effect: Z = 0.80 (P = 0.42)						

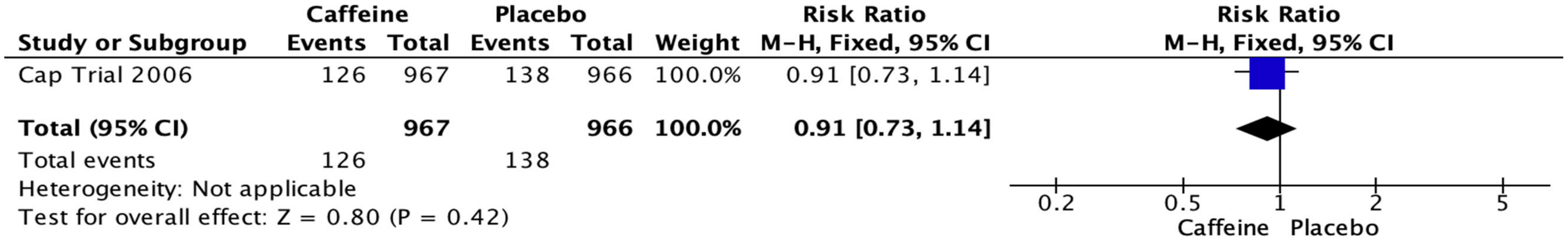

eFigure 69. Forest plot for comparison-Caffeine prophylaxis or treatment for apnea/post-extubation for outcome severe brain injury

Study or Subgroup	Indomethacin Events	Indomethacin Total	Placebo Events	Placebo Total	Weight	Risk Ratio M-H, Fixed, 95% CI
Couser 1996	6	43	12	47	7.5%	0.55 [0.22, 1.33]
Schmidt 2001	125	563	142	562	92.5%	0.88 [0.71, 1.08]
**Total (95% CI)**		**606**		**609**	**100.0%**	**0.85 [0.70, 1.05]**
Total events	131		154			
Heterogeneity: Chi² = 1.04, df = 1 (P = 0.31); I² = 4%						
Test for overall effect: Z = 1.52 (P = 0.13)						

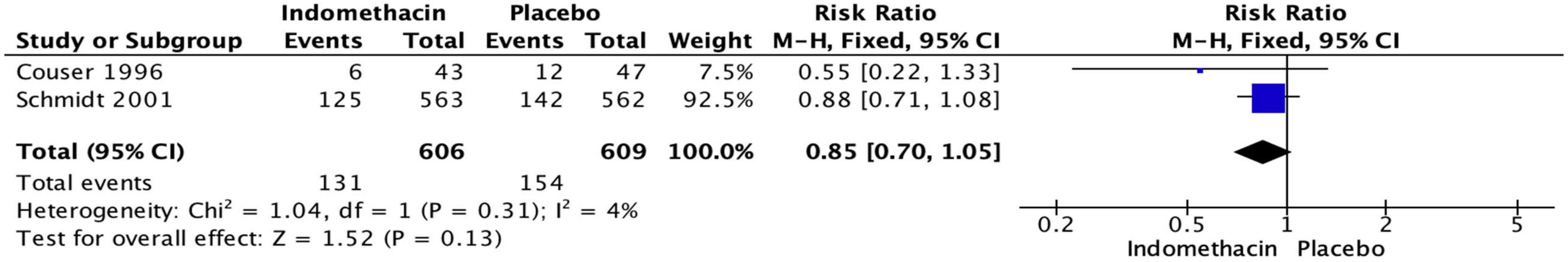

eFigure 70. Forest plot for comparison— Indomethacin prophylaxis for the outcome severe brain injury

© 2023 Razak A et al. *JAMA Network Open.*

Study or Subgroup	Liberal Events	Liberal Total	Restrictive Events	Restrictive Total	Weight	Risk Ratio M-H, Fixed, 95% CI
Bell 2005	0	51	6	49	3.5%	0.07 [0.00, 1.28]
Kirpalani 2006	32	218	30	216	15.8%	1.06 [0.67, 1.68]
Kirpalani 2020	146	855	154	859	80.7%	0.95 [0.78, 1.17]
**Total (95% CI)**		**1124**		**1124**	**100.0%**	**0.94 [0.78, 1.13]**
Total events	178		190			

Heterogeneity: $Chi^2 = 3.33$, $df = 2$ ($P = 0.19$); $I^2 = 40\%$
Test for overall effect: $Z = 0.67$ ($P = 0.50$)

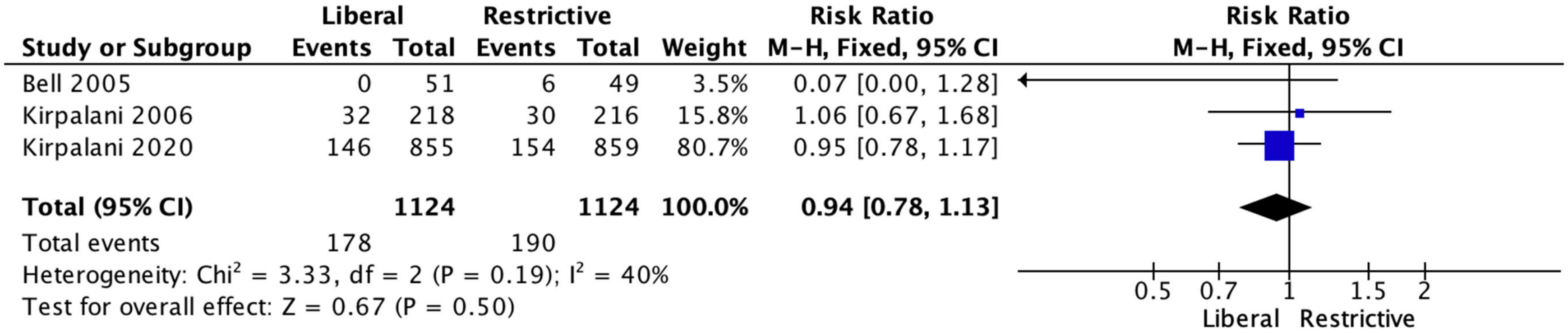

eFigure 71. Forest plot for comparison— Restrictive vs. liberal packed red cell transfusion for anemia for the outcome severe brain injury

© 2023 Razak A et al. *JAMA Network Open.*
