# Supplementary material for: Interventions to Reduce Severe Brain Injury Risk in Preterm Neonates: A Systematic Review and Meta-analysis
Source: JAMA Netw Open. 2023 Apr 13;6(4):e237473. doi: 10.1001/jamanetworkopen.2023.7473 (PMC10102877; doi:10.1001/jamanetworkopen.2023.7473)
Supplement: Supplement 2. — Data Sharing Statement [file jamanetwopen-e237473-s002.pdf]

Data Sharing Statement

Razak. Interventions to Reduce Severe Brain Injury Risk in Preterm Neonates. *JAMA Netw Open*. Published April 13, 2023. doi:10.1001/jamanetworkopen.2023.7473

Data
Data available: Yes
Data types: Other (please specify)
Additional Information: all applicable data is uploaded
How to access data: all applicable data is uploaded
When available: With publication

Supporting Documents
Document types: Other (please specify)
Additional Information: all applicable data is uploaded
How to access documents: all applicable data is uploaded
When available: With publication

Additional Information
Who can access the data: all applicable data is uploaded
Types of analyses: all applicable data is uploaded
Mechanisms of data availability: all applicable data is uploaded
Any additional restrictions: all applicable data is uploaded
